# EGFR-T790M Mutation–Derived Interactome Rerouted EGFR Translocation Contributing to Gefitinib Resistance in Non-Small Cell Lung Cancer

**DOI:** 10.1016/j.mcpro.2023.100624

**Published:** 2023-07-24

**Authors:** Pei-Shan Wu, Miao-Hsia Lin, Jye-Chian Hsiao, Pei-Yi Lin, Szu-Hua Pan, Yu-Ju Chen

**Affiliations:** 1 Genome and Systems Biology Degree Program, National Taiwan University, Taipei, Taiwan; 2 Institute of Chemistry, Academia Sinica, Taipei, Taiwan; 3 Department of Microbiology, National Taiwan University College of Medicine, Taipei, Taiwan; 4 Graduate Institute of Medical Genomics and Proteomics, National Taiwan University College of Medicine, Taipei, Taiwan; 5 Doctoral Degree Program of Translational Medicine, National Taiwan University College of Medicine, Taipei, Taiwan; 6 Department of Chemistry, National Taiwan University, Taipei, Taiwan

**Keywords:** epidermal growth factor receptor, non-small cell lung cancer, tyrosine kinase inhibitor, T790M mutation, TKI-resistance, protein-protein interaction, autophagy

## Abstract

Secondary mutation, T790M, conferring tyrosine kinase inhibitors (TKIs) resistance beyond oncogenic epidermal growth factor receptor (EGFR) mutations presents a challenging unmet need. Although TKI-resistant mechanisms are intensively investigated, the underlying responses of cancer cells adapting drug perturbation are largely unknown. To illuminate the molecular basis linking acquired mutation to TKI resistance, affinity purification coupled mass spectrometry was adopted to dissect EGFR interactome in TKI-sensitive and TKI-resistant non-small cell lung cancer cells. The analysis revealed TKI-resistant EGFR-mutant interactome allocated in diverse subcellular distribution and enriched in endocytic trafficking, in which gefitinib intervention activated autophagy-mediated EGFR degradation and thus autophagy inhibition elevated gefitinib susceptibility. Alternatively, gefitinib prompted TKI-sensitive EGFR translocating toward cell periphery through Rab7 ubiquitination which may favor efficacy to TKIs suppression. This study revealed that T790M mutation rewired EGFR interactome that guided EGFR to autophagy-mediated degradation to escape treatment, suggesting that combination therapy with TKI and autophagy inhibitor may overcome acquired resistance in non-small cell lung cancer.

## Highlights

• Illuminating EGFR-mutant interactome in TKI-sensitive and TKI-resistant NSCLC cells.
• T790M rewired EGFR interactome and shifted EGFR trafficking routes.
• Gefitinib-induced autophagy directs EGFR degradation in TKI-resistant NSCLC cells.
• EGFR recycling *via* Rab7 ubiquitination favors efficacy to TKIs suppression.

## Graphical Abstract

**Figure:**
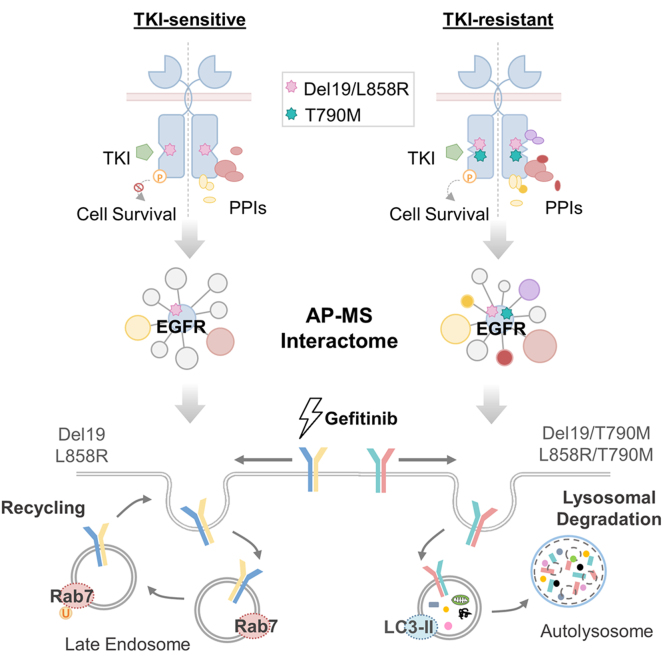

## In Brief

Given the high relapse of TKI resistance caused by EGFR secondary mutation, T790M, EGFR mutant interactomes are constructed in four patient-derived NSCLC cells. The T790M rewired EGFR interactome in diverse subcellular localization and shifted EGFR trafficking routes. The gefitinib treatment activated autophagy pathway in TKI-resistant cells, instead of EGFR recycling *via* Rab7 ubiquitination in TKI-sensitive cells. This first functional link between the mutation-mediated PPI and drug resistance behind the long-standing clinical burden may provide opportunities to develop novel therapeutic strategies.

Lung cancer is the most common and leading cause of cancer-related mortality worldwide (1, 2). Non-small cell lung cancer (NSCLC) is the most common type, accounting for 85% of lung cancers. At molecular level, activating mutations in epidermal growth factor receptor (EGFR) are the most frequent genetic mutation driving cancer cell proliferation and tumor malignancy in East Asians never-smoker NSCLC patients, especially in females (3, 4). Among these mutations, small in-frame deletions of exon 19 (Del19) and L858R point mutation in exon 21 (L858R) are the two predominant EGFR mutations accounting for about 90% of EGFR mutations, which leads to ligand-independent EGFR activation (5) and most widely used EGFR tyrosine kinase inhibitors (TKIs)-based targeted therapy (6). Though first and second generation of EGFR TKIs (gefitinib, erlotinib, and afatinib) show favorable clinical outcome (7), up to 70% of patients eventually develop TKI resistance and tumor relapses upon 9 to 15 months of TKI treatment (8). The most common cause of TKI resistance came from the occurrence of secondary EGFR mutation at the “gatekeeper” position 790 (T790M) in exon 20 (9), approximately contributing to 60% of the acquired resistance NSCLC cases (10, 11). Despite the third-generation TKI, osimertinib, has been designed on the purpose to overcome the acquired TKI resistance EGFR T790M mutation (12), its efficacy can be compromised by another acquired mutation C797S as well as other unknown routes (13, 14). Though it has been known that these resistant EGFR mutations lead to preventing osimertinib binding, it merely takes around 22% of all mechanisms to acquire third-generation TKI resistance. The current study aims to explore the alternative mechanism targeting T790M, the first and most frequent mutation for nearly all patients receiving the first line of EGFR therapy, such as gefitinib, which is a long-standing burden in the health reimbursement system of East Asia countries. The aberrant activation of alternative or unknown bypass pathways still play the major mechanisms of resistant occurrence, their molecular mechanism, and how the mutation affects the EGFR-mediated oncogenesis is not clear. Given this dilemma in treating NSCLC lung cancer, there is an unmet need to develop alternative therapies either targeting active EGFR mutations without acquiring resistance or treating tumors with acquired secondary mutations.

The process of signal transduction is highly dependent on specific protein-protein interactions (PPIs), and identification of the PPI components is imperative to gain insight into the molecular pathogenesis of causal proteins (15, 16). Disease-associated oncogenic mutations have been reported to affect short linear motifs in the intrinsic disorder regions of proteins, resulting in aberrant PPIs to rewire downstream signaling networks (17, 18). Affinity purification coupled mass spectrometry (AP-MS) offered comprehensive identification of endogenous PPI components, referred as interactome, in cell lines or tissues (19, 20). For instance, Lundby *et al.* (21) utilized AP-MS to characterize EGF-dependent phosphotyrosine signaling network in lung tissue, revealing that oncogenic mutation EGFR P1019L switched the interaction components and subsequently led to cell migration and invasiveness. AP-MS also provides a system view of EGFR-PPI, including a 14-protein core network crucial for cell viability in multiple EGFR-mutated lung cancer cells (22), and a protein phosphatases interactome to clarify EGFR signaling (23). These EGFR interactome studies, mainly focusing on primary EGFR mutation (Del19 or L858R) or mutation-independent TKI resistance, extend the knowledge of the molecular basis that PPI acts as critical switches to determine the oncogenic fate and nominate new therapeutic targets to regulate EGFR internalization, protein turnover, and cell migration (24, 25). The molecular mechanism on how acquired secondary T790M mutation perturbs the EGFR-PPI that contributes to rewire the downstream signaling under TKI resistance remains to be further elucidated.

In this study, we aimed to study the molecular pathogenicity of TKI resistance induced by EGFR T790M mutation. Specifically, AP-MS was applied to dissect the EGFR PPI dynamics on four NSCLC cell lines, which harbor primary mutation (Del19 or L858R) and secondary mutation (Del19/T790M or L858R/T790M). The EGFR interactome of TKI-resistant NSCLC cells was found to allocate in different subcellular localization and is highly enriched in protein trafficking. Further validation revealed that autophagy was hyperactive in response to gefitinib treatment in TKI-resistant models (CL68 and H1975), which prompted EGFR translocating into lysosomes for degradation and thus contributing to TKI tolerance. On the contrary, in TKI-sensitive models (PC9 and H3255), the activated pathway of late endosome (LE), which carries EGFR recycle back to plasma membrane through Rab7 ubiquitination, may provide good response to successful TKI administration. Here, we demonstrated that TKI induced distinct EGFR trafficking routes due to the different PPI network caused by secondary T790M mutation, which may offer an alternatively therapeutic opportunity to overcome TKI resistance by targeting EGFR endocytic trafficking.

## Experimental Procedures

### Cell Culture

The H3255 (ATCC CRL-2882) and H1975 (ATCC CRL-5908) cell lines were obtained from American Type Culture Collection, the PC9 cell line was a kind gift from Dr James Chih-Hsin Yang (National Taiwan University Cancer Center), and CL68 cell line was obtained from Dr Chao-Chi Ho’s laboratory (National Taiwan University Hospital). Among these cell models, H3255 (L858R) and PC9 (Del19) are gefitinib-sensitive, and H1975 (L858R/T790M) and CL68 (Del19/T790M) are gefitinib-resistant. All cells were maintained at 37 °C in humidified air with 5% CO2 and in RPMI1640 medium supplemented with 10% fetal bovine serum, 100 units/ml penicillin, 100 μg/ml streptomycin, and 250 ng/ml amphotericin (all from Gibco; Thermo Fisher Scientific, Inc).

### Reagents and Antibodies

Protease inhibitor cocktail (EDTA-Free) was purchased from Merck. Phosphatase inhibitor cocktail 2/3 was purchased from Sigma-Aldrich. Bis[sulfosuccinimidyl]suberate and Protein G agarose beads were obtained from Thermo Fisher Scientific. Gefitinib and cetuximab were purchased from Selleckchem. The PI3K inhibitors (3-methyladenine, 3-MA), ATPase inhibitor (bafilomycin A1, BafA1), proteasome inhibitor (MG-132), and caspase inhibitor (Z-VAD-FMK) were all purchased from Selleckchem.

The primary antibodies for immunoblotting and immunofluorescence are shown as follows: EGFR (#4267), pY1173-EGFR (#4407), Rab7 (#9367), LC3 (#4108), EEA1 (#3288), Rab11 (#5589), pS473-AKT (#4060), AKT (#4691), ubiquitin (#3936), mTOR (#2972), pS2448-mTOR (#2971) were obtained from Cell Signaling Technology. Rab5 (166622), β-actin (47778), EGFR (373746) were purchased from Santa Cruz Biotechnology. Rab7A (55469-1-AP), ubiquitin (10201-2-AP), and RILP (p62, 13574-1-AP) were obtained from ProteinTech. NEDD4L (A302-513A) and VTA1 (A304-828A) were purchased from Bethyl Laboratories, Inc. Rab7 (ab50533) for Western blot of Rab7-IP was purchased from Abcam. Normal mouse immunoglobulin G (IgG) and rabbit IgG were obtained from Santa Cruz (sc-2025) and Millipore (12-370), respectively, which were used as negative control for immunoprecipitation (IP).

Gefitinib was dissolved in dimethyl sulfoxide to produce a 10 mM stock solution at −20 °C. 3-MA was prepared as a 50 mM solution in RPMI medium and filtered with 0.22 μm filter before use. BafA1, Z-VAD-FMK, and MG-132 were dissolved in dimethyl sulfoxide to make a stock solution with concentration at 160 μM, 20 mM, and 20 mM at −20 °C, respectively.

### IP of EGFR Complex

The EGFR interactome analysis was conducted using four lung cancer cell lines, PC9, CL68, H3255, and H1975 cell lines. The AP-MS experiments were performed for three biological replicates of each cell line, and duplicate liquid chromatography-tandem mass spectrometry (LC-MS/MS) runs were conducted for each sample. The spectral count from these six replicates was summed up (n = 6) for following nonspecific-binding elimination and bioinformatics analysis as described in the session of “Exclusion of Nonspecific-Binding Proteins”. The cultured NSCLC cells were washed with ice-cold PBS and lysed on dish with lysis buffer (150 mM NaCl, 100 mM sodium phosphate, 0.5% NP-40, 10% glycerol) with phosphatase and protease inhibitors. The cell lysates was agitated by rotation under 4 °C for 30 min and centrifuged with 12,600*g* in 4 °C for 30 min. The supernatants were collected and quantified protein concentrations with bicinchoninic acid assay kit (BCA, Thermo Fisher Scientific) using bovine serum albumin as the standard. Before cell lysis, antibodies (cetuximab or normal mouse IgG) were conjugated on protein G agarose beads and cross-linked with 2 mM bis[sulfosuccinimidyl]suberate in 20 mM Hepes buffer (100 mM NaCl, pH7.4) under 4 °C for overnight. The preconjugated antibody-beads were washed three times with lysis buffer and centrifuged with 1500 rpm for 5 min to remove unconjugated antibodies. The cell lysatees were then incubated with preconjugated antibody-beads in 4 °C overnight. Beads were collected by centrifugation and washed with enough volume of lysis buffer and tris-buffered saline buffer to remove unbinding proteins and detergents. Elution of interacting protein complexes was achieved by the addition of 0.1 M glycine (pH 1.5). Eluates were neutralized with 1 M triethylammonium bicarbonate buffer (pH8.5) before enzymatic digestion. For immunoassay, 2-fold concentration of SDS sample buffer was used to elute protein complexes from beads followed by 10 min incubation at 95 °C. The eluents were separated by SDS-PAGE for Western blotting and detected by antibody staining.

### Protein Digestion

Protein complexes were subsequently reduced with 10 mM DTT for 30 min and alkylated with 50 mM iodoacetamide for 45 min under 37 °C. Samples were digested with Lys-C (Wako) for 3 h and trypsin (Promega) under 37 °C for overnight; the enzyme-to-protein ratio for Lys-C was 1:100 (w/w) and trypsin was 1:50 (w/w). Digested peptides were acidified with 10% TFA to a final concentration of 0.5% (pH ∼2–3) and desalted by the homemade StageTip plugged with 3 M Empore Styrene divinylbenzene (SDB-XC) disk. Peptide samples were stored at −80 °C until LC-MS/MS analysis.

### Liquid Chromatography-Tandem Mass Spectrometry

Peptide samples were dried with vacuum centrifugation and dissolved in 0.1% formic acid (FA). LC-MS/MS profiling were acquired on a TripleTOF 5600 MS system (AB SCIEX) coupled with a nanoACQUITY Ultra Performance LC (Waters Corporation). The LC separation was performed with 15 cm home-made capillary column packing with 3 μm ReproSil-Pur 120 C18-AQ particles (Dr Maisch). The mobile phase of LC system consisted of water with 0.1% FA (buffer A) and acetonitrile with 0.1% FA (buffer B). Peptides were separated through a 90 min gradient of up to 85% buffer B at a flow rate of 500 nl min^−1^. Mass spectrometry (MS) was operated in ESI positive mode, and the ion spray source parameters were as follow: ion spray voltage of 2.5 kV, nebulizer gas at 20 psi, curtain gas at 15 psi, and interface heater temperature of 150 °C. For information-dependent acquisition, the data was acquired for 350 ms and the MS survey range was *m/z* 350 to 1250. The top 15 precursor ions with charge state +2 to +4, of which the threshold exceeded 100 counts per second in each MS survey, were selected for subsequent fragmentation. The MS/MS scan was performed for 100 ms in each acquisition, and the dynamics exclusion was set for 12 s. The collision energy was adjusted automatically using the rolling CE function of Analyst TF software (https://sciex.com/products/software/analyst-tf-software).

### Protein Identification and Quantification

The MS raw data files were converted to the open mzXML file format and further processed using the *Trans*-Proteomic Pipeline (v.4.9.0) suite of tools. For protein identification, X!Tandem search engine was used against UniProt human database containing 20,205 protein entries (downloaded on Dec. 15, 2015) with the mass tolerance of 20 ppm and 0.1 Da for parent ions and fragment ions, respectively, and the maximum of missed cleavage was 2. Carbamidomethyl cysteine was set as fixed modification (C, +57.0214 Da) and methionine oxidation (M, +15.995 Da), acetylation (protein N-term, +42.011 Da) as variable modifications. The false discovery rate filtration performed at 1% was both done on peptide and protein level. The ProteinProphet posterior protein probability was set between 0.5∼0.99. The protein quantification was calculated by Abacus based on spectral counting, which is built-in protein length normalization to eliminate the bias against the number of detectable peptides (26). Subsequently, the sum of spectral count was subjected to calculate the confidence of protein interaction using SAINT statistical model at the SAINT probability threshold ≥0.99.

### Exclusion of Nonspecific-Binding Proteins

The nonspecific-binding proteins were excluded by using IgG antibody as a control. In this study, we adapted stringent criteria for protein identification. Thus, the proteins identified based on one unique peptide among all datasets were eliminated. The proteins with EGFR/IgG ratio ≥5 computed by spectral count in at least 1 cell of the paired model (PC9 *versus* CL68 or H3255 *versus* H1975) were retained for the following analysis in both paired cell lines. The filtered interactors were then mapped to the contaminant repository for affinity purification (CRAPome) database (27) and a community benchmarked work surveying the antibody quality for IP (28) to extract the contaminant frequency, in which the contaminant frequency was defined as the detection percentage of all AP-MS purifications using negative control. The frequency extracted from CRAPome (F_CRAPome_) and benchmarking reference (F_ab_ and F_IgG_) were averaged and applied as a filter requirement with threshold ≤10, this representing the protein had 10% chance to be identified as a contaminant, to eliminate the nonspecific binders that cannot be removed from up-front reduction using ratio of EGFR/IgG. The remaining proteins were considered as *bona fide* EGFR interactors for the follow-up analysis.

### Bioinformatic Analysis of EGFR Interactome

The scatter plot of technical reproducibility and hierarchical clustering of confident interactors were performed with Perseus software (v1.5.0.15, https://maxquant.org/perseus/). PPI networks were constructed by STRING (v10.0, https://string-db.org/). The confidence cutoff for showing interaction links was set to medium (0.400), and all items of the various evidence types in STRING were selected, such as experiments, databases, and co-expression, and contributed to the network construction. The visualization and network features analysis were processed by Cytoscape (v3.3.0, https://cytoscape.org/). The functional annotation of protein identified in the EGFR interactome was classified with the Database for Annotation, Visualization and Integrated Discovery (DAVID 6.8, https://david.ncifcrf.gov/). The enrichment of biological processes and annotation terms were performed by gene ontology. The signaling pathways of interacting proteins were mapped to the KEGG (Kyoto Encyclopedia of Genes and Genomes) and Reactome (v3.6, https://reactome.org/) database.

### Western Blotting Analysis

Cells were treated or cotreated with different inhibitors in immunoblotting assays: gefitinib (IC50/20 dosage), 3-MA (10 mM), BafA1 (0.1 μM), MG-132 (5 μM), and Z-VAD-FMK (50 μM). Cells were lysed on dish with PTS buffer (12 mM sodium deoxycholate, 12 mM sodium lauroyl sarcosine, and 100 mM Tris–HCl, pH9.0) with complete EDTA-free protease inhibitor cocktail (1:200, v/v) and phosphatase inhibitors cocktail 2 and 3 (1:100, v/v). Protein concentrations were measured by BCA assay. Equal amounts of protein from each sample were loaded into 10% or 15% SDS-polyacrylamide gel. After electrophoresis, separated proteins were transferred to polyvinylidene difluoride (0.45 μm pore size) membranes (PerkinElmer). Membranes were blocked with 5% bovine serum albumin in TBS containing 0.1% Tween-20, probed with indicated primary antibodies (1/1000–1/3000, v/v), detected with HRP-conjugated secondary antibodies (1/3000, v/v), visualized by enhanced chemiluminescent substrate (PerkinElmer), and imaged using ImageQuant LAS-4000 (FujiFilm). The quantitative densitometry of immunoblots was performed using ImageJ 1.49v (National Institutes of Health). Net LC3-II flux assay was calculated difference between treatment and control under same condition group and presented with densitometric units.

### Co-IP for Western Blotting Analysis

NSCLC cells were lysed with lysis buffer (0.5% NP-40, 10 mM Tris–HCl, 150 mM NaCl, pH7.5) with inhibitors of phosphatase, protease, or deubiquitinase (N-ethylmaleimide, 10 mM, Sigma-Aldrich). The cell lysate was agitated by rotation under 4 °C for 30 min and centrifuged with 12,600*g* in 4 °C for 30 min. The supernatants were quantified protein concentrations with BCA and incubated with respective antibodies by rotation at 4 °C for overnight. Prewashed protein G agarose beads were then added to supernatant and incubated by rotating at 4 °C for 1 h. Beads were collected by centrifugation and washed three times with lysis buffer and TBS buffer. Remove washing buffer completely, and 2X SDS sample buffer (contained 10 mM DTT) was added to the beads followed by 10 min incubation at 95 °C. Samples were separated by SDS-PAGE for Western blotting and detection by antibody staining (ubiquitin, 1:1000, CST#3936; Rab7, 1:1000, PT#55469-1-AP).

### Immunofluorescence and Confocal Microscopy

TKI-sensitive and TKI-resistant NSCLC cells were seeded at a cell density 1.5 × 10^5^ (PC9 and H1975) and 3.0 × 10^5^ (CL68 and H3255) cells ml^−1^ in 6 cm dish onto 6 mm glass coverslips and incubated for 48 h. After 18 h treatment of gefitinib (IC20 dosage), cells were washed with PBS and fixed with 3.7% (w/v) paraformaldehyde in PBS for 10 min and permeabilized with 0.1% Triton-X100 in PBS for 5 min at room temp. Cells were then incubated with indicated primary antibodies (EGFR, SC-373746; Rab7, CST#9367; LC3, CST#4108; diluted 1:100 in PBS) overnight at 4 °C, followed by incubation with secondary antibody (Invitrogen, anti-mouse AlexaFlour594, or anti-rabbit AlexaFlour488, diluted 1:200 in PBS) for 1 h at 37 °C in the dark. The rhodamine phalloidin (Invitrogen, Alexa Fluor594, 1:40 in PBS) was used under single primary antibody staining and incubated for 1 h at 37 °C in the dark. PBS was used to wash coverslips before mounting on microscope slides using ProLong Diamond antifade mounting oil with DAPI (Invitrogen). Confocal images were captured using ZEISS LSM 780 confocal microscopy (Zeiss) with x63 oil objective lenses and 1024 × 1024 frame. Laser power and gain were adjusted manually to optimize fluorescence intensity and each fluorophore with minimal photobleaching. Images were analyzed using ZEISS ZEN2011 software (https://www.zeiss.com/microscopy/en/products/software/zeiss-zen-lite.html), and the puncta numbers were calculated by Image J. Pearson correlation coefficient of colocalization analysis were obtained by using Coloc 2 plugin of Image J. The images selected to quantify were acquired randomly, and each region of interest was corresponded to every single cell, Pearson’s correlation coefficient between EGFR and LC3/Rab7 was defined.

### MTS Cell Proliferation Assay

Cell proliferation was analyzed by using CellTiter 96 Aqueous One Solution Cell Proliferation Assay (MTS) kit (Promega Corporation) according to the manufacturer's instructions. For combined treatment, cells were seeded in 12-well plate and incubated for 48 h. Once the cells were treated/cotreated with the chemicals for 18 h (IC20 gefitinib, 10 mM 3-MA, or 0.1 μM BafA1), the medium was replaced with mixed solution of MTS reagent and fresh medium (1:10) and then incubated for 1.5 h at 37 °C, 5% CO_2_ in a humidified incubator. The absorbance was measured at 490 nm using Multiskan Go (Thermo Fisher Scientific).

### Experimental Design and Statistical Rationale

The EGFR interactome analysis was conducted using four lung cancer cell lines, PC9, CL68, H3255, and H1975 cell lines. The AP-MS experiments were performed for three biological replicates of each cell line, and duplicate LC-MS/MS runs were conducted for each sample. For quantitative comparison to identify the interacting proteins, spectral count method was used to calculate and sum the number of spectra from these six replicates (n = 6) for following nonspecific-binding elimination and bioinformatics analysis as described above. For immunoassay, the experiments were executed for at least three independent experiments and presented as the bar plot with mean ± SD. The differences between sample groups were performed using Student’s *t* test to determine the statistical significance with the threshold of *p* value ≤0.05.

## Results

### Characterization of EGFR Interactomes in TKI-Sensitive and TKI-Resistant NSCLC Cells

**Fig. 1:**
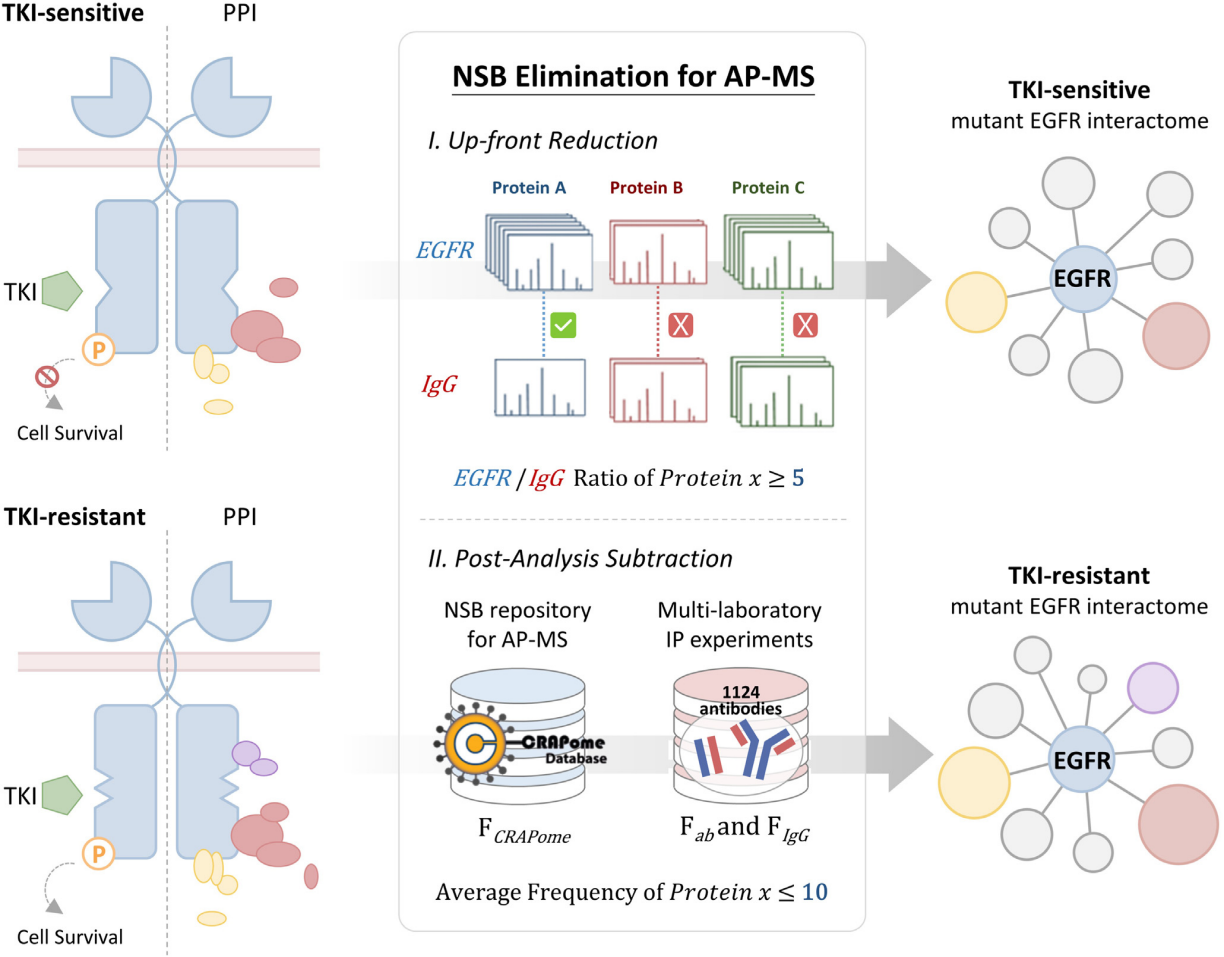
**Mutant EGFR interactome analysis derived from TKI-sensitive and TKI-resistant NSCLC cells.** Two distinct primary EGFR mutation–containing NSCLC cell lines, PC9 (Del19) and H3255 (L858R), and their secondary mutation counterparts, CL68 (Del19-T790M) and H1975 (L858R-T790M), represent as TKI-sensitive and TKI-resistant models, respectively. Protein complexes were pulled-down using cetuximab (EGFR) and IgG followed by proteolytic digestion and then subjected into LC-MS/MS analysis. Two data processing steps were employed to eliminate nonspecific interactors. In the up-front step (I), the contaminants were removed by comparing spectral count between IgG control (*IgG*) and cetuximab pull-down (*EGFR*) experiments. The threshold of spectral count ratio between EGFR and IgG was set as larger or equal to 5. In the post-analysis step (II), the common contaminants in AP-MS experiment were removed based on the detection frequency. The detection frequency was extracted from CRAPome database (F_*CRAPome*_). We filtered out the potential contaminants based on the frequency of detection across multiple different antibodies (F_*ab*_) and their congenital controls (F_*IgG*_), together with F_*CRAPome*_ to gain the highly confident EGFR-interacting proteins. The average frequency of detection was set as ≤10 representing that the false discovery rate is less than 0.1. The promising EGFR interactors were used to construct network topology and perform further bioinformatics analysis. AP-MS, affinity purification coupled mass spectrometry; CRAPome, contaminant repository for affinity purification; EGFR, epidermal growth factor receptor; IgG, immunoglobulin G; LC-MS/MS, liquid chromatography-tandem mass spectrometry; NSCLC, non-small cell lung cancer; TKI, tyrosine kinase inhibitor.

To dissect the underlying mechanisms of the TKI resistance conferred by secondary EGFR-T790M mutation, we applied AP-MS strategy to construct the EGFR interactome atlas in two TKI-sensitive NSCLC cell lines and their corresponding TKI-resistant NSCLC cell lines (Fig. 1). The EGFR interactome analysis was conducted using four lung cancer cell lines, PC9, CL68, H3255, and H1975 cell lines. The AP-MS experiments were performed for three biological replicates of each cell line, and duplicate LC-MS/MS runs were conducted for each sample. In brief, PC9 and H3255 NSCLC cell lines represent the gefitinib-sensitive NSCLCs which carry the constitutively activating EGFR mutations, Del19 and L858R, respectively. The CL68 and H1975 cell lines harbor both primary and additional secondary mutation, Del19/T790M or L858R/T790M, respectively, contributing to gefitinib-resistant phenotype (29, 30). The detailed histological and clinical information of NSCLC cell lines were listed in supplemental Table S1. Cetuximab, a monoclonal EGFR antibody approved as targeted therapy, was used as bait to pull-down the EGFR interactome due to its high affinity against the EGFR outer membrane domain III (25, 31, 32, 33), and mouse IgG was used as a control to exclude the nonspecifically-bound proteins. To minimize the effect caused by different EGFR expression, the EGFR expression was measured by immunoblot and the pulldown protein amount of EGFR was technically adjusted between paired cell models and evaluated with immunoblotting before MS-based interactome analysis (supplemental Fig. S1*A*). Relative protein abundance was quantitatively compared for both cetuximab and mouse IgG captured proteins by spectral count which is commonly utilized in label-free proteomics based on the number of peptides identified from a given protein (34). Furthermore, the nonspecific interactors copurified through binding to solid matrix were filtered out based on the comparative quantitation between cetuximab pull-down and IgG control and the criteria described on the AP-MS contaminant database (Fig. 1).

Based on AP-MS analysis, a total of 1138 proteins were found as potential members of the EGFR interactome under at least one cell model, in which 537, 653, 250, and 619 proteins were individually identified from PC9, CL68, H3255, and H1975, respectively (supplemental Table S2). The Pearson’s correlation coefficients of quantified protein showed good reproducibility in the biological triplicates of PC9 and CL68 AP-MS analyses but slightly lower in the pair of H3255 and H1975 experiments (supplemental Fig. S1*B*). Since the presence of nonspecific interactors is inevitable, a two-step scoring approach was adopted to distinguish true low-level interactors from background contaminants (Fig. 1). In the first step of up-front background reduction, the actual EGFR interactors were filtered with at least 5-fold higher abundance in cetuximab pull-down (*EGFR*) *versus* IgG control (*IgG*), calculated by the sum of spectral counts from biological replicates. Then, the average contaminant frequency (value ≤10) was further taken into account based on the information from the CRAPome database (F_*CRAPome*_) (27) and the multilaboratory IP experiments (F_*ab*_ and F_*IgG*_) (28). With this two-step filtering, a total of 187 highly confident EGFR interactors were identified, including 26 proteins commonly detected across four cell models and 13, 26, 2, and 43 proteins uniquely observed in PC9, CL68, H3255, and H1975 cells, respectively (supplemental Fig. S1*B*). Notably, the EGFR interactomes from the NSCLC cells harboring the same primary EGFR mutations showed high overlapping interactors; 34 and 23 were commonly identified in the Del19 and L858R EGFR mutation pairs, respectively (supplemental Fig. S1*B* and supplemental Tables S2–S5). Protein identified on the basis of single unique peptide was also included as potential EGFR interactors after elimination of non-specific binding proteins, if they show significant enrichment with criteria of EGFR/IgG ≥5 in the corresponding partner cells. The annotated spectra of these cases were provided to support the identification confidence in these four EGFR interactomes (supplemental Fig. S6). Furthermore, the cells with the similar oncogenic background were clustered, implying that different primary EGFR mutations had specific PPIs triggering distinct oncogenic pathways (supplemental Fig. S1*C*). These results, consistent with previous findings, indicated that oncogenic mutations of EGFR switch the components of the protein complex.

### EGFR-T790M–Orientated PPIs Suggest Diverse Spatial Interactome in TKI-Resistant NSCLC Cells

**Fig. 2:**
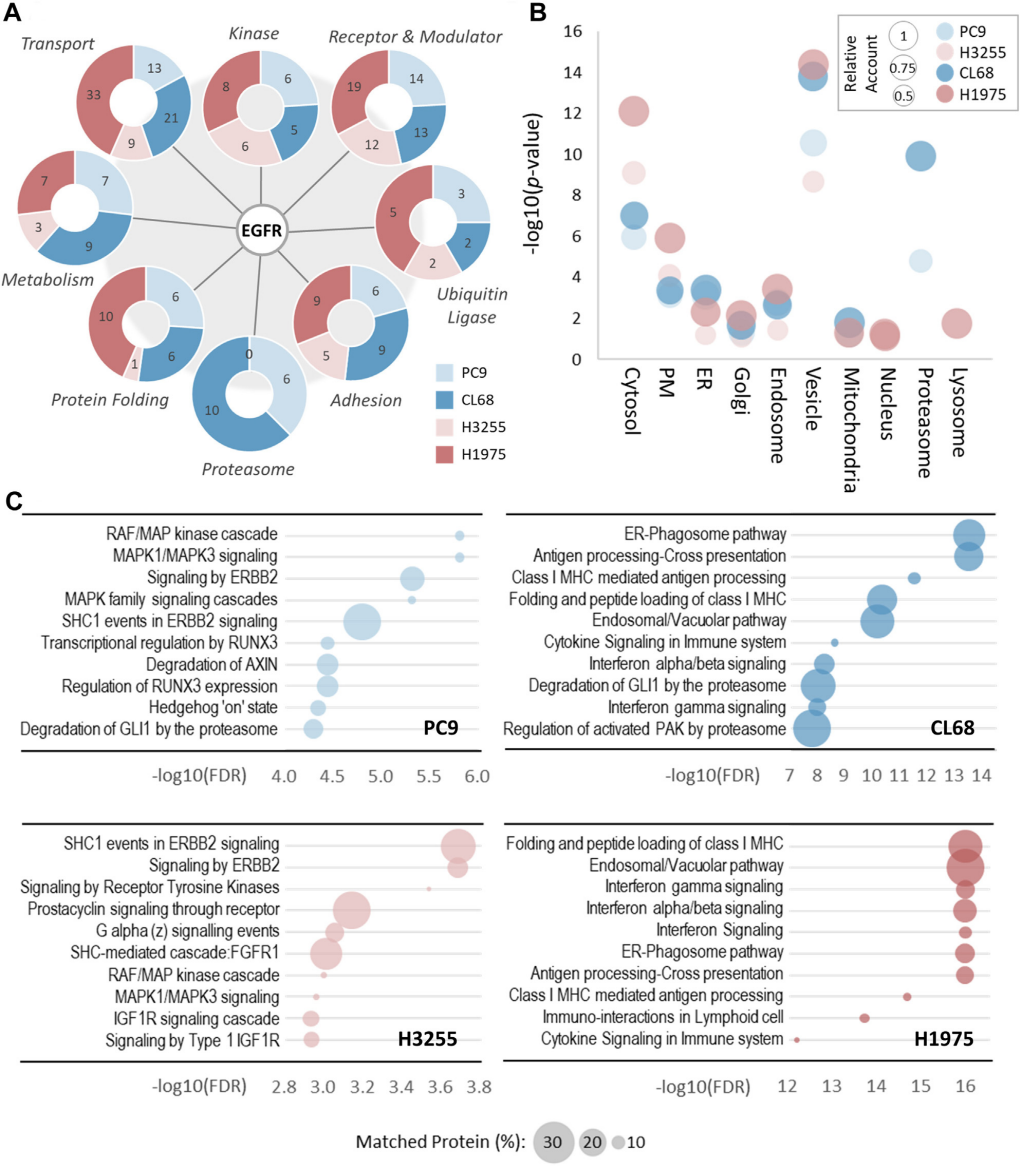
**Bioinformatics analysis characterizes EGFR interactomes in TKI-sensitive and TKI-resistant NSCLC cells.***A*, EGFR PPI topologies were constructed based on the highly confident interactors from STRING and presented with a schematic network. The interacting proteins were grouped together according to their protein functions and involved pathways. The matched protein number from each cell line models were indicated in the doughnut plot. *B*, the bubble diagram showed the annotated GO cellular component of EGFR interactomes in four cell lines. The enrichment score was presented with −log10(*p* value), and the bubble size means the relative protein count belongs to a specific subcellular compartment. *C*, pathway enrichment of EGFR interactomes was analyzed by Reactome, and the top ten pathways were presented with bubble plots. The X-axis shows the enrichment score of each pathway (−log10(FDR)), and the circle size represents the percentage of protein number involved in each pathway (%). The FDR ≤0.05 was set as the statistically significant threshold. EGFR, epidermal growth factor receptor; ER, endoplasmic reticulum; FDR, false discovery rate; GO, gene ontology; NSCLC, non-small cell lung cancer; PM, plasma membrane; PPI, protein-protein interaction; TKI, tyrosine kinase inhibitor.

The PPI networks of each mutated EGFR protein were then established with 62/178, 76/210, 39/83, and 92/217 node/edges based on the connectivity and the number of PPIs presented in topology for PC9, CL68, H3255, and H1975 NSCLC cells, respectively (supplemental Fig. S2). By gene ontology enrichment, these network nodes were clustered into eight subgroups, including transport, protein folding, adhesion, metabolism, kinase, receptor and modulator, proteasome, and ubiquitin ligase subgroups (Fig. 2*A*). In agreement with previous studies, the well-known EGFR interactors, ERBB2 and MET, were found as major hubs, which were frequently reported as a bypass mechanism for acquiring resistance (supplemental Fig. S2) (35). Intriguingly, most major hubs were commonly identified across four NSCLC cells, yet generally more PPI components were observed in the resistant CL68 and H1975 cells (Fig. 2*A*). We therefore deduced that these major hubs represented as the static EGFR interactors rendering the housekeeping processes for basic life support (36). These EGFR interactors which were enriched in the functional categories of transport, including protein, small molecule, and ion transportation, showed higher interacting multiplicity and intensity in both TKI-resistant cells than the TKI-sensitive models (Fig. 2*A*, “Transport”). This result implied that the acquired T790M mutation not only introduced EGFR structural change for eliminating TKI binding but also switched the EGFR PPIs towards cellular transporting.

We next examined the cellular compartments of the EGFR-T790M PPI. As expected, most EGFR interactors displayed similar organelle distributions in cytosol and plasma membrane across four NSCLC cell lines, while the proteasome-related proteins were only enriched in the NSCLC cells possessing EGFR Del19 mutations and the EGFR-L858R interacted with more nuclear and lysosomal proteins (Fig. 2*B*). Interestingly, the membrane-bound structures such as endosome and vesicle were significantly enriched in TKI-resistant CL68 and H1975 cells (Fig. 2*B*). Furthermore, pathway analysis showed that the oncogenic signaling pathways such as ERBB2/MAPK signaling and cell adhesion were commonly enriched in all NSCLC cells, in agreement with their crucial role in regulating cell proliferation, survival, as well as migration (Fig. 2*C*) (37). The ER-phagosome, endosomal/vacuolar pathway, and endocytosis were specifically overrepresented in TKI-resistant models (Fig. 2*C*, right panel), again, emphasizing the significance of protein intracellular trafficking in T790M acquired–resistant NSCLC cells. Endocytosis is a key mechanism for internalizing cell surface molecules and surface-bound cargos (38), and more importantly, trafficking of intrinsic or stress-induced EGFR vesicle from endocytosis has been reported as an alternative mechanism rendering cancer cell drug resistance (39, 40). Besides, the observed enrichment of class I MHC antigen processing–related pathways in TKI-resistant NSCLC cells is consistent with the reported role of endocytosis together with autophagy in MHC antigen presentation (41), which reinforces the relevance of endocytosis in TKI resistance (Fig. 2C, right panel). Given above results, the diverse annotated subcellular localization of PPIs together with the hyperactive EGFR endosomal trafficking implies that the T790M mutation may perturb EGFR signaling *via* endocytic processing.

### EGFR Location With Distinct Endosomal Protein Expression Profiles Alter EGFR Trafficking Routes in TKI-Resistant NSCLC Cells

**Fig. 3:**
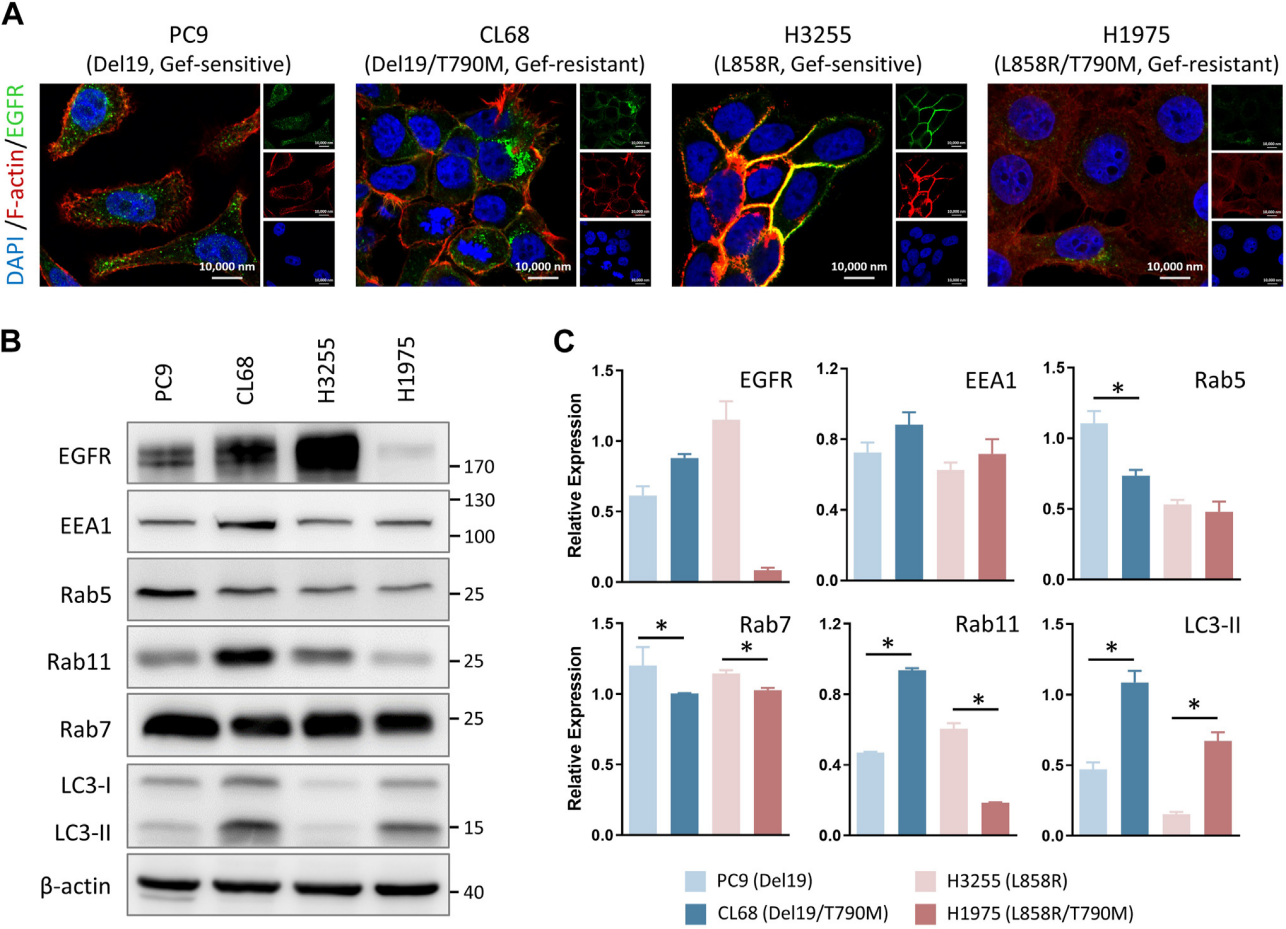
**Characterization of EGFR localization in TKI-sensitive and TKI-resistant NSCLC cells.***A*, representative confocal immunofluorescence microscopy showing the static location of endogenous EGFR (Alexa Flour 488, *green*). Cell nucleus was stained with DAPI (*blue*), and F-actin was colored with *red* using Phalloidin for cell morphology recognition. Gef, Gefitinib. *B*, the basal expression level of EGFR and protein trafficking regulators in NSCLC cells. Cell lysates were analyzed by Western blotting using antibodies against EGFR, EEA1, and Rab5 (early endosome marker), Rab11 (recycling late endosome marker), Rab7 (degradation late endosome marker), and LC3-II (autophagy marker). Β-actin was used as loading controls. *C*, the intensity of each protein band from (*B*) was quantified by image J, and the relative expression level was normalized with β-actin. Triplicate results were presented as mean ± SD. The statistical analysis was performed by two sample *t* test (∗indicated *p* value ≤0.05). Compared to TKI-sensitive cells, Rab7 and LC3-II significantly showed opposite expression trends in TKI-resistant models. EGFR, epidermal growth factor receptor; NSCLC, non-small cell lung cancer; TKI, tyrosine kinase inhibitor.

EGFR is primarily located on the plasma membrane and its intracellular trafficking *via* endocytosis-mediated internalization to various subcellular localizations is believed to alter its biological functions and subsequent signal cascades (42, 43, 44). Thus, we sought to characterize and compare the EGFR localization and potential intracellular trafficking in the TKI-resistant and TKI-sensitive cells. At the basal level, EGFR showed inherently different expression levels and distinct subcellular distributions across four NSCLC cell lines (Fig. 3*A*). Comparing the pair of PC9 (Del19) and CL68 cells (Del19/T790M), EGFR showed homogeneous distribution in the cytosol of PC9 cells, while perinuclear accumulation was observed in the CL68 cells; for L858R paired cell lines, EGFR showed strong staining primarily on plasma membrane in H3255 (L858R) cells, yet disperse cytosolic EGFR dominate in the resistant H1975 (L858R/T790M) cells. Furthermore, the expression level of a subset of endosomal proteins, which are responsible for protein intracellular trafficking, were also determined (Fig. 3*B*). Among these, EEA1, an effector regulating the fusion and docking of early endosome to autophagosome, had relatively higher but not significant expression level in CL68 and H1975 cells, implying that endosomal formation may be more active in TKI-resistant models (Fig. 3*C*). Upon early endosome formation, the next step is either fusing with autophagosome by the assistance of LC3 protein or developing into LE with the help of Rab7 (38, 45, 46). The observations of higher proportion of LC3-II to LC3-1 and the lower level of Rab7 in both TKI-resistant cells suggested the autophagy process is relatively active in NSCLC cells with EGFR T790M mutation (Fig. 3, *B* and *C*). Furthermore, the inconsistent trend of Rab11, a marker of recycling LE, implies less common relevance between two TKI-sensitive and TKI-resistant NSCLC pairs (Fig. 3, *B* and *C*). Altogether, these results indicated the alterations in EGFR subcellular localization as well as the expression levels of endosomal proteins, especially EEA1, Rab7, and LC3-II, supporting the potential role of autophagosome in modulating endocytic trafficking in TKI-resistant NSCLC cells.

### Gefitinib-Induced Autophagy Direct EGFR Degradation in TKI-Resistant NSCLC Cells

**Fig. 4:**
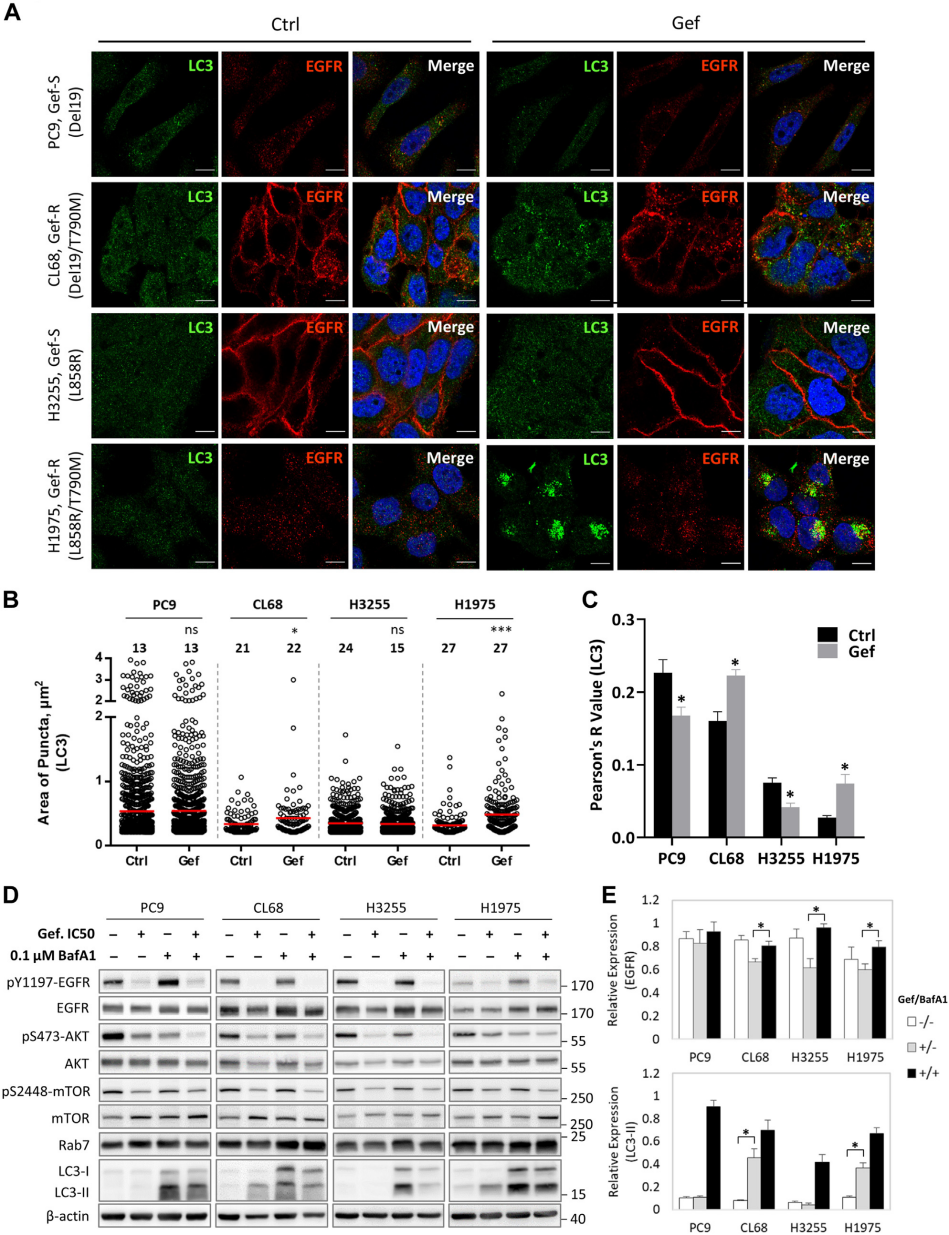
**Gefitinib induced autophagy activation in T790M-acquired TKI-resistant NSCLC cells.***A*, confocal microscopic analysis showing the immunofluorescence of LC3 (*green*), EGFR (*red*), and nucleus (*blue*). Cells were pretreated with gefitinib (Gef) at dosage of IC20 or DMSO (Ctrl) in RPMI medium supplemental with 10% FBS followed by 18 h incubation. Bar represents 10 μm. *B*, vesicle dispersion represented as puncta of LC3, and pixel area ≥0.2 μm^2^ was set as minimum threshold. *Red line* indicated the median value of LC3 puncta area. Total cell number analyzed in each NSCLC model indicated above each scatter bar. *C*, colocalization of LC3 with EGFR was assessed by Pearson’s coefficient in control cells (Ctrl) *versus* gefitinib-treated cells (Gef). *D*, validation of pathway responsible for EGFR degradation. Cells were treated by gefitinib (IC50 dosage) with (+) or without (−) BafA1 (0.1 μM) for 18 h and immunoblotted against indicated antibodies. *E*, the bar chart showed relative protein intensity EGFR and LC3-II from (*D*) measured by Image J and normalized to β-actin. The results were presented as means ± SD of individual triplicate experiments. All significance was calculated using two sample *t* test. ∗*p* value ≤0.05, ∗∗∗*p* value ≤0.001, ns = not significant. BafA1, bafilomycin A1; EGFR, epidermal growth factor receptor; NSCLC, non-small cell lung cancer; TKI, tyrosine kinase inhibitor.

To further reveal the underlying molecular basis of how the TKI treatment and resistance likely induced protein trafficking, we administered gefitinib to all these four NSCLC cells to create the iatrogenic stress for dissecting the roles of endocytic trafficking in TKI resistance. As expected, gefitinib efficiently suppressed EGFR kinase activity as seen in the decreased phosphorylated level of activation site, Tyr1197 in EGFR, accompanying by the lower EGFR expression in a time-dependent manner in fours NSCLC models (supplemental Fig. S3*A*). Significantly, the LC3-II expression, a marker for autophagosome activation, only increased in TKI-resistant cells (CL68 and H1975) in a time- and dose-dependent manner, though Rab7 and LC3-I remained constant levels (supplemental Fig. S3, *A* and *B*). LC3-II is generated through the ubiquitination-like enzymatic cleavage of the phosphatidylethanolamine-conjugated LC3-I on the surface of nascent autophagosome (47). The augment of LC3-II revealed that the administration of gefitinib induced autophagic flux and increased autophagic activity in NSCLC cells harboring EGFR T790M mutation (supplemental Fig. S3*C*). In line with this, it intrigued us to further explore the relationship between EGFR and LC3 under gefitinib treatment. Upon 18-h gefitinib treatment, the LC3 puncta sizes were significantly increased and accumulated perinuclearly in TKI-resistant cells (CL68 and H1975) but not in TKI-sensitive cells (PC9 and H3255) (Fig. 4, *A* and *B*, green color). Moreover, in both TKI-resistant models, LC3 and EGFR were highly colocalized (Pearson’s R value show data), indicating that the decreased EGFR expression and activity may be the consequence of the autophagosome-mediated EGFR degradation (Fig. 4*C* and supplemental Fig. S3). Meanwhile, proteasome inhibitor MG-132 and caspase inhibitor Z-VAD-FMK were unable to efficiently block the gefitinib-induced EGFR degradation, excluding the potential involvement of these two pathways and thus emphasizing the importance of autophagy in EGFR degradation (supplemental Fig. S3*D*). However, it is noted that MG-132 synergistically prompted the autophagy activation in four NSCLC cells, evidenced by the increased LC3-II level which leads to even more remarkable EGFR decrease through autophagic degradation (supplemental Fig. S3*D*).

To further verify the role of autophagy in response to TKI stimulation, the NSCLC cells were cotreated with gefitinib and autophagosome-lysosome fusion inhibitor BafA1. Consistent with previous studies, BafA1 sufficiently blocked the autolysosome formation which resulted in increased level of LC3-II, due to the accumulation of autophagosomes, yet did not affect the EGFR autophosphorylation (pY1197) status and cell viability (Fig. 4*E* and supplemental Fig. S4, *A* and *B*). Furthermore, the autophagy-mediated EGFR degradation was largely relieved when TKI-resistant NSCLC cells were cotreated with BafA1 and gefitinib (Fig. 4, *D* and *E*). Further cotreating gefitinib and 3-MA, a dual inhibitor of PI3K and autophagy, significantly inhibited the cell growth of TKI-resistant NSCLC cells (CL68 and H1975, supplemental Fig. S5*A*) and decreased the LC3-II protein level, though the EGFR degradation was not rescued (supplemental Fig. S5, *B* and *C*). This could be explained by the antiproliferation effect of 3-MA through the inhibition of the PI3K–FOXO3a pathway (48). These results indicated that autophagosome-mediated protein EGFR degradation may provide the TKI-resistant NSCLC cells ability to escape TKI inhibition. Among the heterogeneous interactors from different EGFR subtypes, excluding housekeeping EGFR interactors, the proteins participated in up-, middle-, and down-stream endosomal trafficking, including NEDD4L, VTA1, and p62, were selected to verify their interaction with EGFR. Consistent with our AP-MS result, p62 and VTA1 proteins showed a weak or transient interaction with EGFR, while NEDD4L, a ubiquitin-protein ligase which regulates membrane protein internalization and turnover through ubiquitination (49), presented a stronger interaction with EGFR (supplemental Fig. S5*D*, I). Furthermore, the reverse Co-IP assay using NEDD4L again affirmed the interaction between EGFR and NEDD4L (supplemental Fig. S5*D*, II), which was first identified by our EGFR interactome analysis.

### Gefitinib Induced Rab7-Mediated EGFR Transportation in TKI-Sensitive NSCLC Cells

**Fig. 5:**
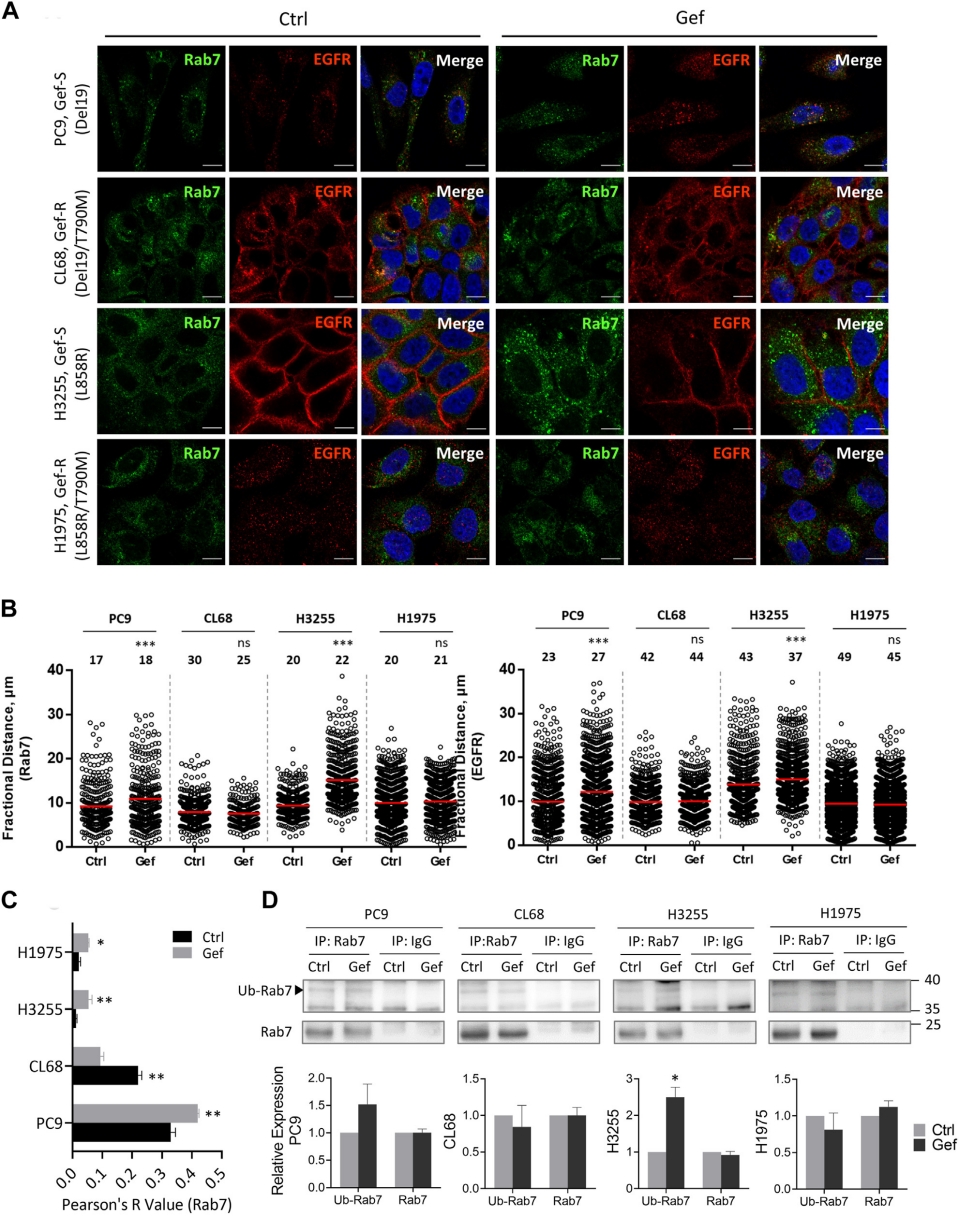
**Effect of gefitinib on Rab7-mediated endosomal transportation in NSCLC cells.***A*, confocal microscopic analysis representing immunofluorescence of Rab7 (*green*), EGFR (*red*), and nucleus (*blue*). Cells were treated with gefitinib (Gef) at the dosage of IC20 or DMSO (Ctrl) in RPMI medium supplemental with 10% FBS for 18 h. Bar represents 10 μm. *B*, vesicle dispersion presented as a fractional distance of Rab7 (*top*) and EGFR (*bottom*) dot along a straight line to the nuclear center. The dot used for fractional distance calculation was set as pixel area ≥0.2 μm^2^, and the *red line* indicated the median value. Total cell number analyzed in each NSCLC cell model was indicated on the *top* of the scatter bar. *C*, colocalization of Rab7 and EGFR was quantified by the Pearson’s coefficient. *D*, Rab7 proteins were immunoprecipitated from the cells treated with gefitinib at IC50 dosage or DMSO using anti-Rab7 and rabbit IgG antibodies as control. The precipitants were analyzed by Western blotting using anti-ubiquitin and anti-Rab7 antibodies. Relative intensity was measured by Image J and presented with mean ± SD (n = 3). All significance was calculated using two sample *t* test. ∗*p* value ≤0.05, ∗∗*p* value ≤0.01, ∗∗∗*p* value ≤0.001, ns = not significant. EGFR, epidermal growth factor receptor; IgG, immunoglobulin G; NSCLC, non-small cell lung cancer.

The hyperactivation LC3 implied the vigorous autophagosome formation in TKI-resistant NSCLC cells (Fig. 4); however, the fate of LE transportation is subsequently decided by the ubiquitination status of Rab7 (50, 51, 52). In this study, the higher Rab7 expression in PC9 and H3255 cells suggested that the gefitinib-induced EGFR reduction is possibly mediated by endocytic trafficking of the LE (Fig. 3, *B* and *C* and supplemental Fig. S3, *A* and *B*). Upon 18-h gefitinib treatment, Rab7 notably aggregated in cytosol in TKI-sensitive NSCLC cells but it showed consistently perinuclear localizations in TKI-resistant NSCLC cells (Fig. 5*A*). The localization movement was quantified by the fractional distance, which is measured from the center of the nucleus to either the Rab7 or EGFR dots in a defined cellular region. Clearly, the gefitinib treatment redistributed both EGFR and Rab7 location closer to plasma membrane and preferentially colocalized in TKI-sensitive PC9 and H3255 cells (Fig. 5*B*, top). In contrast, the localization of EGFR and Rab7 was not affected in TKI-resistant NSCLC cells upon gefitinib treatment (Fig. 5, *B* and *C*). Furthermore, the ubiquitination extent on Rab7 was slightly higher (∼38 kDa molecular weight) in PC9 and H3255 cells after gefitinib treatment, indicating that EGFR-carried LE tended to move toward plasma membrane which may confer TKIs more chance to suppress EGFR activity (Fig. 5*D*, PC9 and H3255). However, the ubiquitination level remained consistent in TKI-resistant models (Fig. 5*D*, CL68 and H1975). Accordingly, our results suggested that the recycling endosome route was more prominent in response to the gefitinib treatment in the NSCLC cells harboring primary EGFR mutation, which will promote the TKI therapeutic efficacy and cell death.

## Discussion

The dysregulation of EGFR signaling is a common cause of tumorigenesis as it leads to aberrant cancer cell survival, invasion, and metastasis. High prevalence of constantly activating EGFR mutations, such as Del19 and L858R, is observed in more than 50% East Asia NSCLC patients, making the TKIs treatment efficient and successful at the beginning. However, patients eventually develop TKI resistance and tumor relapses due to the occurrence of secondary EGFR mutation, T790M (53, 54). It has been well documented that NSCLC patients possessing different EGFR activation mutations, Del19 or L858R, show varied clinical characteristics, TKI responses, as well as the treatment outcome (55). Furthermore, the NSCLC patients with EGFR L858R usually show greater malignancy and higher metastasis rates compared to those with EGFR Del19 (56). The co-occurrence frequency of these two activating EGFR mutations in NSCLC patients is extremely low; it is challenging and not relevant to obtain two mutation types from the same parental cell line. Therefore, we used the pairs of patient-derived NSCLC cell lines with the same primary EGFR mutation to dissect the T790M-specific pathways, which may better reflect the heterogeneity of EGFR resistance mutations and patient phenotypes. We utilized the pairwise comparison of patient-derived NSCLC cell lines as study models to gain a glimpse into how T790M perturbed EGFR downstream signals underlying TKI resistance. We adopt the AP-MS approach to comprehensively identify the EGFR interactome dynamics using two pairs of NSCLC cell lines harboring the same EGFR primary mutations, Del19 or L858R, with or without the T790M mutation (21, 57). A total of 187 highly confident EGFR interactors were identified, and the following network and pathway analysis pointed out the apparent discrepancies on endocytic trafficking between TKI-sensitive and TKI-resistant NSCLC cells.

**Fig. 6:**
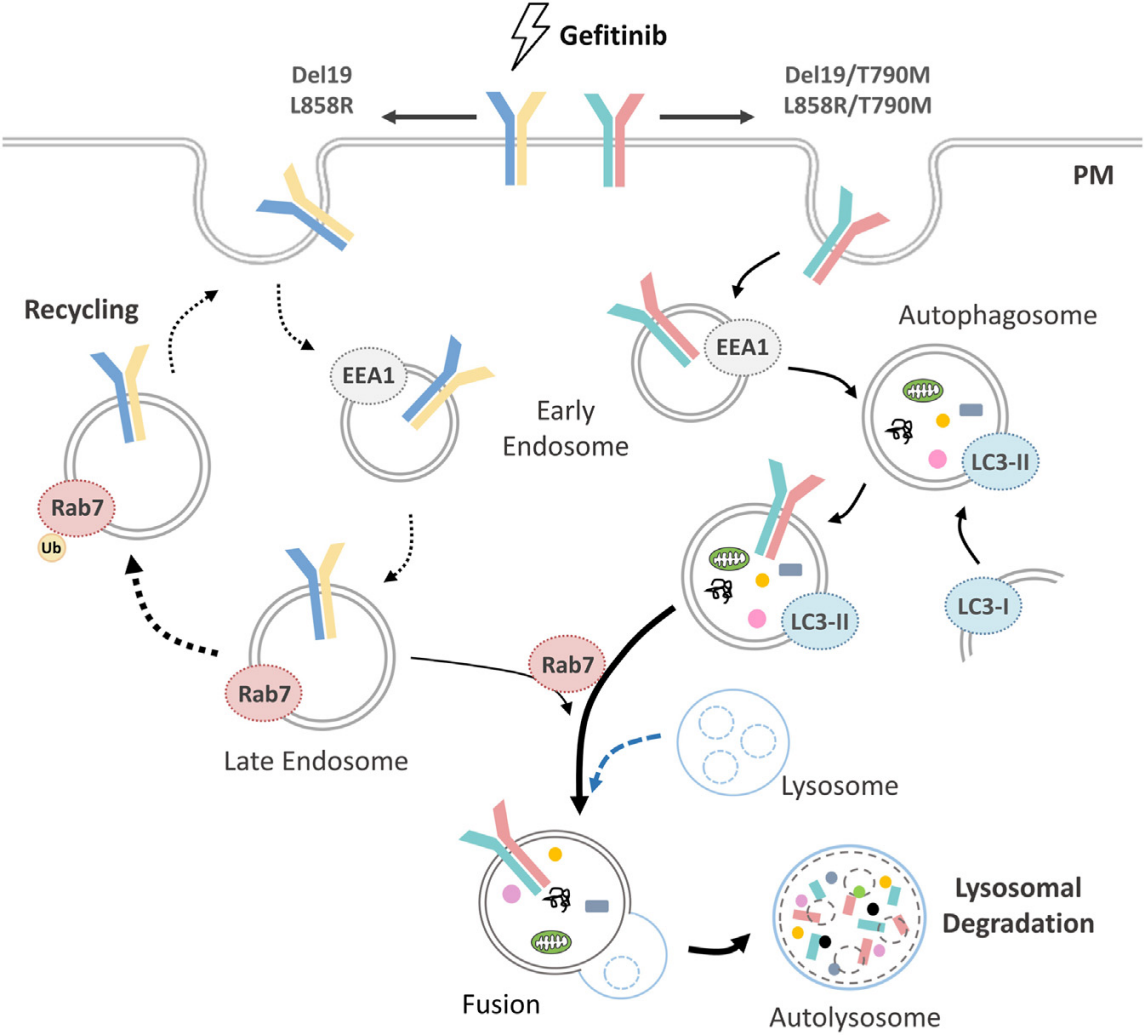
**The model depicting gefitinib-induced cellular stress triggers EGFR trafficking and signaling through distinct mechanisms between TKI-sensitive and TKI-resistant NSCLC models.** In TKI-resistant lung cancer cells, the treatment of gefitinib triggered the autophagy-mediated EGFR degradation to defense gefitinib targeting, resulting in T790M mutation–dependent drug resistance. Gefitinib induced endosomal recycling pathway in TKI-sensitive cells which has EGFR Del19 or L858R mutation. EGFR, epidermal growth factor receptor; NSCLC, non-small cell lung cancer; TKI, tyrosine kinase inhibitor.

EGFR-mediated endocytic trafficking is reported as a crucial mechanism for tumorigenesis as well as a bypass drug-resistant mechanism under iatrogenic cellular stress, such as TKI treatment (45, 58, 59). Since EGFR internalization/translocation is known to be perturbed by the presence of TKIs (60), we therefore investigated the roles of identified PPIs in EGFR trafficking mechanisms upon gefitinib treatment in NSCLC cells. We found that autophagy-mediated lysosomal EGFR degradation prompted cell survival upon gefitinib treatment in both TKI-resistant models as cotreating gefitinib with the 3-MA could facilitate the gefitinib cytotoxicity which is not observed in TKI-sensitive models (Fig. 4 and supplemental Fig. S4). Although the nonspecificity and off-target effect of autophagy inhibitors (61) may confound the elucidation of autophagy in TKI-resistant mechanism, we have carefully interrogated the relevance by employing multiple autophagy modulators to confirm their modes of action as potential therapeutic targets (Fig. 4 and supplemental Figs. S3 and S5). In contrast, gefitinib administration rerouted their EGFR location to cell periphery through endosome recycling as Rab7 ubiquitination level was upregulated in TKI-sensitive NSCLC models (Fig. 5). These results suggested that the difference of EGFR interactome components at basal level are crucial for deciding the EGFR trafficking routes to respond or extenuate the TKI insult (Fig. 6). Notably, we found the ubiquitination system, such as NEDD4L, ITCH, WWP1/2, and SMURF2, was obviously enriched in EGFR interactome networks which is known to involve in many aspects of protein internalization and trafficking (supplemental Fig. S2) (62, 63, 64). According to our AP-MS analysis and IP experiments, NEDD4L was proved as a novel EGFR interactor, which is a ubiquitin E3 ligase involved in switching autophagic response through modulating the ubiquitination level of PIK3CA and UBK1 (65, 66). Furthermore, NEDD4L has been proved to act as a tumor suppressor through regulating EGFR, TGFβ, and Wnt signaling pathways (67), and the lower NEDD4L level showed poor prognosis in a variety of cancers clinically (68, 69). Therefore, understanding the interplays between ubiquitin system and protein trafficking may offer a novel avenue for cancer management.

The EGFR activation governs versatile cellular progresses and thus its subcellular trafficking *via* endocytic vesicle is highly related to distinct biological functions. Dysregulation of EGFR trafficking leads to malignancy and varied treatment effectiveness. For example, the nuclear EGFR is involved in transcriptional regulation, cell proliferation, DNA repair, and chemo-resistance (43, 70), while the EGFR located in mitochondria plays a vital role in modulating mitochondrial functions through phosphorylation of COXII, which is correlated with cell survival and metastasis (44, 71). Furthermore, patients with membrane-localized mutant EGFRs have significantly longer progression-free survival (PFS) than those with cytoplasmic-localized EGFRs due to the higher TKI therapeutic efficacy (72, 73). Accordingly, even patients with identical EGFR mutation subtypes would have varied sensitivity of therapies because of the different EGFR subcellular localizations. To date, it is known that EGFR endocytosis and cytoprotective autophagy triggered by TKI treatment serve as bypass drug-resistant mechanisms in cancer cells; however, the underlying mechanism remains a mystery (59, 74, 75, 76). We proved that the acquired T790M EGFR mutation significantly rewired EGFR trafficking fates *via* switching EGFR interactors in response to TKI treatment, that is, TKI induced autophagy-mediated EGFR degradation in both TKI-resistant models but prompted EGFR recycling back to plasma membrane in NSCLC cells only possessing primary EGFR mutations (Fig. 6). Therefore, targeting autophagy by clinically approved inhibitors, such as hydroxychloroquine (77), or the more specific strategies for autophagy modulation, such as nanoparticle-orientated autophagy inhibition (78), may enhance efficacy of TKI therapy and tackle the tumor relapse in TKI-resistant NSCLC patients.

As acquired EGFR secondary mutation is inevitable in patients administered with TKI treatments, new therapeutic strategies are required to improve patients’ benefit and conquer resistance. It is noteworthy that MS-based interactome across different mutant EGFRs shed insights on the molecular basis and PPIs analysis, highlighting the importance of switching key EGFR interactors for EGFR endocytic trafficking by acquired T790M EGFR mutation. In particular, regulating the activity of autophagy was evaluated as a crucial TKI-resistant mechanism; this provided opportunities to develop novel therapeutic strategies or combined treatment for lung cancer patients.

## Data Availability

All raw data that support the findings of this study have been deposited in jPOST repository (https://repository.jpostdb.org) with the accession number as JPST001609 for jPOST and PXD034228 for ProteomeXchange.

## Supplemental Data

This article contains supplemental data.
