# Supplementary material for: EGFR-T790M Mutation–Derived Interactome Rerouted EGFR Translocation Contributing to Gefitinib Resistance in Non-Small Cell Lung Cancer
Source: Mol Cell Proteomics. 2023 Jul 24;22(9):100624. doi: 10.1016/j.mcpro.2023.100624 (PMC10545940; doi:10.1016/j.mcpro.2023.100624)
Supplement: Supplemental Figures S1–S5 [file mmc1.pptx]

## Slide 1
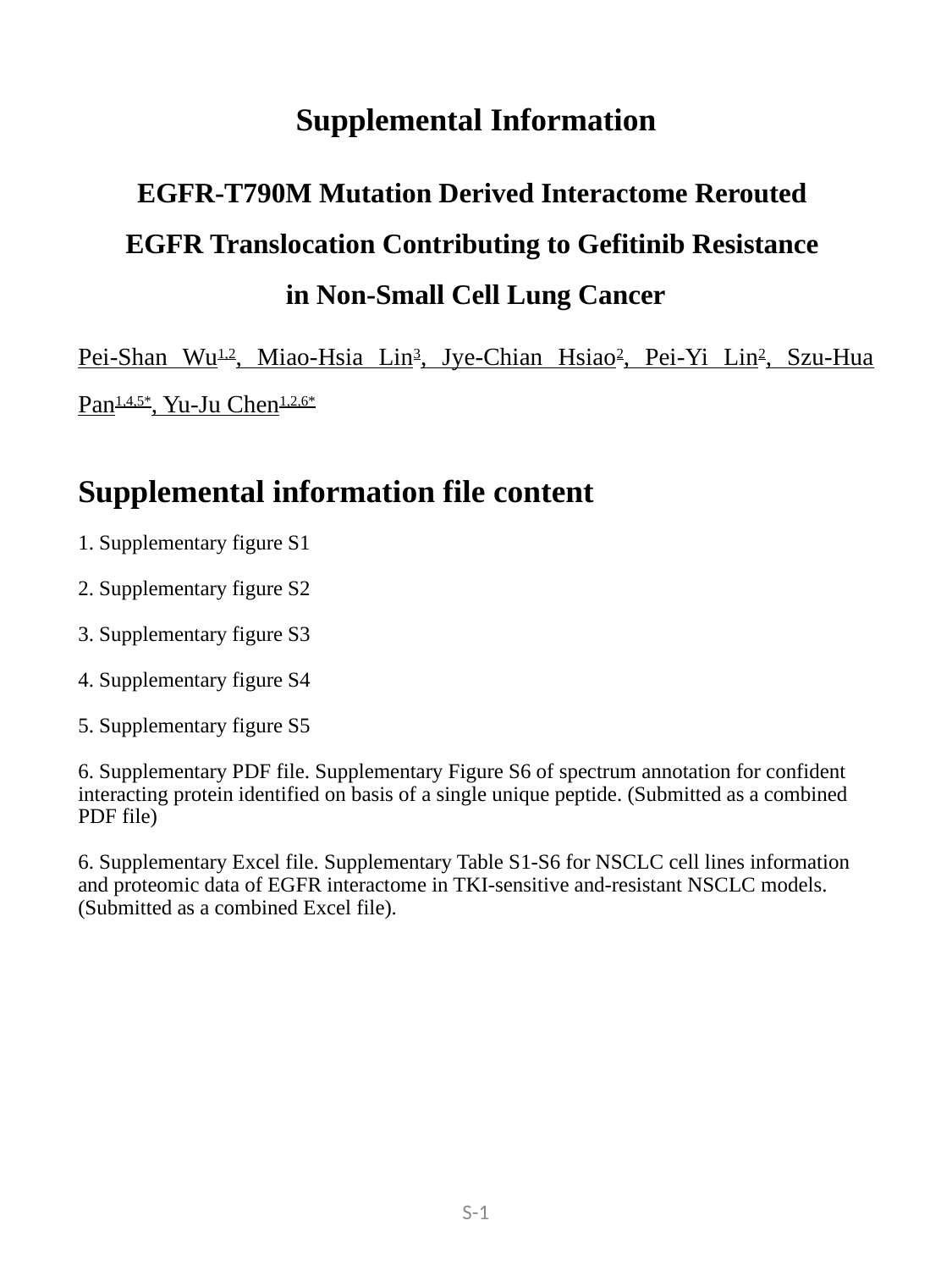

Supplemental Information
EGFR-T790M Mutation Derived Interactome Rerouted
EGFR Translocation Contributing to Gefitinib Resistance
in Non-Small Cell Lung Cancer
Pei-Shan Wu1,2, Miao-Hsia Lin3, Jye-Chian Hsiao2, Pei-Yi Lin2, Szu-Hua Pan1,4,5*, Yu-Ju Chen1,2,6*
Supplemental information file content
1. Supplementary figure S1
2. Supplementary figure S2
3. Supplementary figure S3
4. Supplementary figure S4
5. Supplementary figure S5
6. Supplementary PDF file. Supplementary Figure S6 of spectrum annotation for confident interacting protein identified on basis of a single unique peptide. (Submitted as a combined PDF file)
6. Supplementary Excel file. Supplementary Table S1-S6 for NSCLC cell lines information and proteomic data of EGFR interactome in TKI-sensitive and-resistant NSCLC models. (Submitted as a combined Excel file).
S-1

## Slide 2
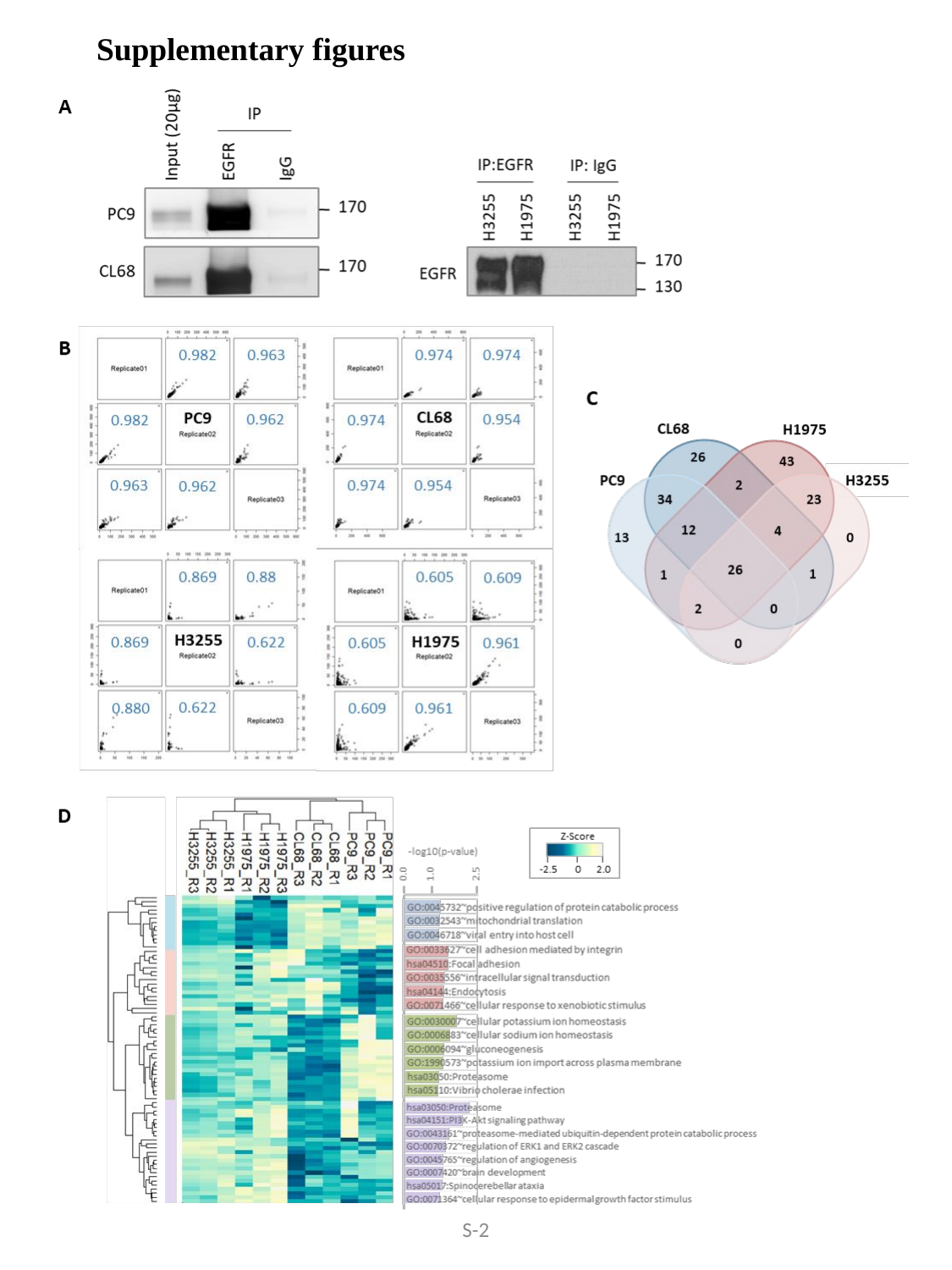

Supplementary figures
S-2

## Slide 3
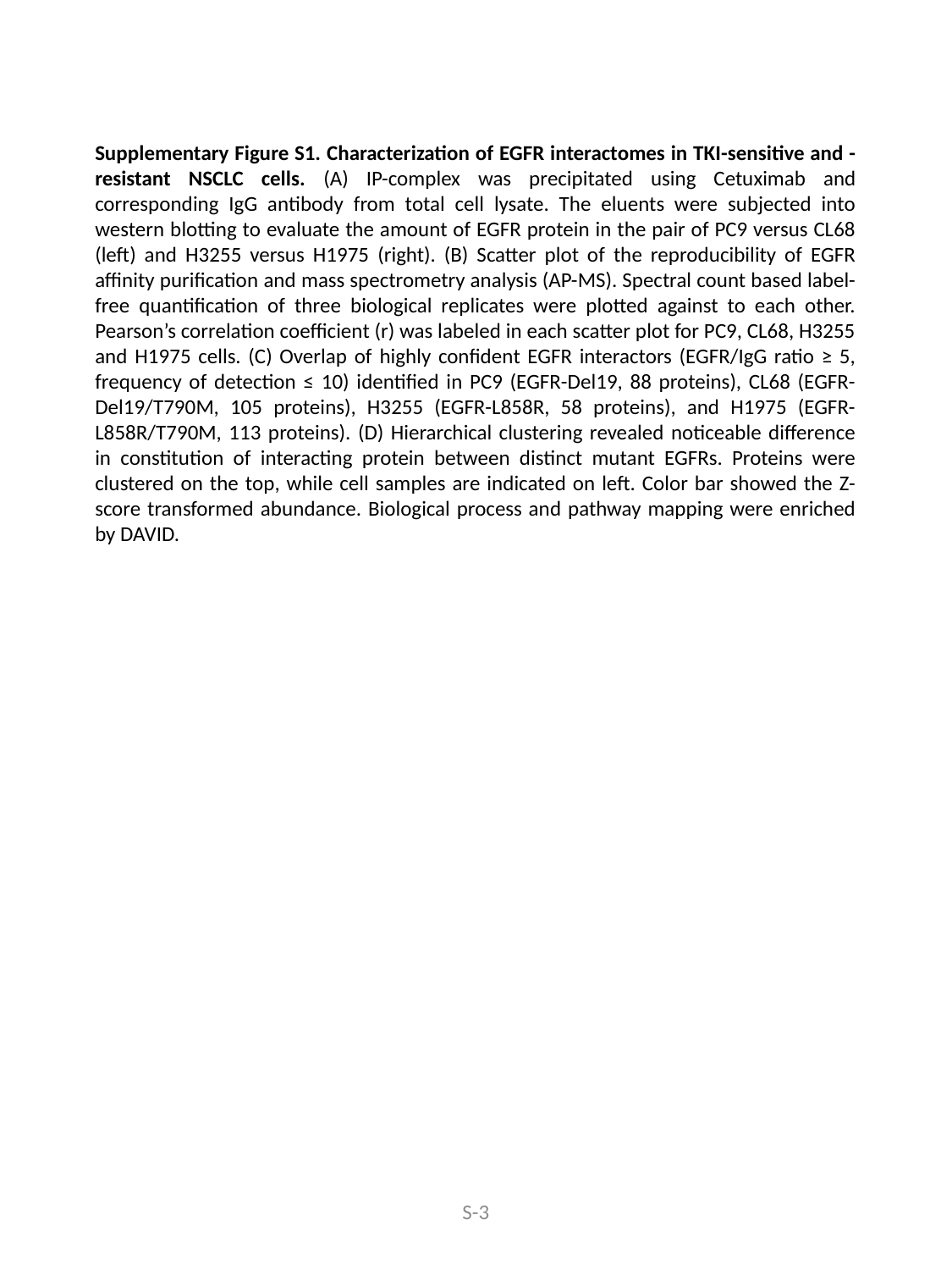

Supplementary Figure S1. Characterization of EGFR interactomes in TKI-sensitive and -resistant NSCLC cells. (A) IP-complex was precipitated using Cetuximab and corresponding IgG antibody from total cell lysate. The eluents were subjected into western blotting to evaluate the amount of EGFR protein in the pair of PC9 versus CL68 (left) and H3255 versus H1975 (right). (B) Scatter plot of the reproducibility of EGFR affinity purification and mass spectrometry analysis (AP-MS). Spectral count based label-free quantification of three biological replicates were plotted against to each other. Pearson’s correlation coefficient (r) was labeled in each scatter plot for PC9, CL68, H3255 and H1975 cells. (C) Overlap of highly confident EGFR interactors (EGFR/IgG ratio ≥ 5, frequency of detection ≤ 10) identified in PC9 (EGFR-Del19, 88 proteins), CL68 (EGFR-Del19/T790M, 105 proteins), H3255 (EGFR-L858R, 58 proteins), and H1975 (EGFR-L858R/T790M, 113 proteins). (D) Hierarchical clustering revealed noticeable difference in constitution of interacting protein between distinct mutant EGFRs. Proteins were clustered on the top, while cell samples are indicated on left. Color bar showed the Z-score transformed abundance. Biological process and pathway mapping were enriched by DAVID.
S-3

## Slide 4
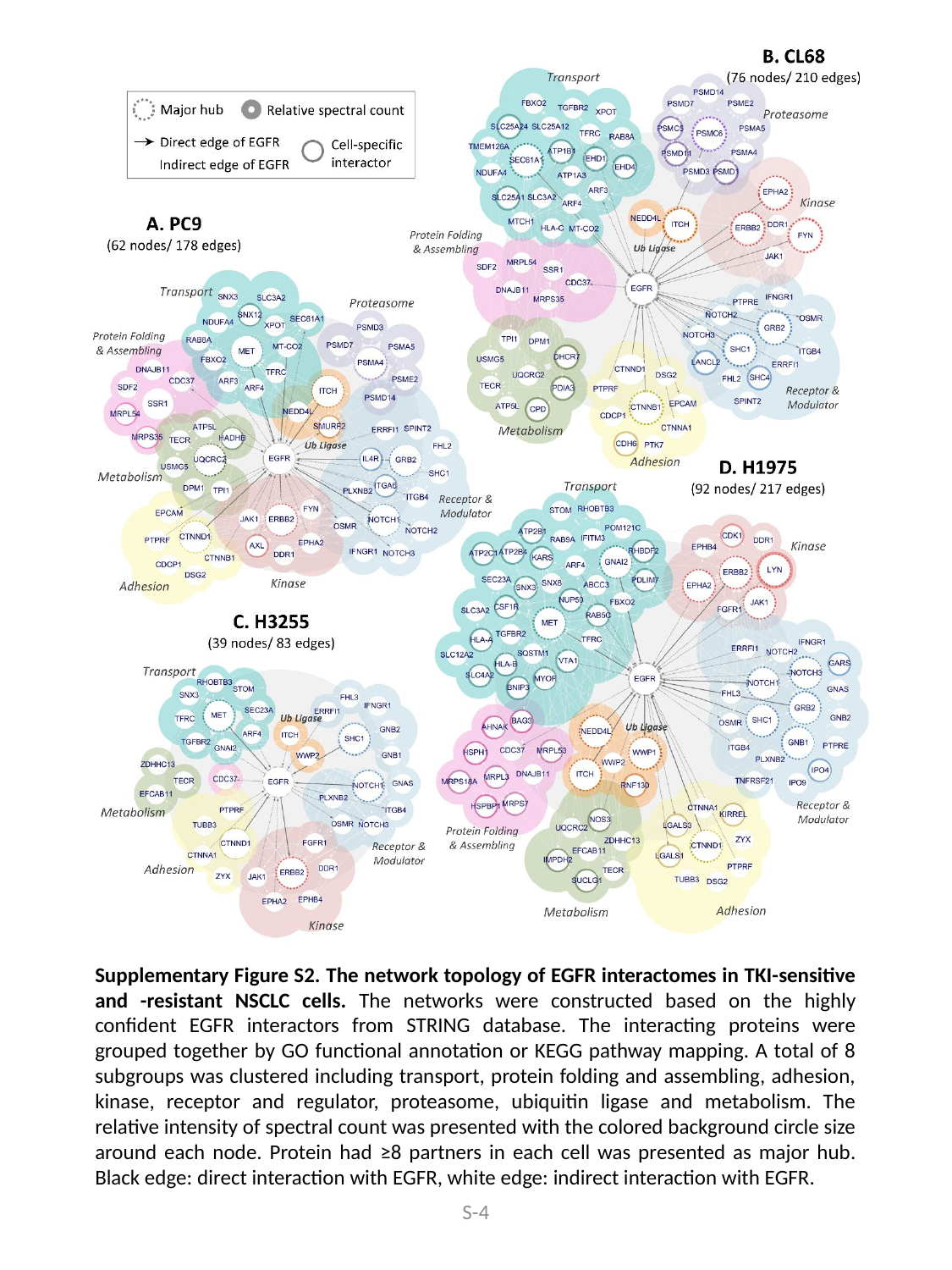

Supplementary Figure S2. The network topology of EGFR interactomes in TKI-sensitive and -resistant NSCLC cells. The networks were constructed based on the highly confident EGFR interactors from STRING database. The interacting proteins were grouped together by GO functional annotation or KEGG pathway mapping. A total of 8 subgroups was clustered including transport, protein folding and assembling, adhesion, kinase, receptor and regulator, proteasome, ubiquitin ligase and metabolism. The relative intensity of spectral count was presented with the colored background circle size around each node. Protein had ≥8 partners in each cell was presented as major hub. Black edge: direct interaction with EGFR, white edge: indirect interaction with EGFR.
S-4

## Slide 5
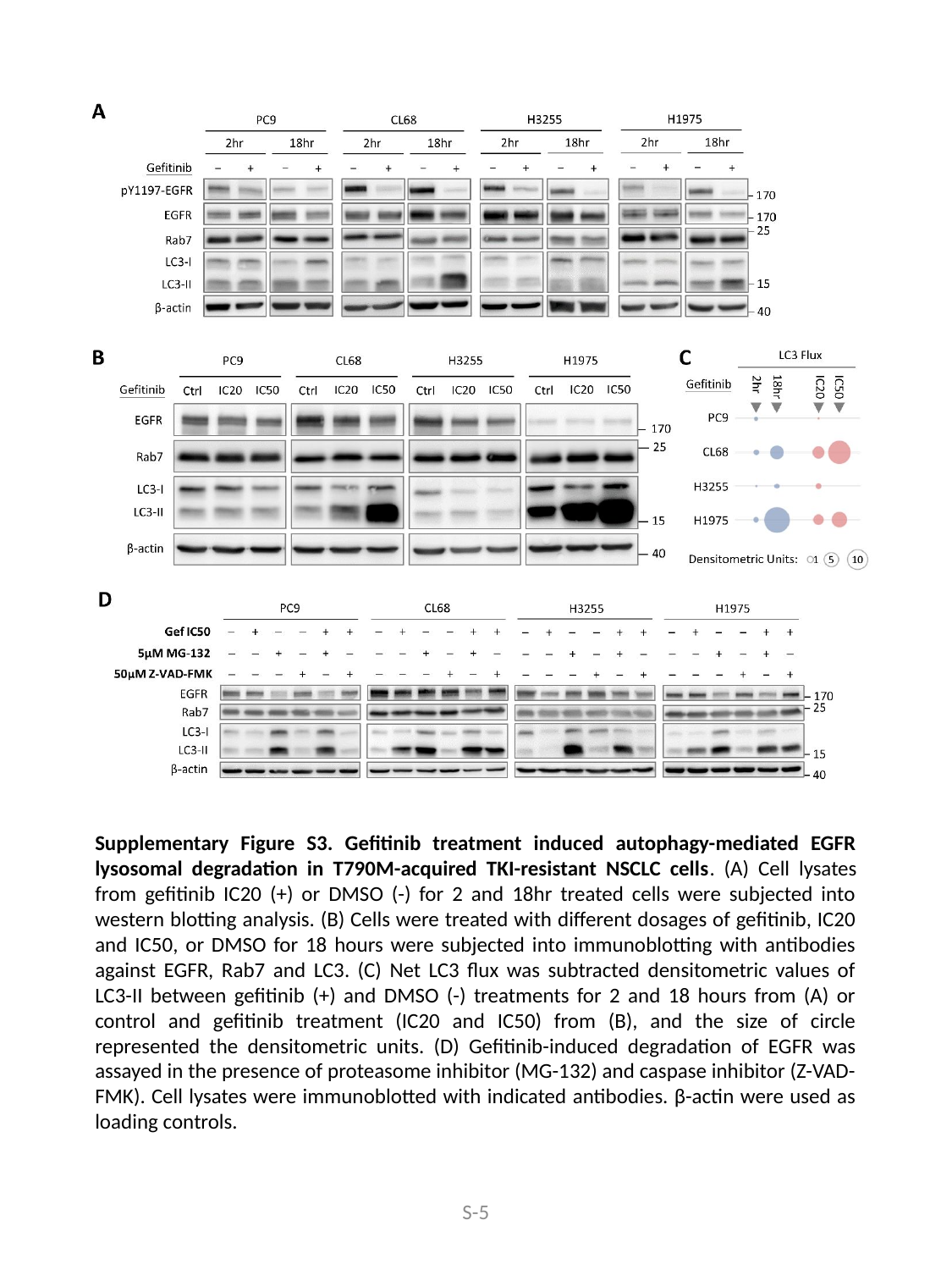

Supplementary Figure S3. Gefitinib treatment induced autophagy-mediated EGFR lysosomal degradation in T790M-acquired TKI-resistant NSCLC cells. (A) Cell lysates from gefitinib IC20 (+) or DMSO (-) for 2 and 18hr treated cells were subjected into western blotting analysis. (B) Cells were treated with different dosages of gefitinib, IC20 and IC50, or DMSO for 18 hours were subjected into immunoblotting with antibodies against EGFR, Rab7 and LC3. (C) Net LC3 flux was subtracted densitometric values of LC3-II between gefitinib (+) and DMSO (-) treatments for 2 and 18 hours from (A) or control and gefitinib treatment (IC20 and IC50) from (B), and the size of circle represented the densitometric units. (D) Gefitinib-induced degradation of EGFR was assayed in the presence of proteasome inhibitor (MG-132) and caspase inhibitor (Z-VAD-FMK). Cell lysates were immunoblotted with indicated antibodies. β-actin were used as loading controls.
S-5

## Slide 6
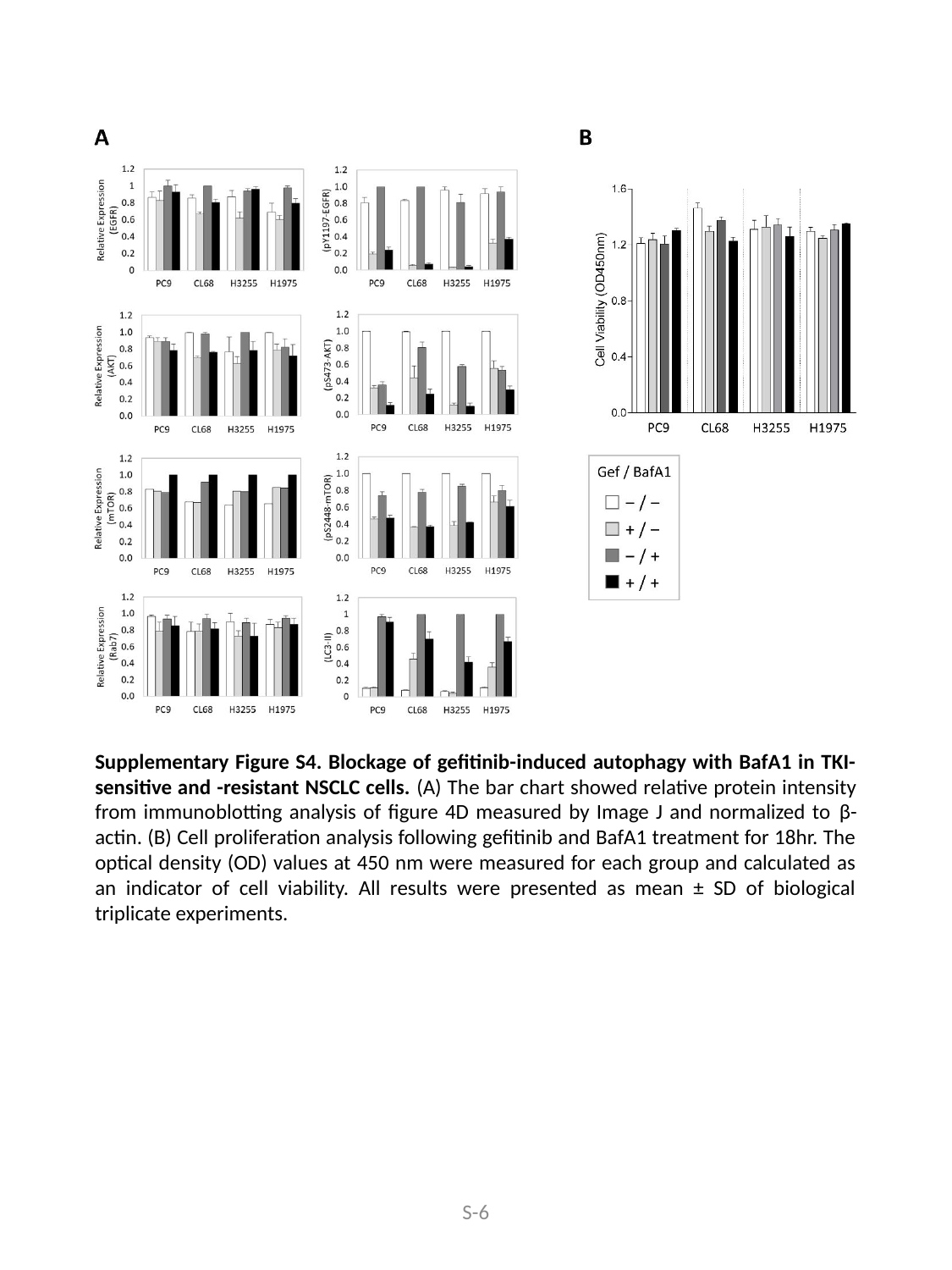

Supplementary Figure S4. Blockage of gefitinib-induced autophagy with BafA1 in TKI-sensitive and -resistant NSCLC cells. (A) The bar chart showed relative protein intensity from immunoblotting analysis of figure 4D measured by Image J and normalized to β-actin. (B) Cell proliferation analysis following gefitinib and BafA1 treatment for 18hr. The optical density (OD) values at 450 nm were measured for each group and calculated as an indicator of cell viability. All results were presented as mean ± SD of biological triplicate experiments.
S-6

## Slide 7
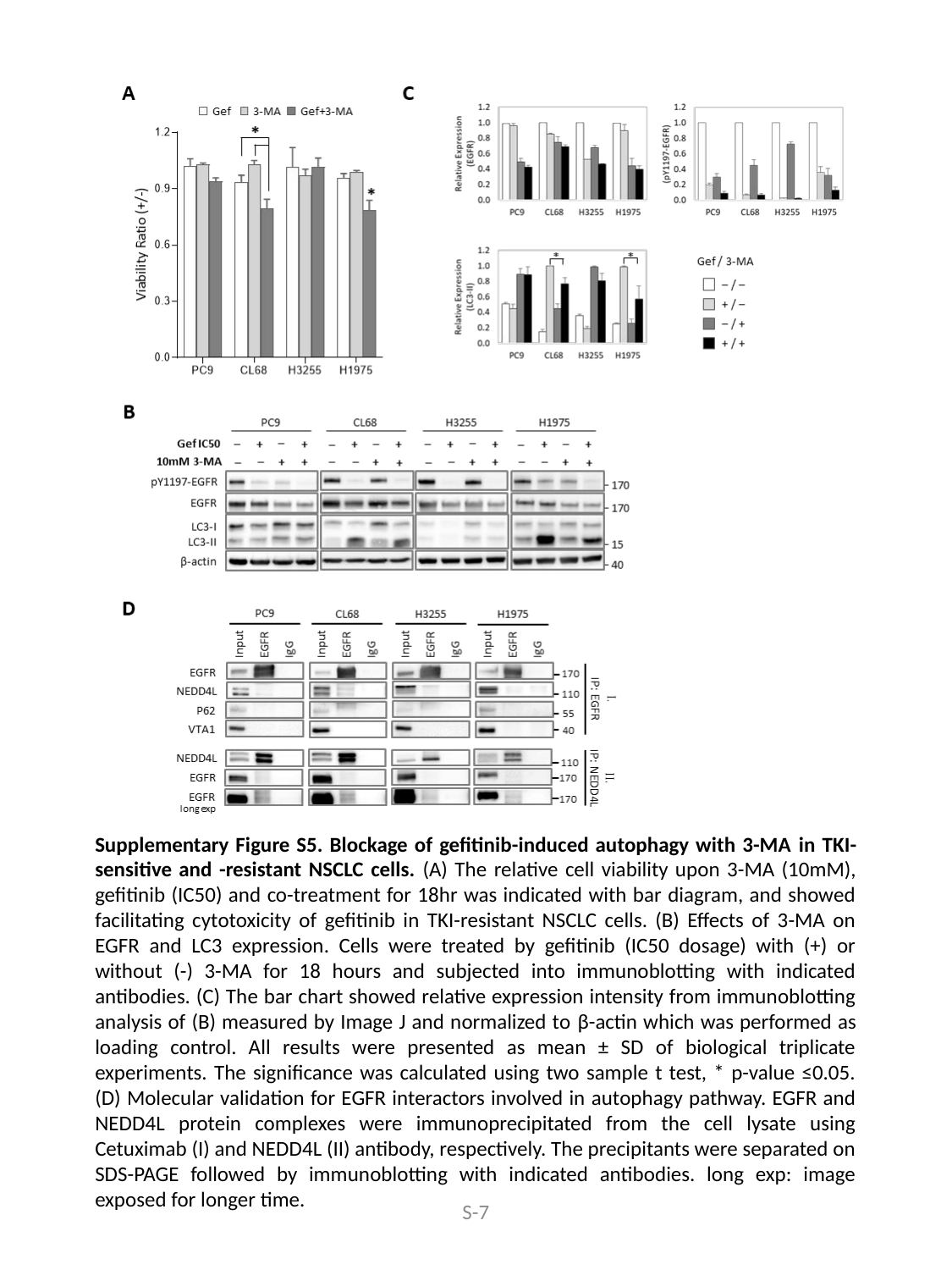

Supplementary Figure S5. Blockage of gefitinib-induced autophagy with 3-MA in TKI-sensitive and -resistant NSCLC cells. (A) The relative cell viability upon 3-MA (10mM), gefitinib (IC50) and co-treatment for 18hr was indicated with bar diagram, and showed facilitating cytotoxicity of gefitinib in TKI-resistant NSCLC cells. (B) Effects of 3-MA on EGFR and LC3 expression. Cells were treated by gefitinib (IC50 dosage) with (+) or without (-) 3-MA for 18 hours and subjected into immunoblotting with indicated antibodies. (C) The bar chart showed relative expression intensity from immunoblotting analysis of (B) measured by Image J and normalized to β-actin which was performed as loading control. All results were presented as mean ± SD of biological triplicate experiments. The significance was calculated using two sample t test, * p-value ≤0.05. (D) Molecular validation for EGFR interactors involved in autophagy pathway. EGFR and NEDD4L protein complexes were immunoprecipitated from the cell lysate using Cetuximab (I) and NEDD4L (II) antibody, respectively. The precipitants were separated on SDS-PAGE followed by immunoblotting with indicated antibodies. long exp: image exposed for longer time.
S-7
