# Supplementary material for: EGFR-T790M Mutation–Derived Interactome Rerouted EGFR Translocation Contributing to Gefitinib Resistance in Non-Small Cell Lung Cancer
Source: Mol Cell Proteomics. 2023 Jul 24;22(9):100624. doi: 10.1016/j.mcpro.2023.100624 (PMC10545940; doi:10.1016/j.mcpro.2023.100624)
Supplement: Supplemental Tables S1–S6 [file mmc3.pptx]

## Slide 1
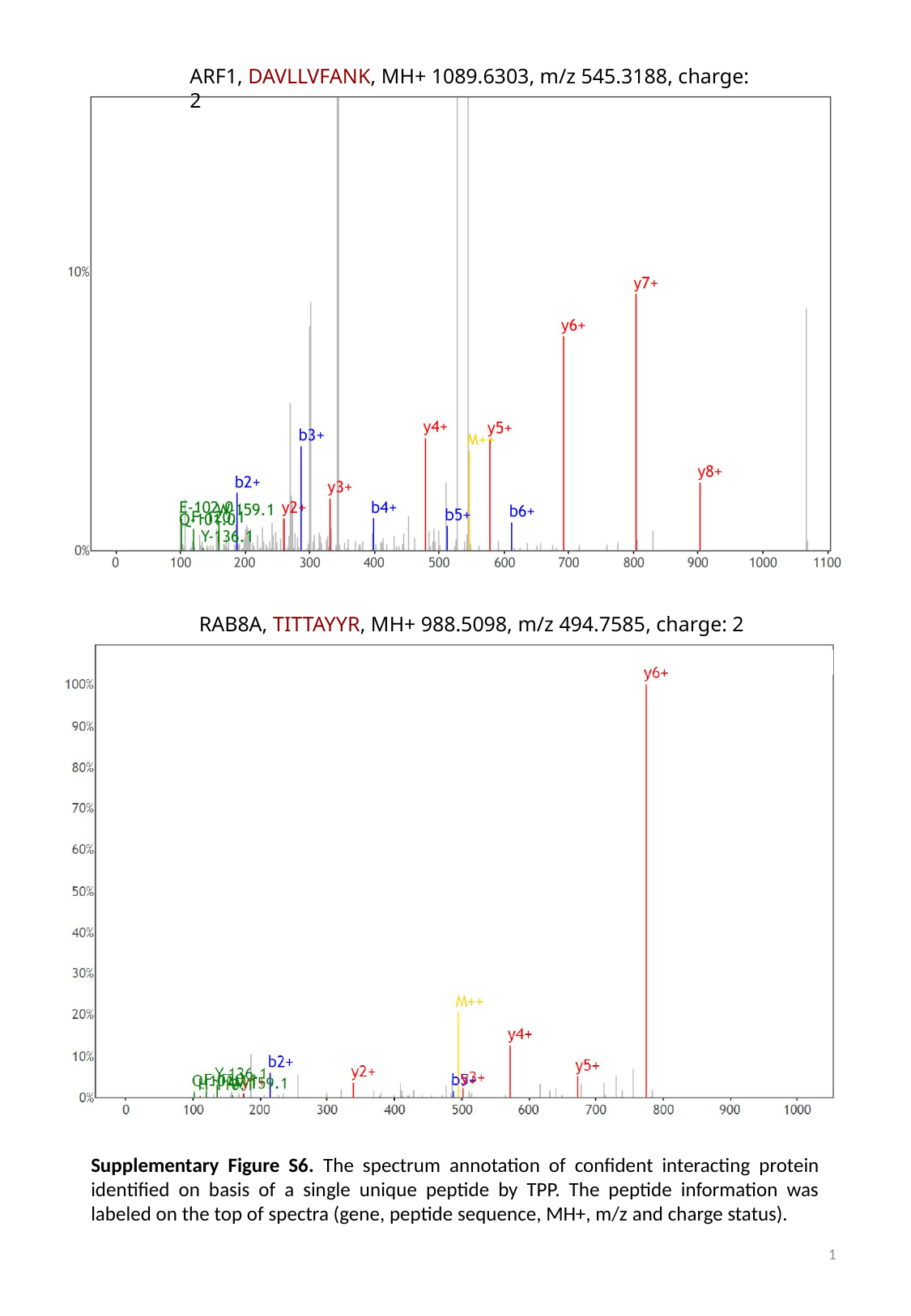

ARF1, DAVLLVFANK, MH+ 1089.6303, m/z 545.3188, charge: 2
RAB8A, TITTAYYR, MH+ 988.5098, m/z 494.7585, charge: 2
Supplementary Figure S6. The spectrum annotation of confident interacting protein identified on basis of a single unique peptide by TPP. The peptide information was labeled on the top of spectra (gene, peptide sequence, MH+, m/z and charge status).
1

## Slide 2
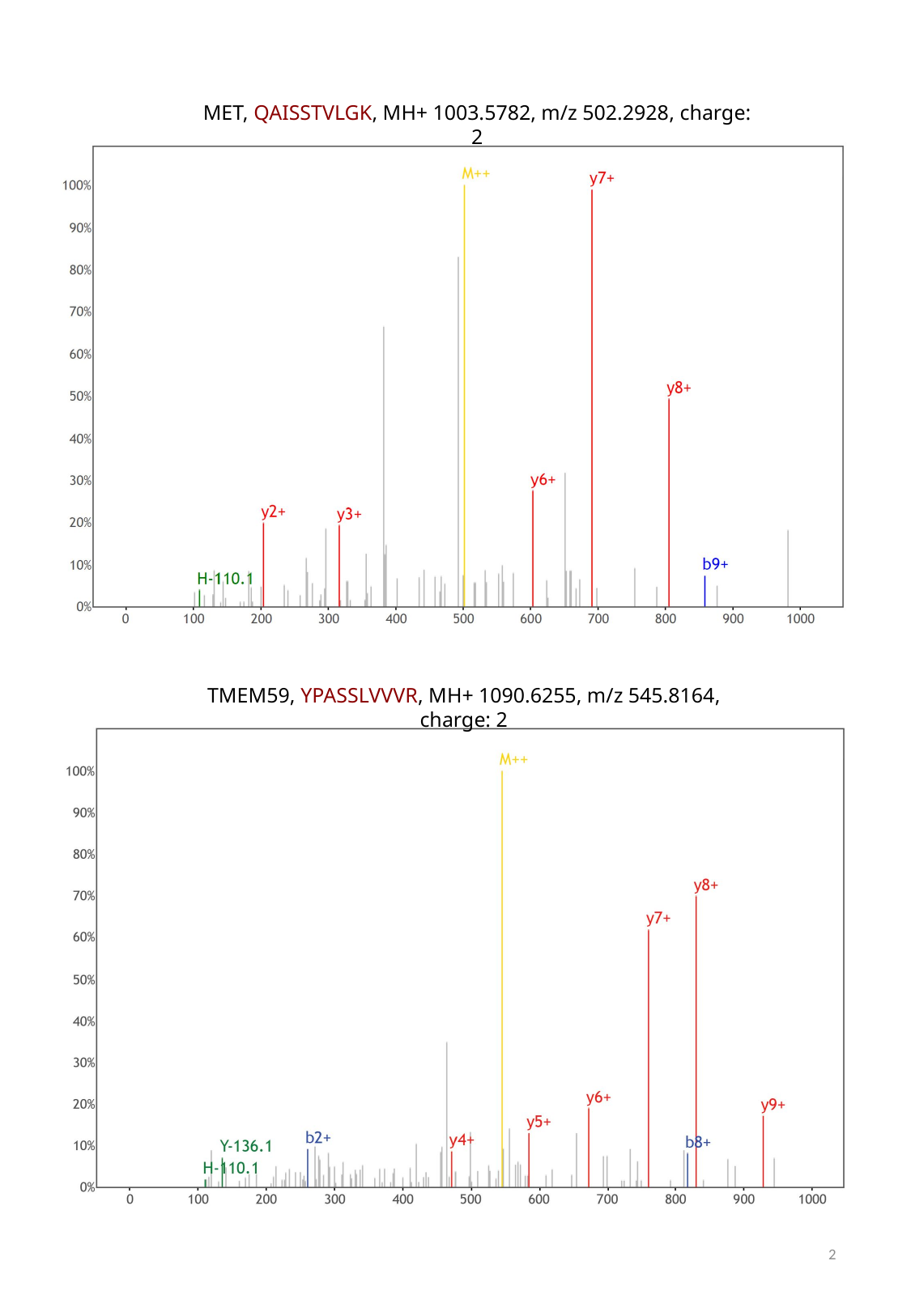

MET, QAISSTVLGK, MH+ 1003.5782, m/z 502.2928, charge: 2
TMEM59, YPASSLVVVR, MH+ 1090.6255, m/z 545.8164, charge: 2
2

## Slide 3
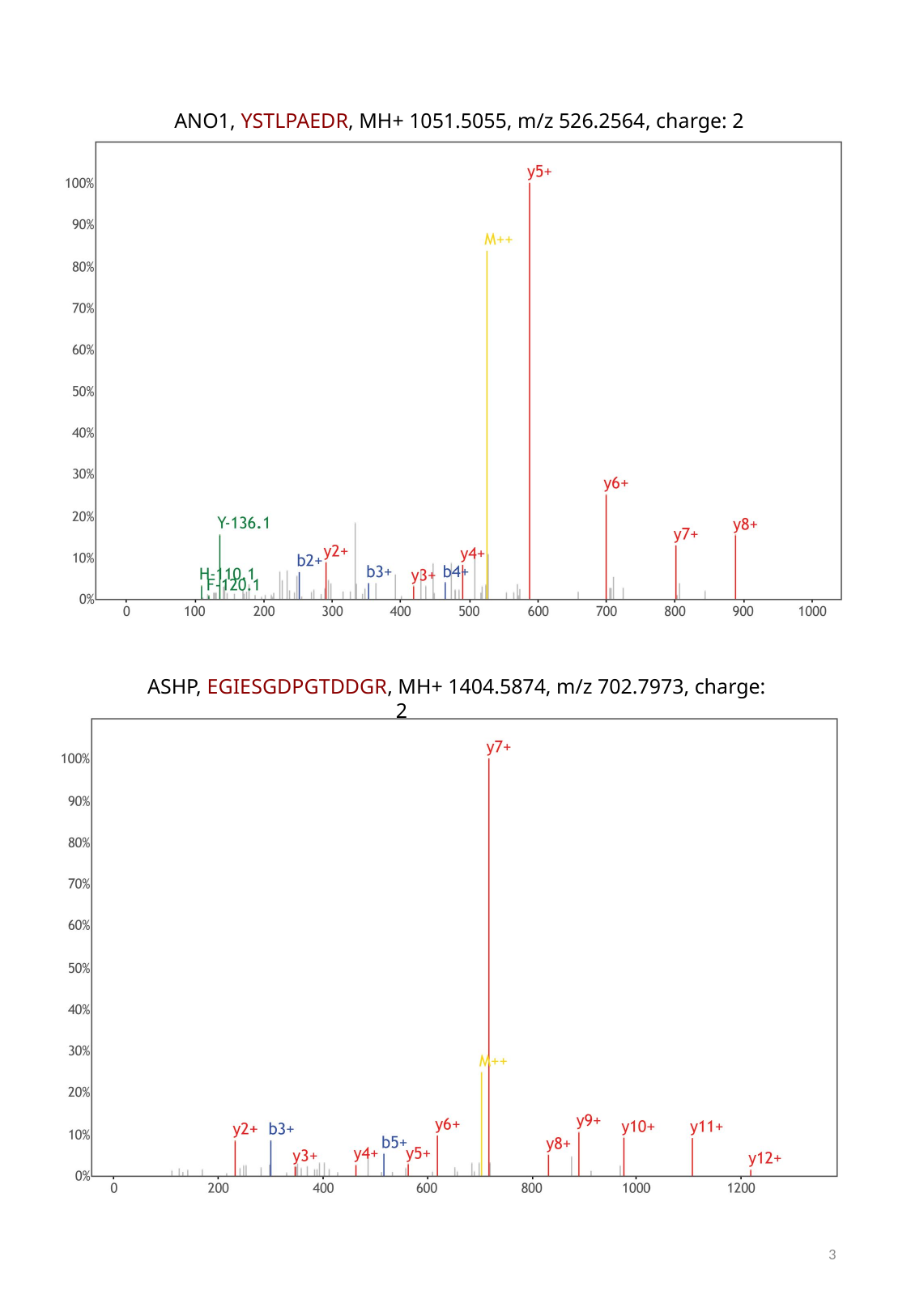

ANO1, YSTLPAEDR, MH+ 1051.5055, m/z 526.2564, charge: 2
ASHP, EGIESGDPGTDDGR, MH+ 1404.5874, m/z 702.7973, charge: 2
3

## Slide 4
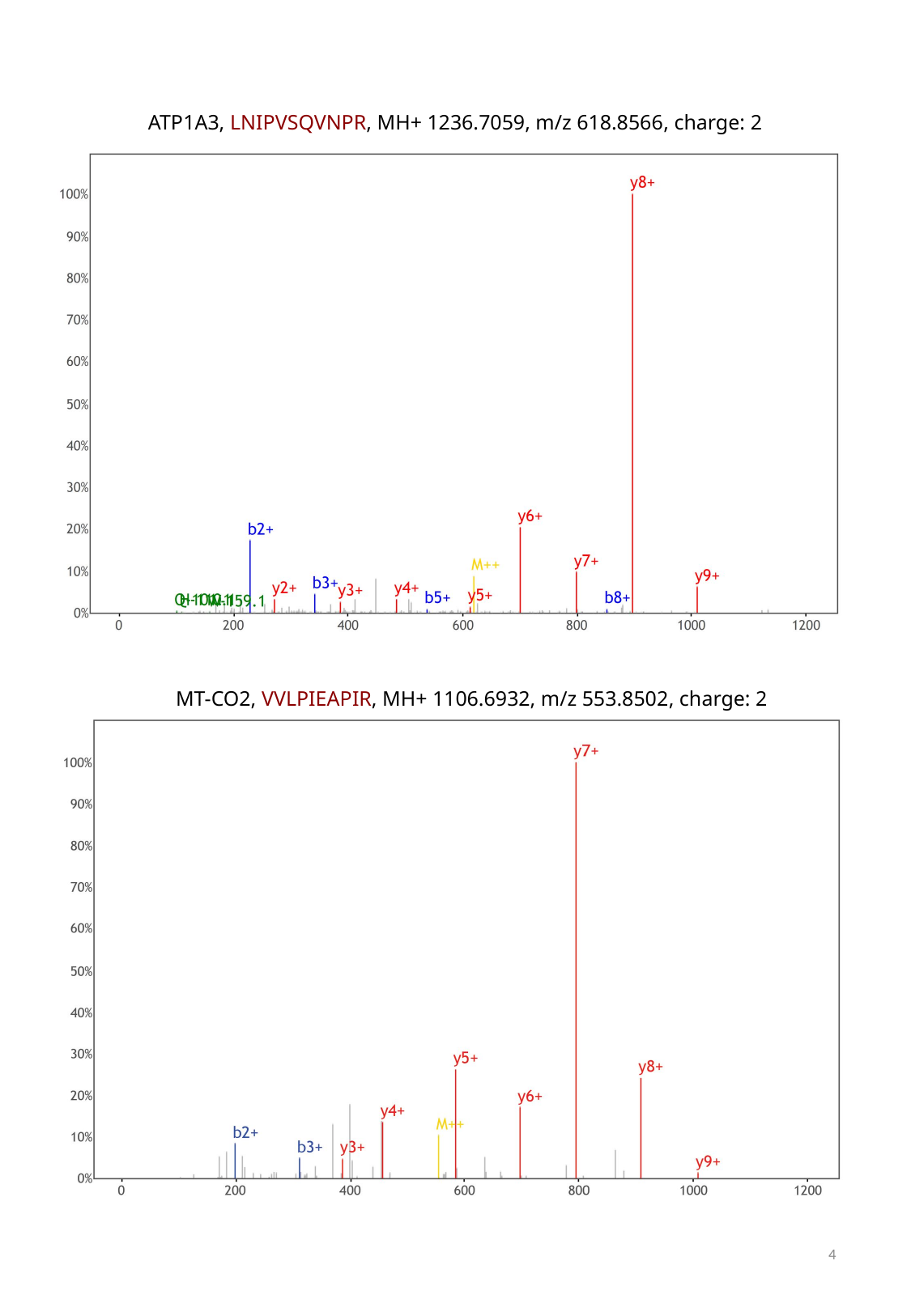

ATP1A3, LNIPVSQVNPR, MH+ 1236.7059, m/z 618.8566, charge: 2
MT-CO2, VVLPIEAPIR, MH+ 1106.6932, m/z 553.8502, charge: 2
4

## Slide 5
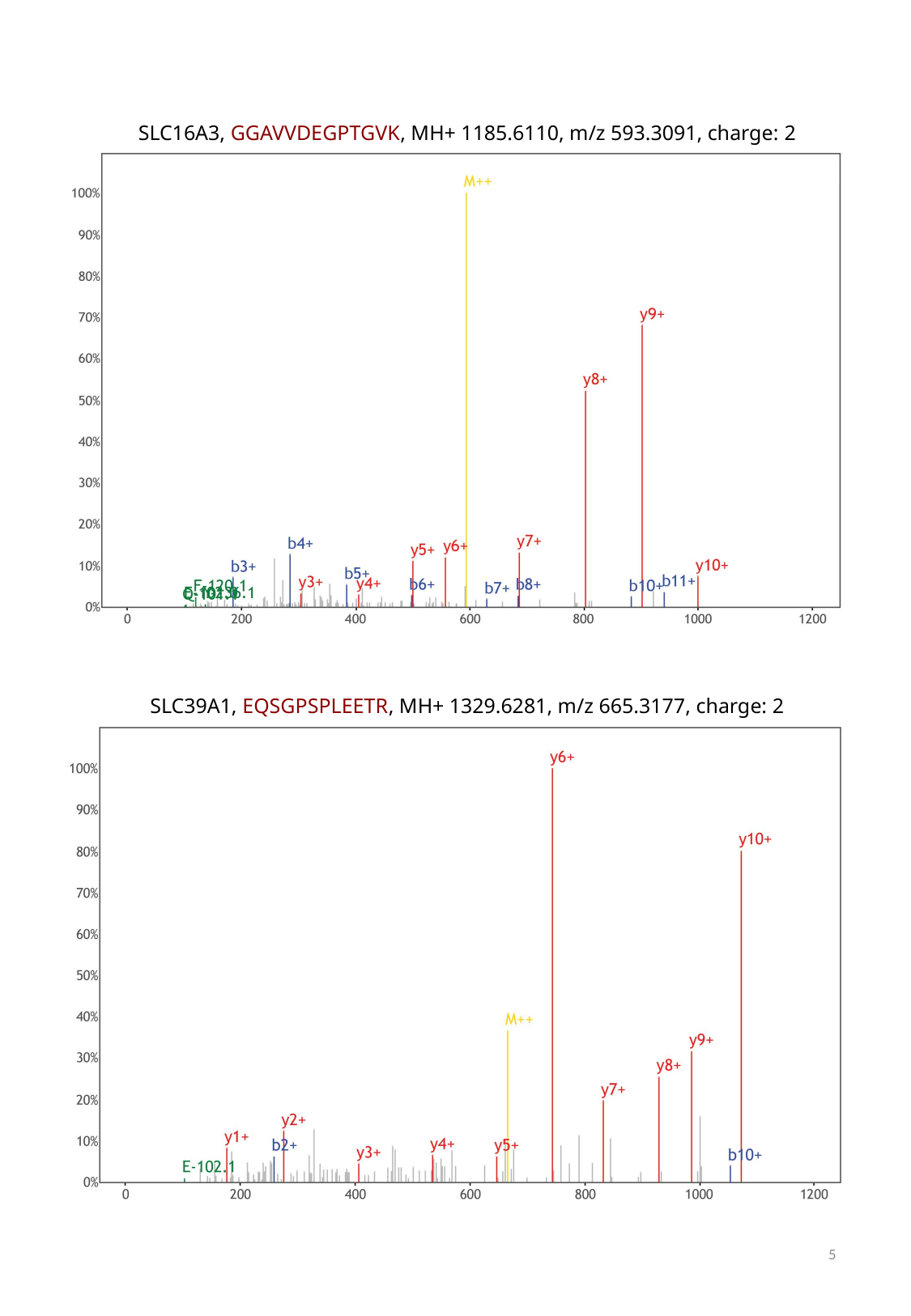

SLC16A3, GGAVVDEGPTGVK, MH+ 1185.6110, m/z 593.3091, charge: 2
SLC39A1, EQSGPSPLEETR, MH+ 1329.6281, m/z 665.3177, charge: 2
5

## Slide 6
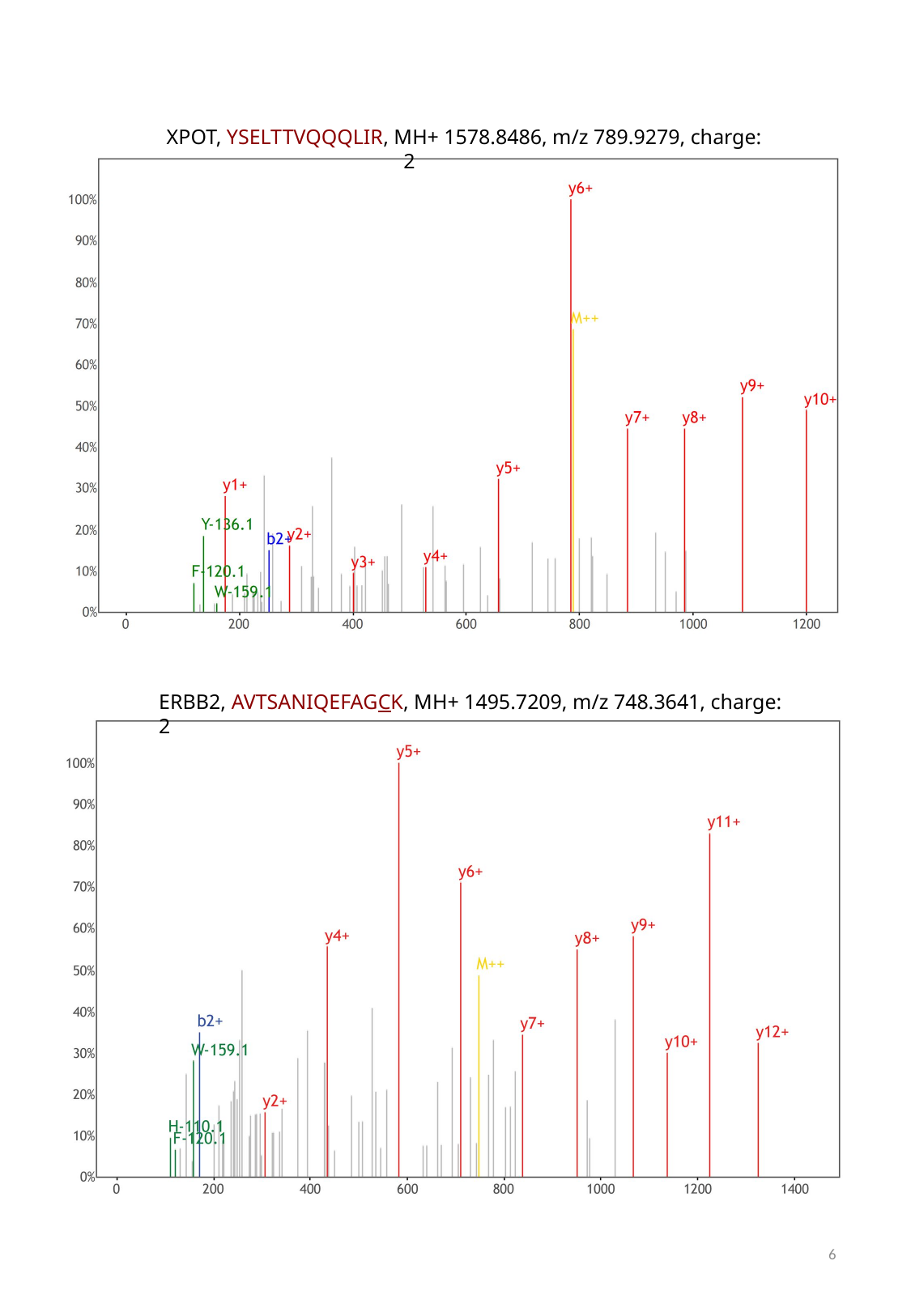

XPOT, YSELTTVQQQLIR, MH+ 1578.8486, m/z 789.9279, charge: 2
ERBB2, AVTSANIQEFAGCK, MH+ 1495.7209, m/z 748.3641, charge: 2
6

## Slide 7
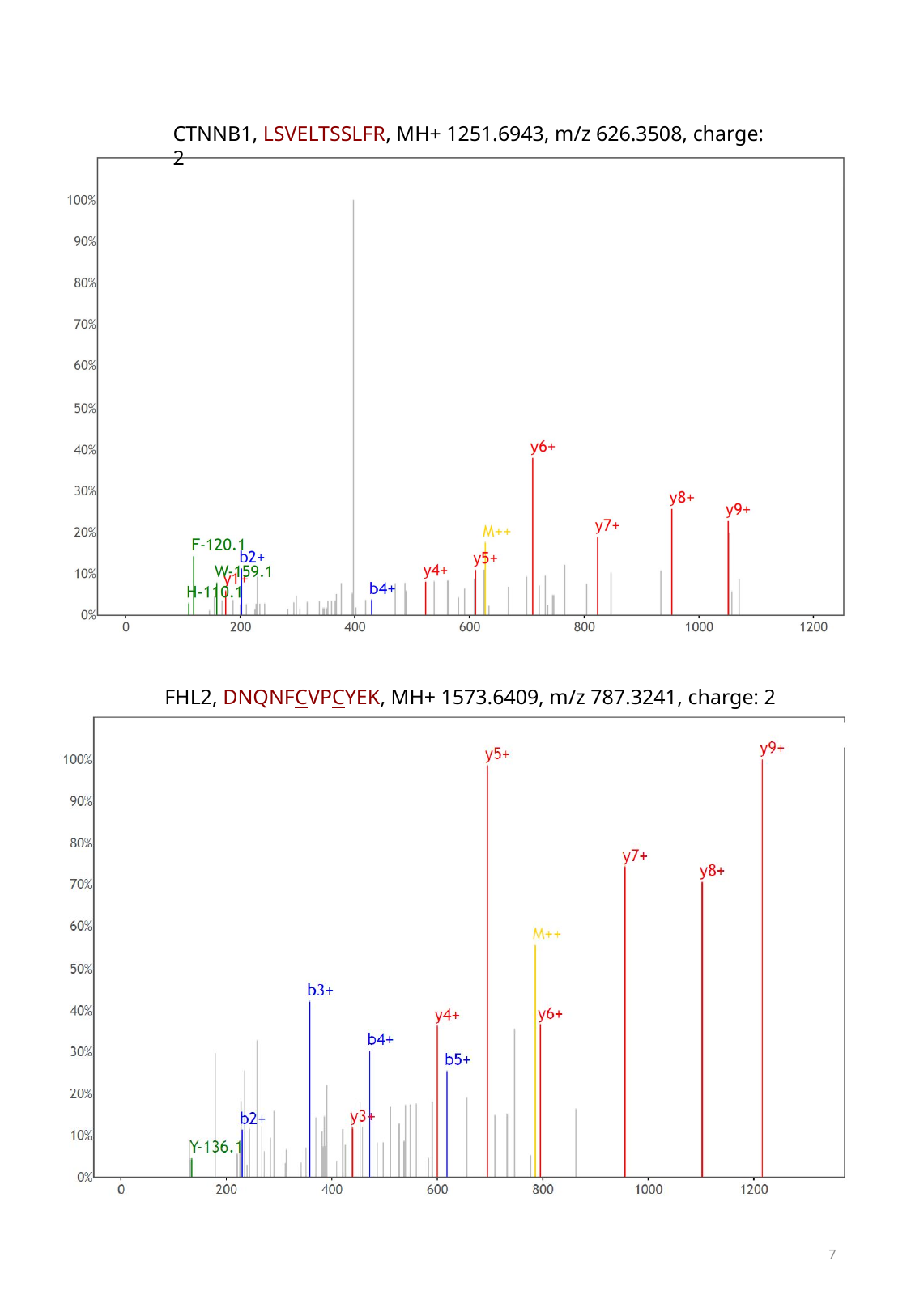

CTNNB1, LSVELTSSLFR, MH+ 1251.6943, m/z 626.3508, charge: 2
FHL2, DNQNFCVPCYEK, MH+ 1573.6409, m/z 787.3241, charge: 2
7

## Slide 8
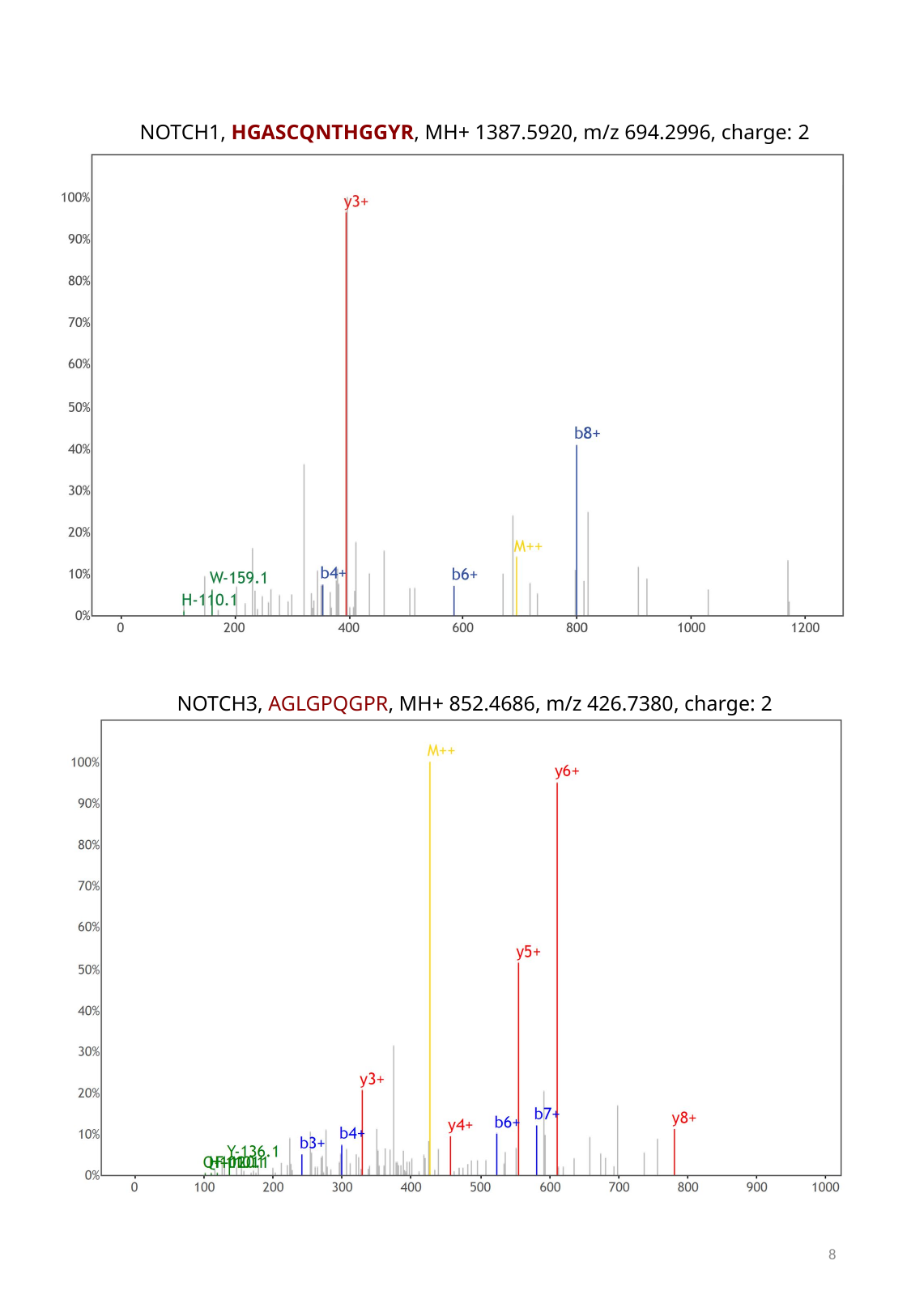

NOTCH1, HGASCQNTHGGYR, MH+ 1387.5920, m/z 694.2996, charge: 2
NOTCH3, AGLGPQGPR, MH+ 852.4686, m/z 426.7380, charge: 2
8

## Slide 9
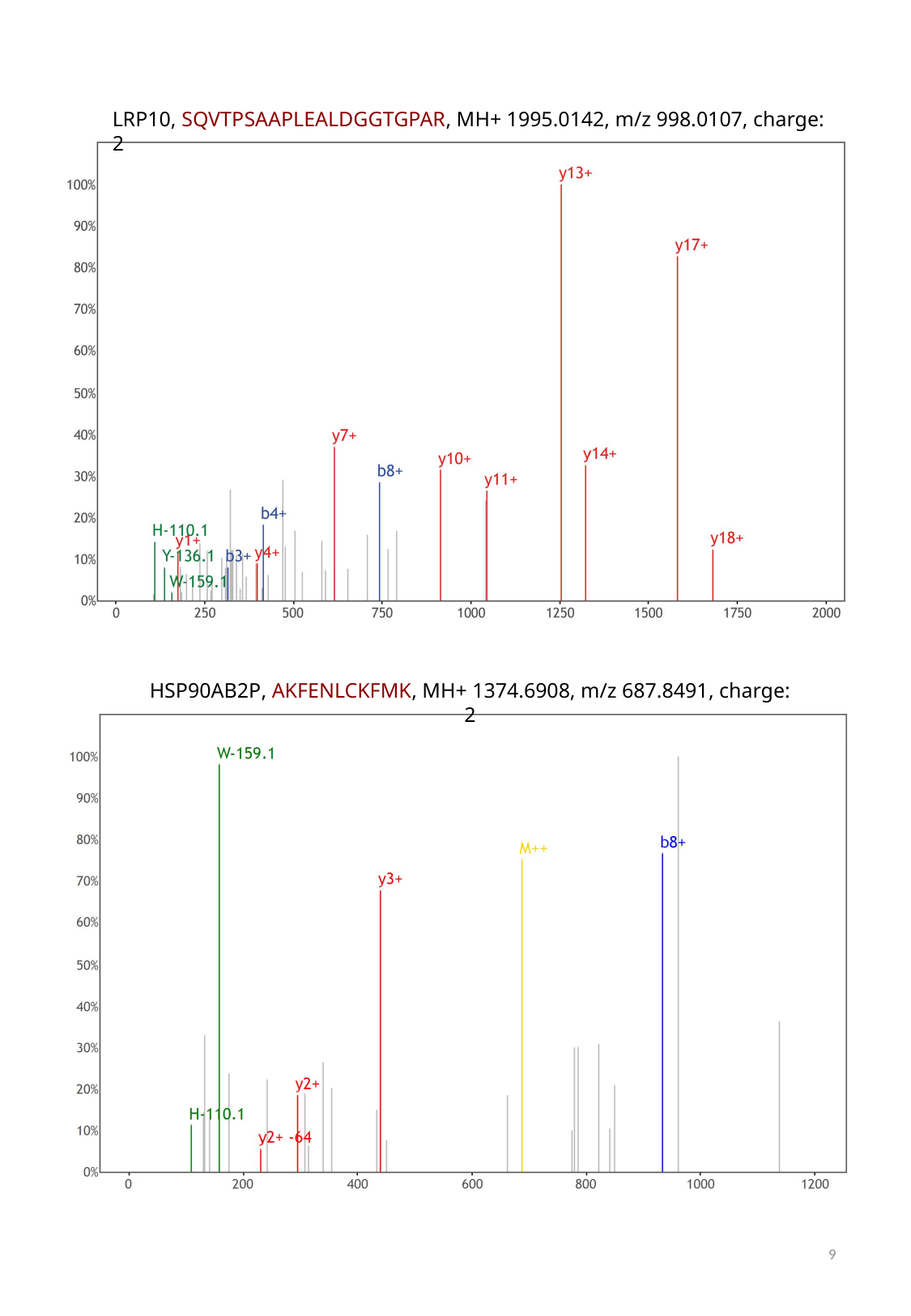

LRP10, SQVTPSAAPLEALDGGTGPAR, MH+ 1995.0142, m/z 998.0107, charge: 2
HSP90AB2P, AKFENLCKFMK, MH+ 1374.6908, m/z 687.8491, charge: 2
9

## Slide 10
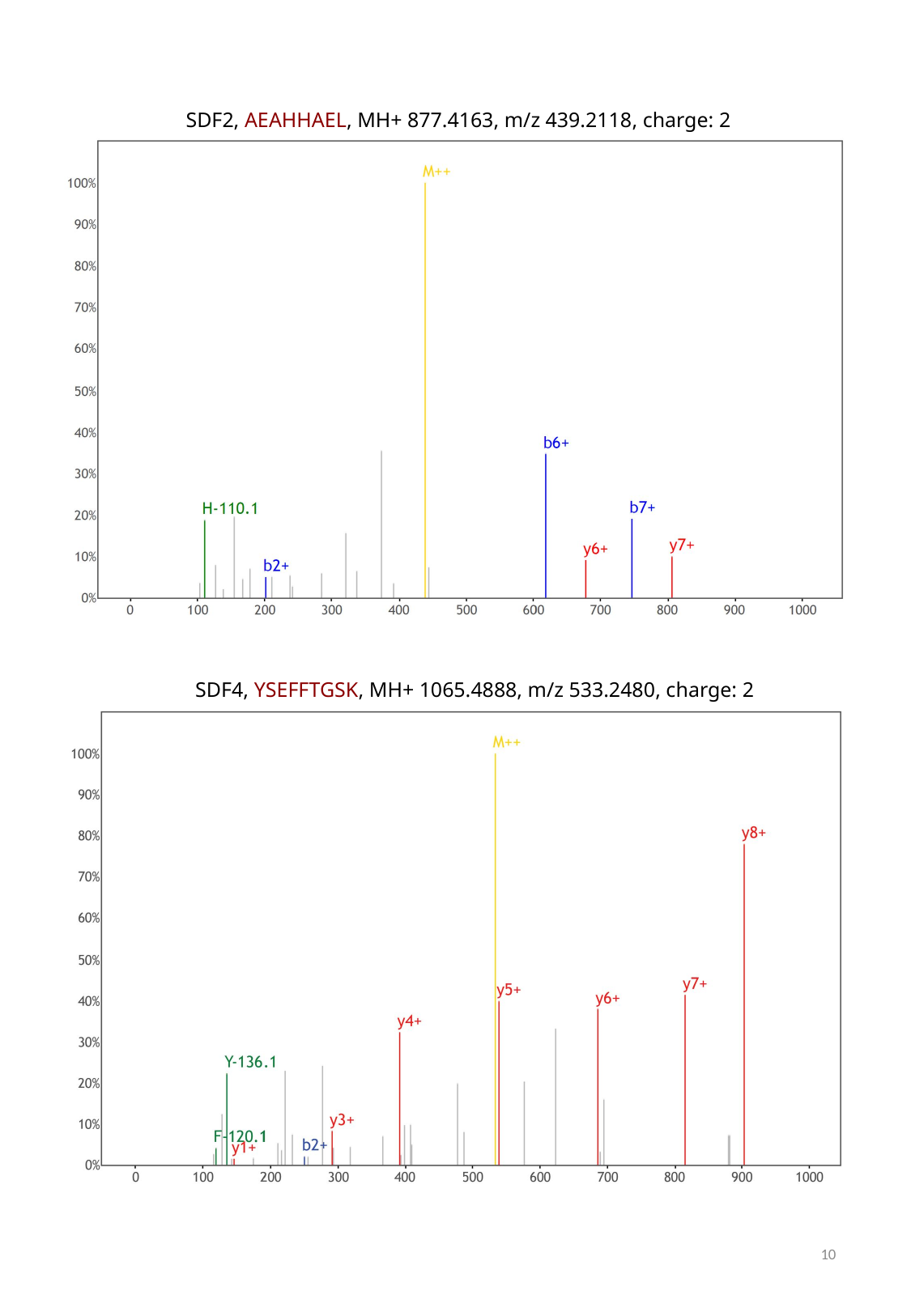

SDF2, AEAHHAEL, MH+ 877.4163, m/z 439.2118, charge: 2
SDF4, YSEFFTGSK, MH+ 1065.4888, m/z 533.2480, charge: 2
10

## Slide 11
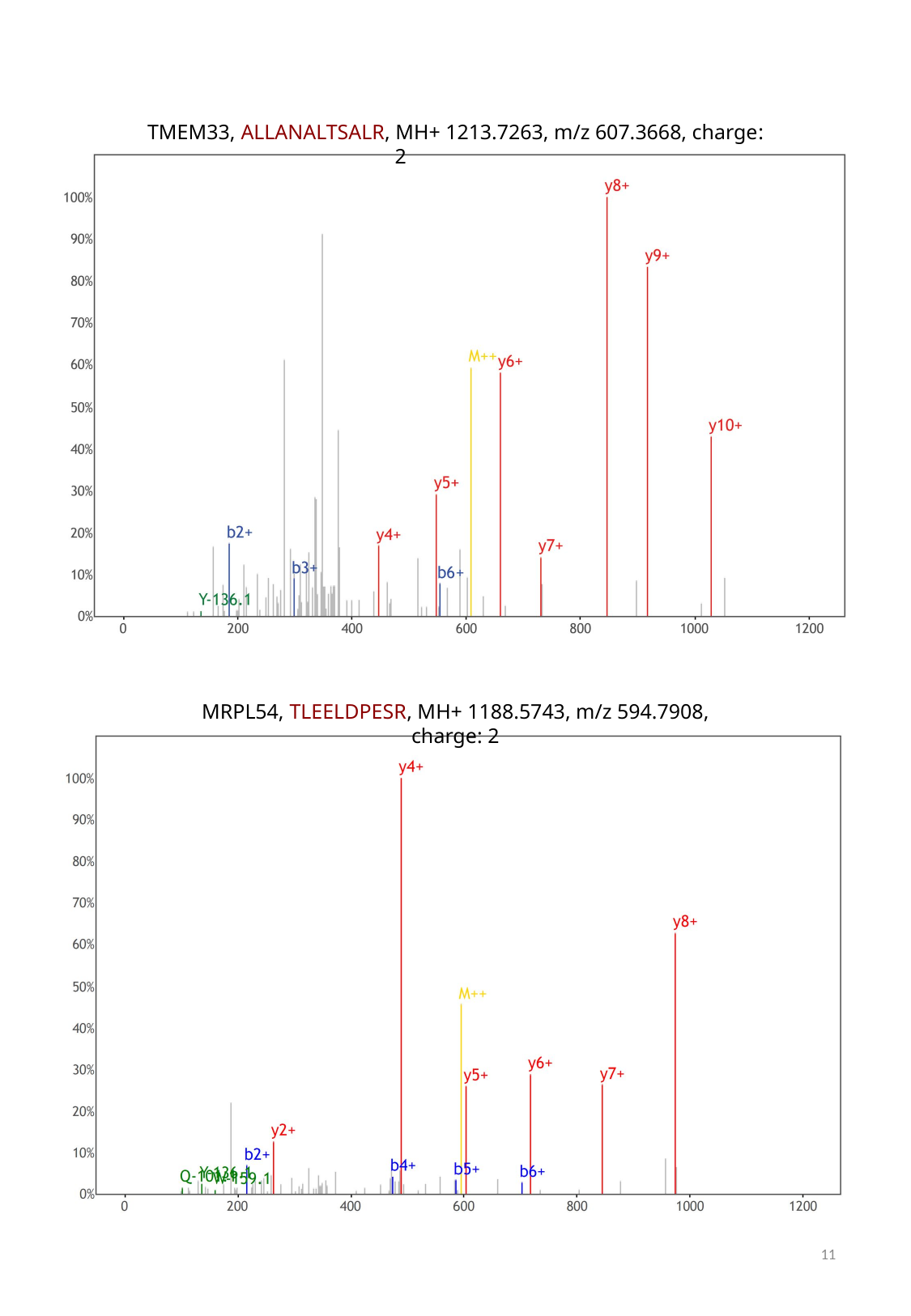

TMEM33, ALLANALTSALR, MH+ 1213.7263, m/z 607.3668, charge: 2
MRPL54, TLEELDPESR, MH+ 1188.5743, m/z 594.7908, charge: 2
11

## Slide 12
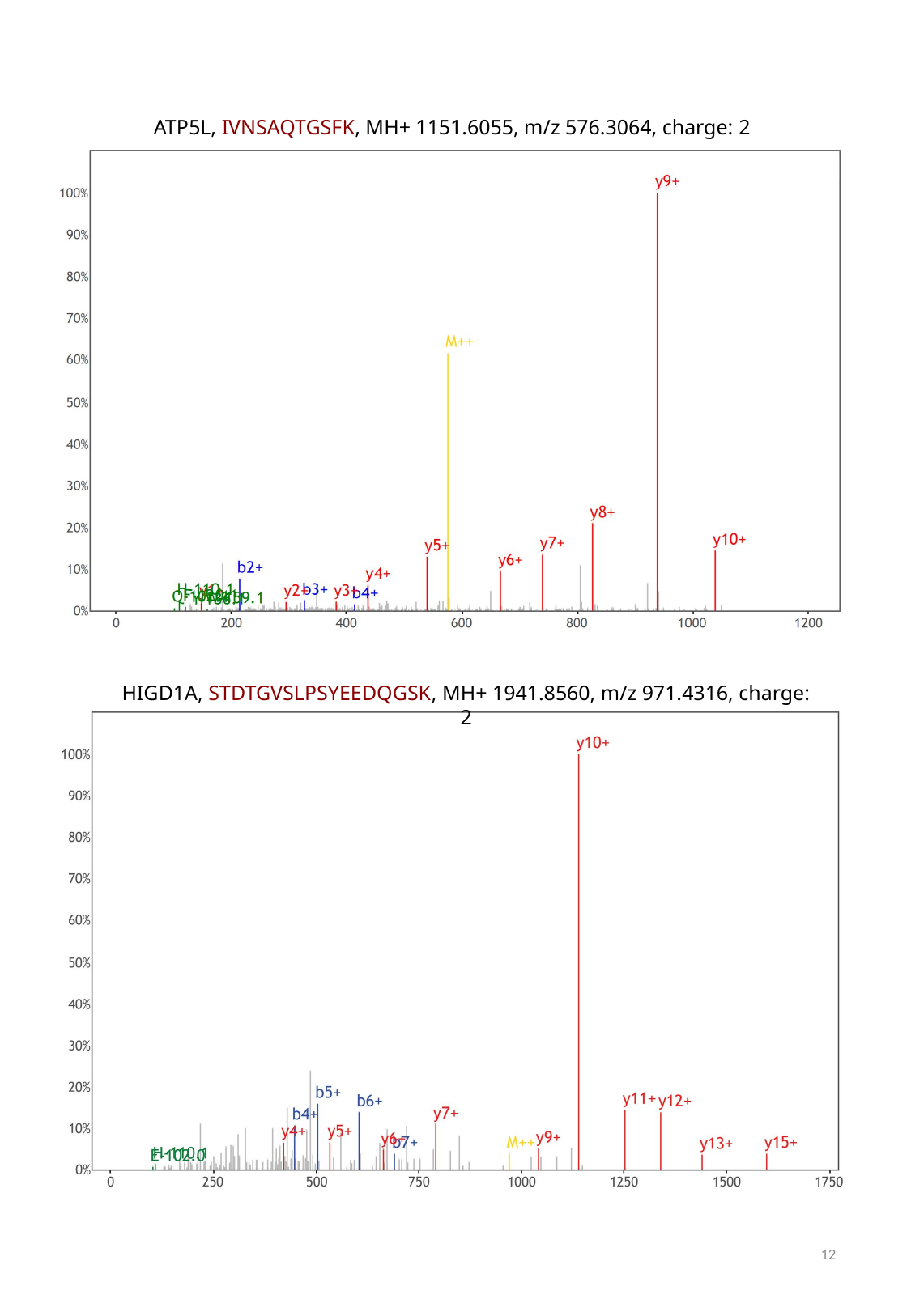

ATP5L, IVNSAQTGSFK, MH+ 1151.6055, m/z 576.3064, charge: 2
HIGD1A, STDTGVSLPSYEEDQGSK, MH+ 1941.8560, m/z 971.4316, charge: 2
12

## Slide 13
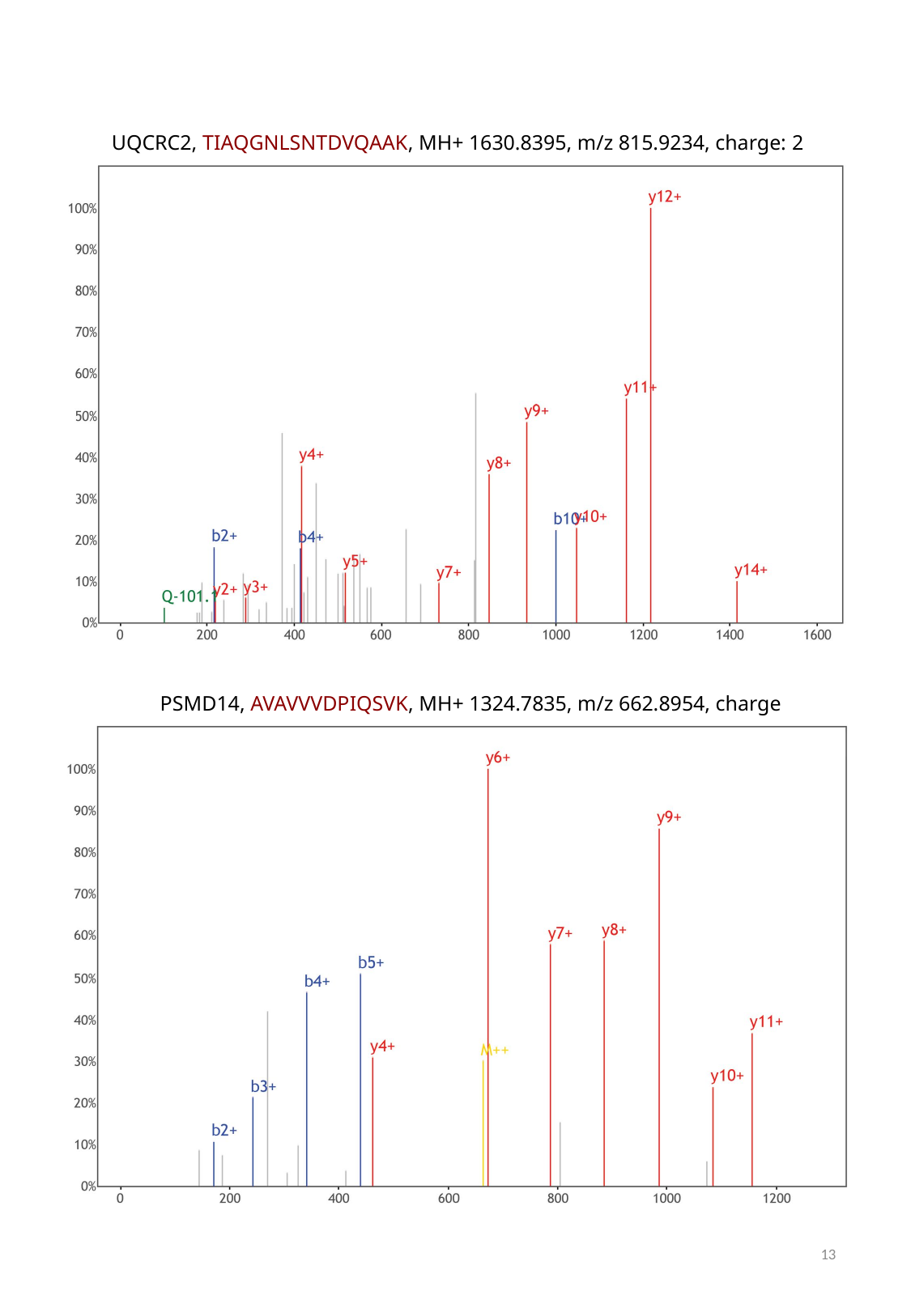

UQCRC2, TIAQGNLSNTDVQAAK, MH+ 1630.8395, m/z 815.9234, charge: 2
PSMD14, AVAVVVDPIQSVK, MH+ 1324.7835, m/z 662.8954, charge
13

## Slide 14
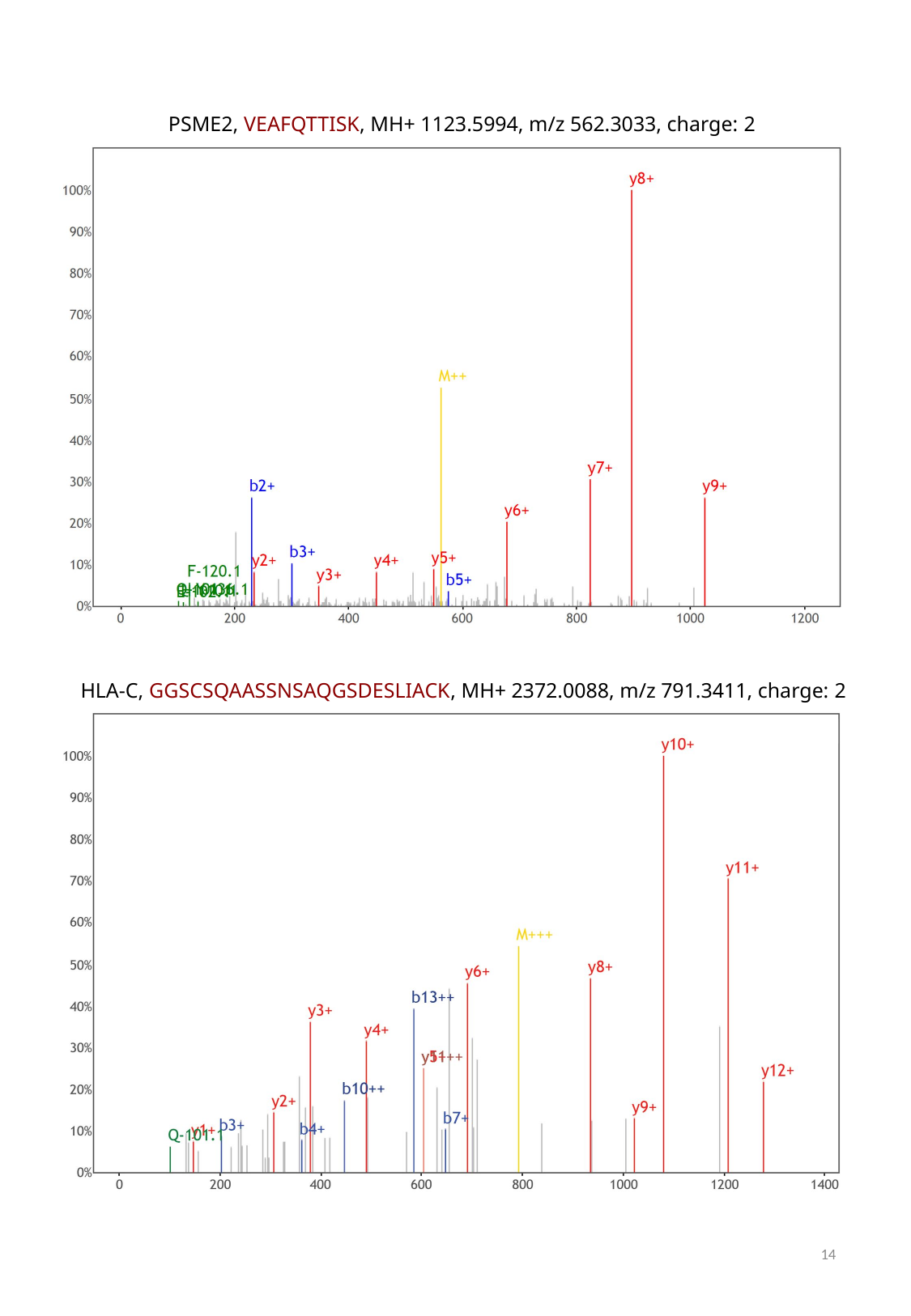

PSME2, VEAFQTTISK, MH+ 1123.5994, m/z 562.3033, charge: 2
HLA-C, GGSCSQAASSNSAQGSDESLIACK, MH+ 2372.0088, m/z 791.3411, charge: 2
14

## Slide 15
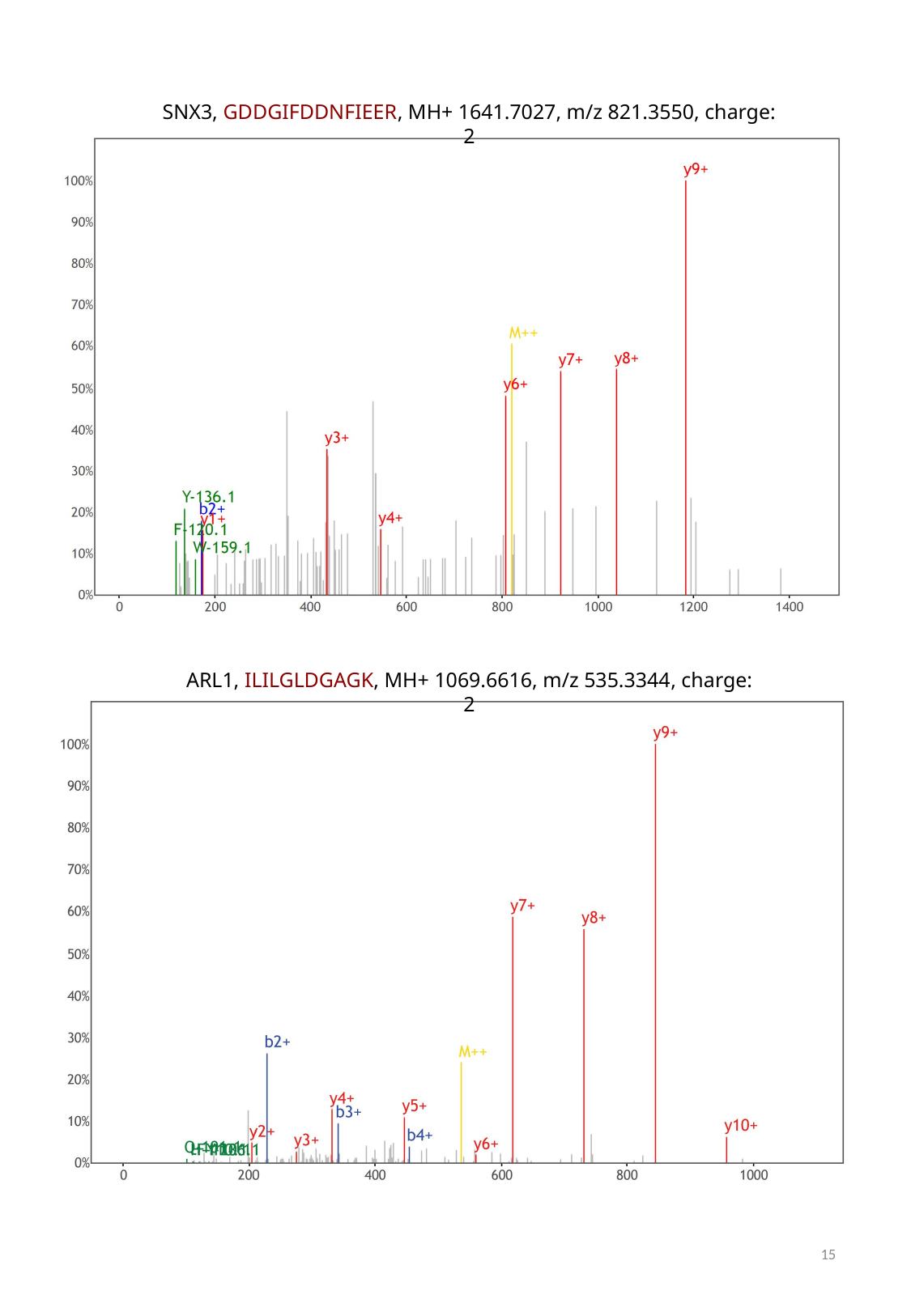

SNX3, GDDGIFDDNFIEER, MH+ 1641.7027, m/z 821.3550, charge: 2
ARL1, ILILGLDGAGK, MH+ 1069.6616, m/z 535.3344, charge: 2
15

## Slide 16
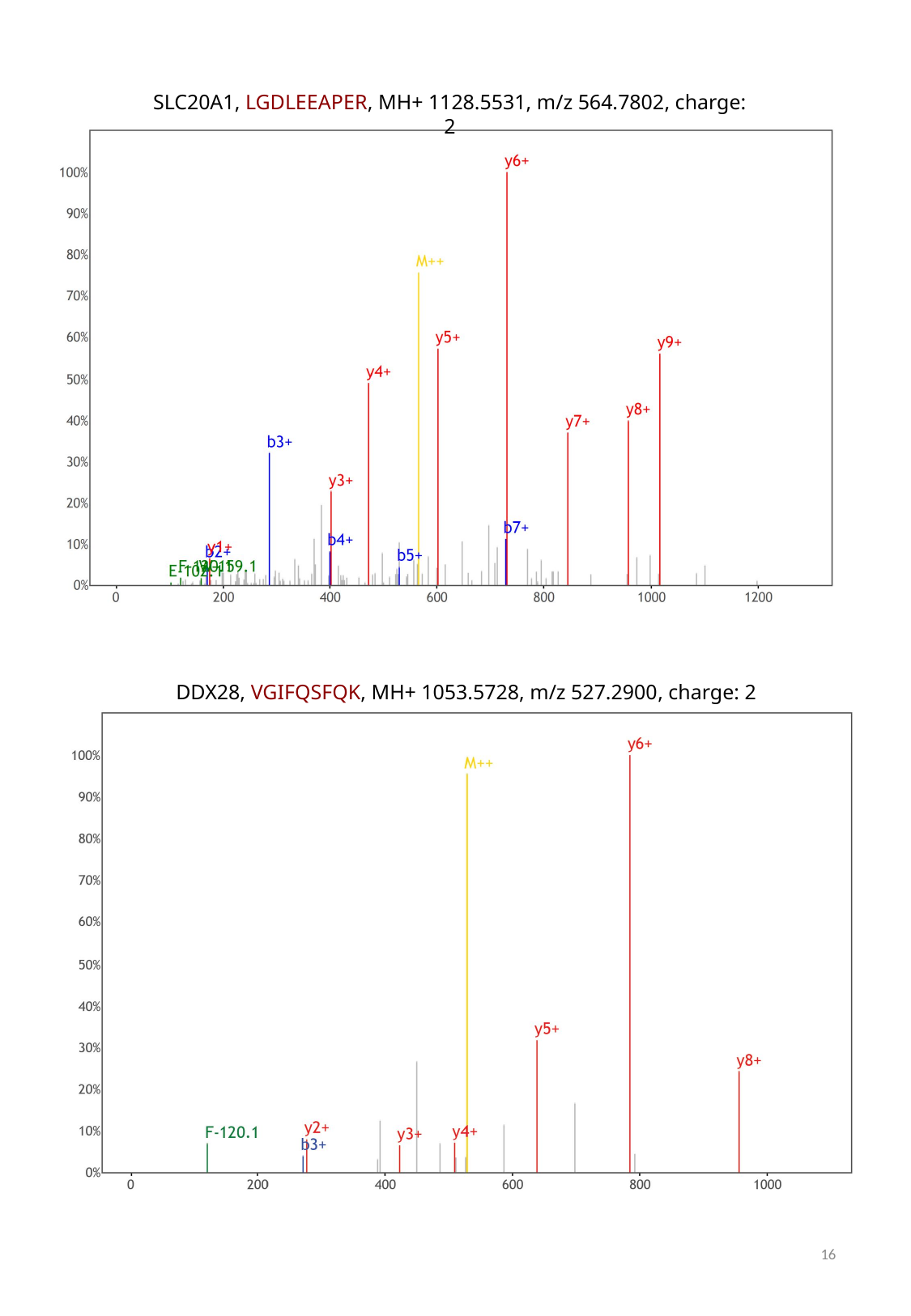

SLC20A1, LGDLEEAPER, MH+ 1128.5531, m/z 564.7802, charge: 2
DDX28, VGIFQSFQK, MH+ 1053.5728, m/z 527.2900, charge: 2
16

## Slide 17
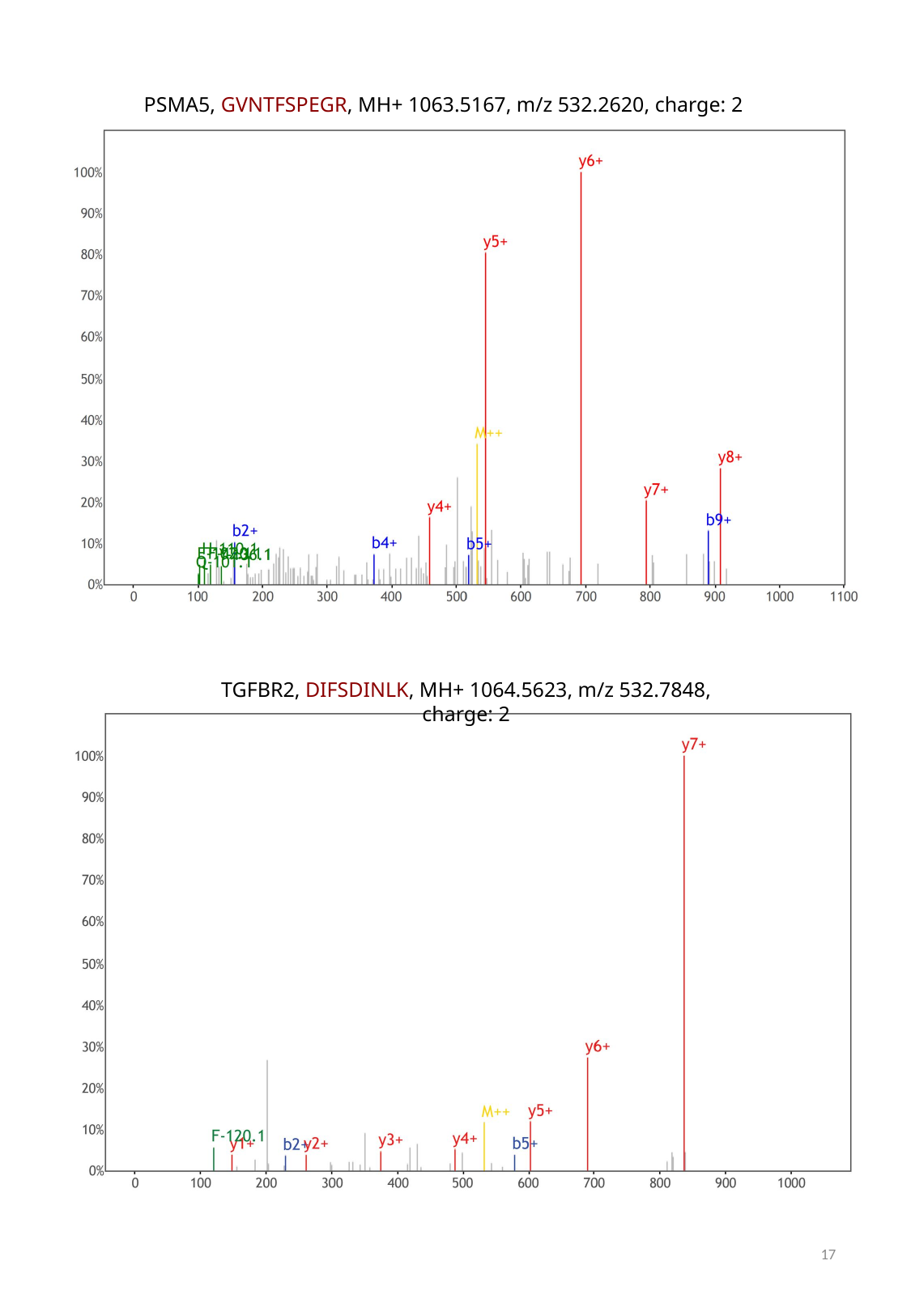

PSMA5, GVNTFSPEGR, MH+ 1063.5167, m/z 532.2620, charge: 2
TGFBR2, DIFSDINLK, MH+ 1064.5623, m/z 532.7848, charge: 2
17

## Slide 18
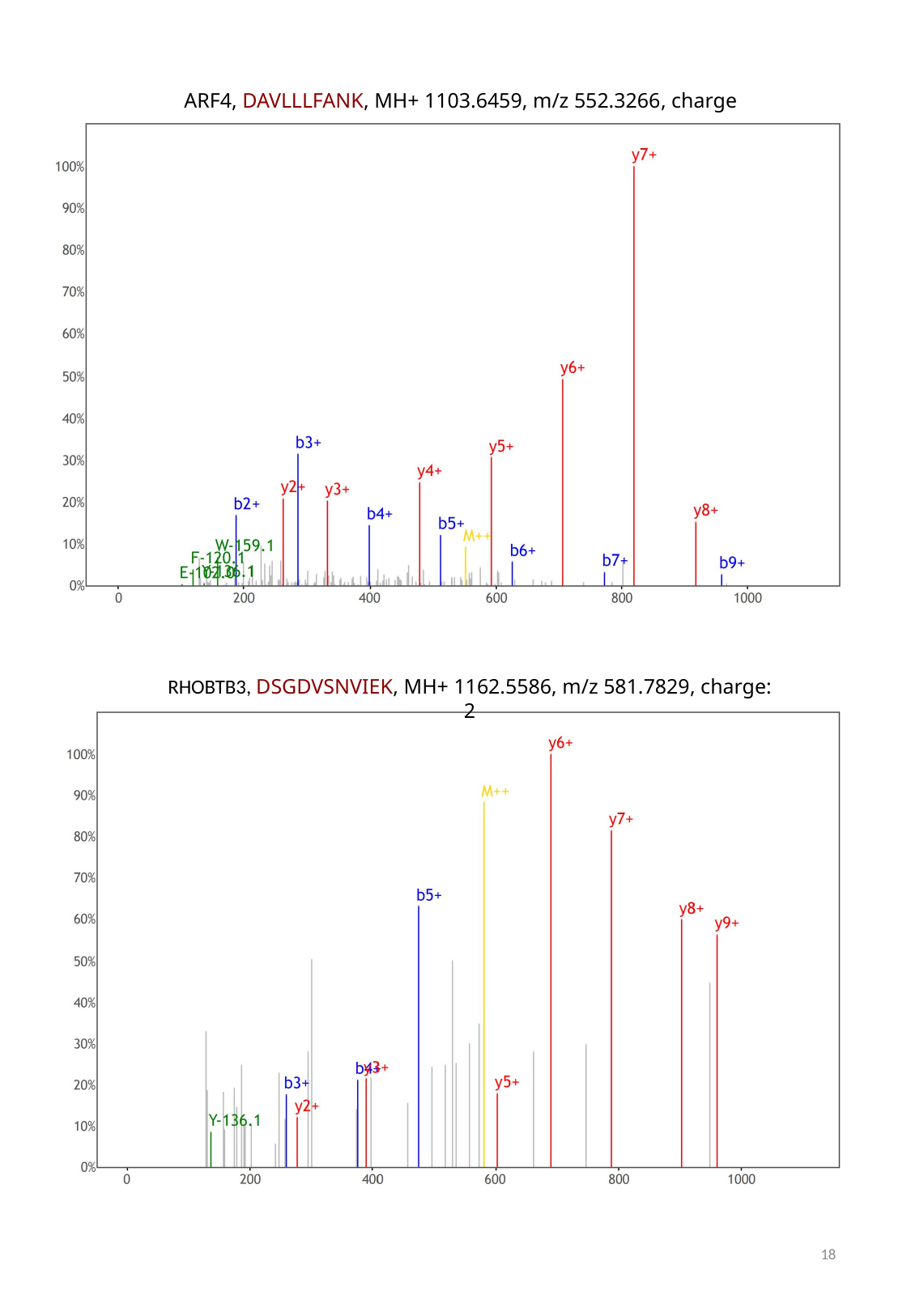

ARF4, DAVLLLFANK, MH+ 1103.6459, m/z 552.3266, charge
RHOBTB3, DSGDVSNVIEK, MH+ 1162.5586, m/z 581.7829, charge: 2
18

## Slide 19
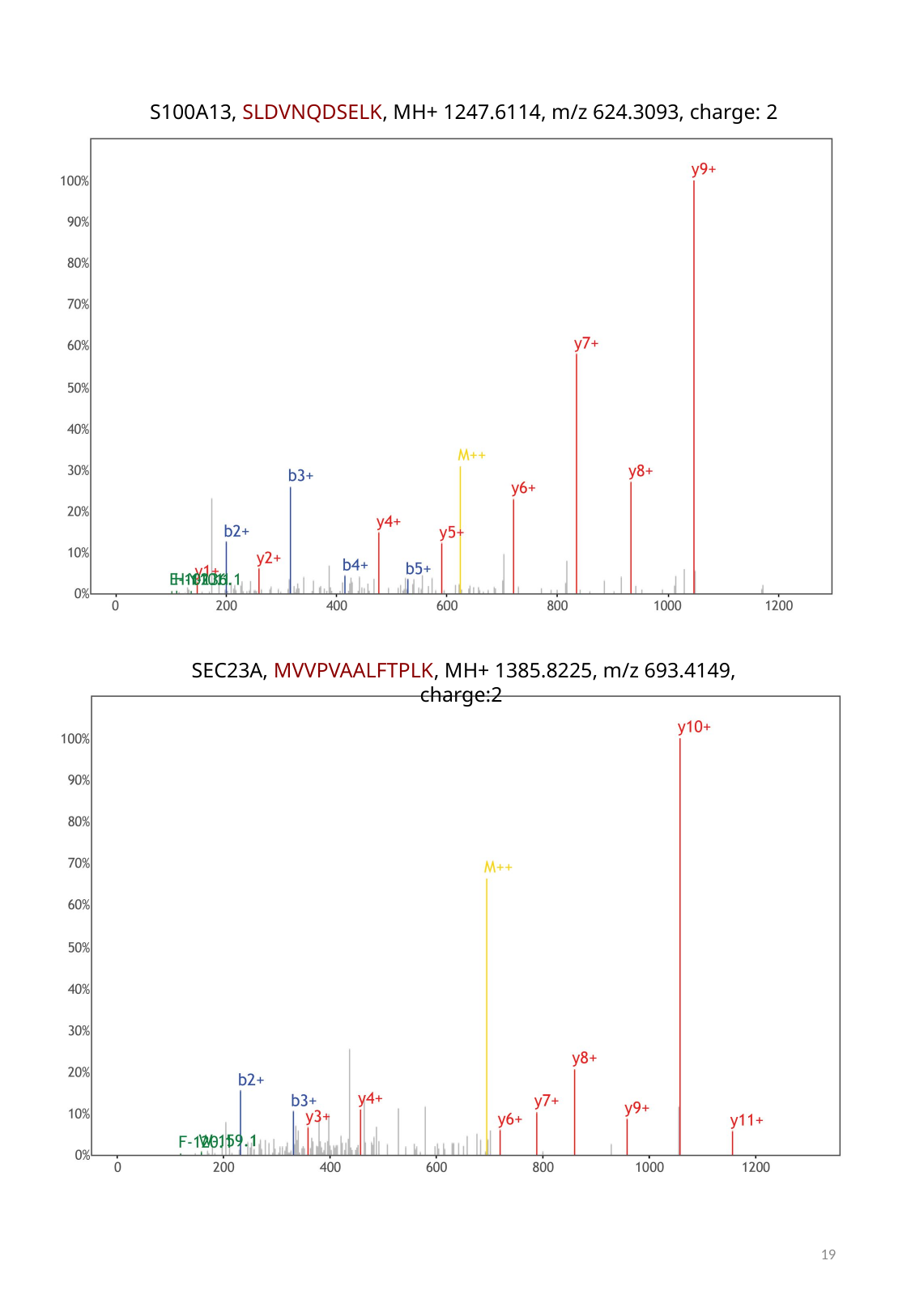

S100A13, SLDVNQDSELK, MH+ 1247.6114, m/z 624.3093, charge: 2
SEC23A, MVVPVAALFTPLK, MH+ 1385.8225, m/z 693.4149, charge:2
19

## Slide 20
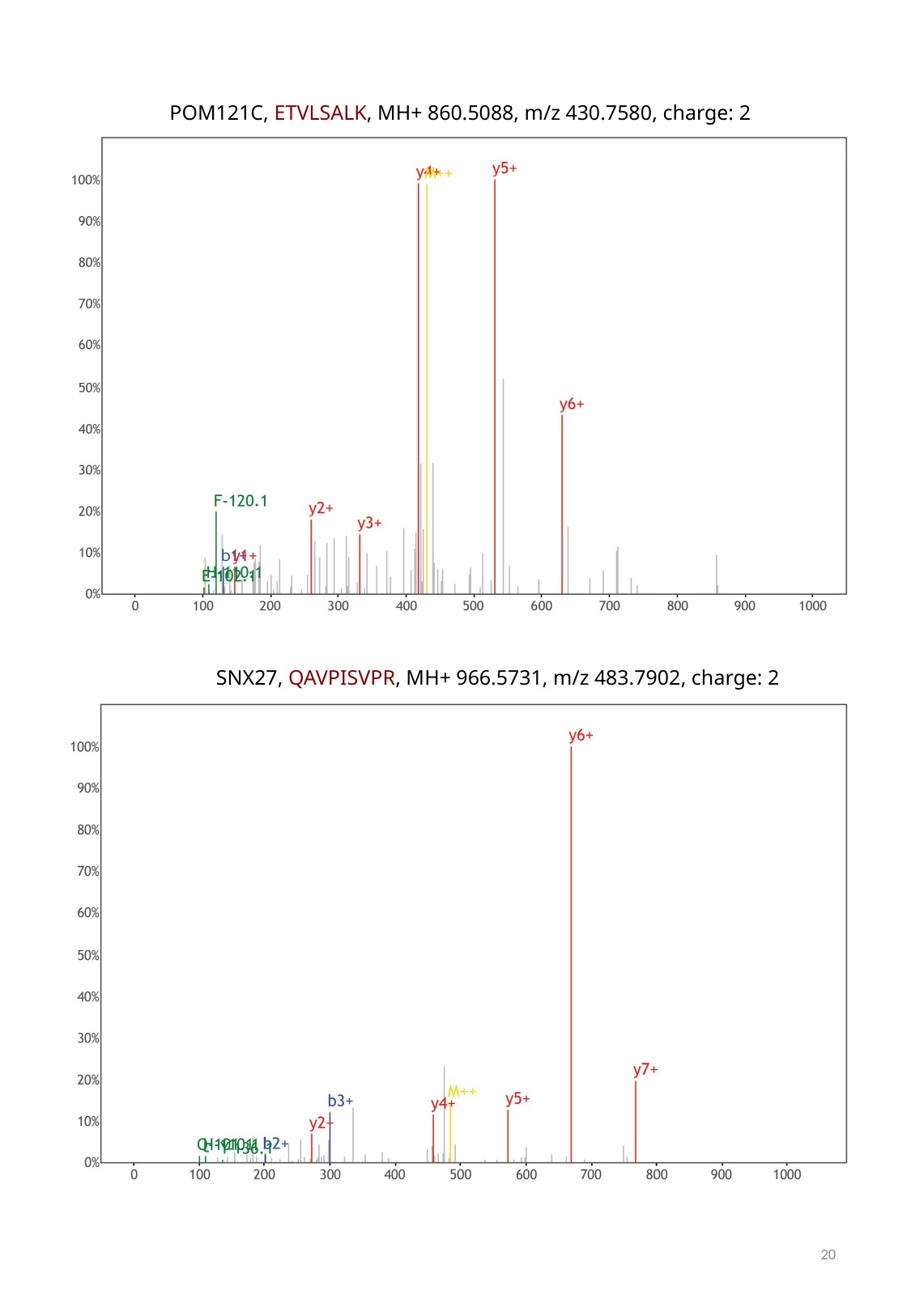

POM121C, ETVLSALK, MH+ 860.5088, m/z 430.7580, charge: 2
SNX27, QAVPISVPR, MH+ 966.5731, m/z 483.7902, charge: 2
20

## Slide 21
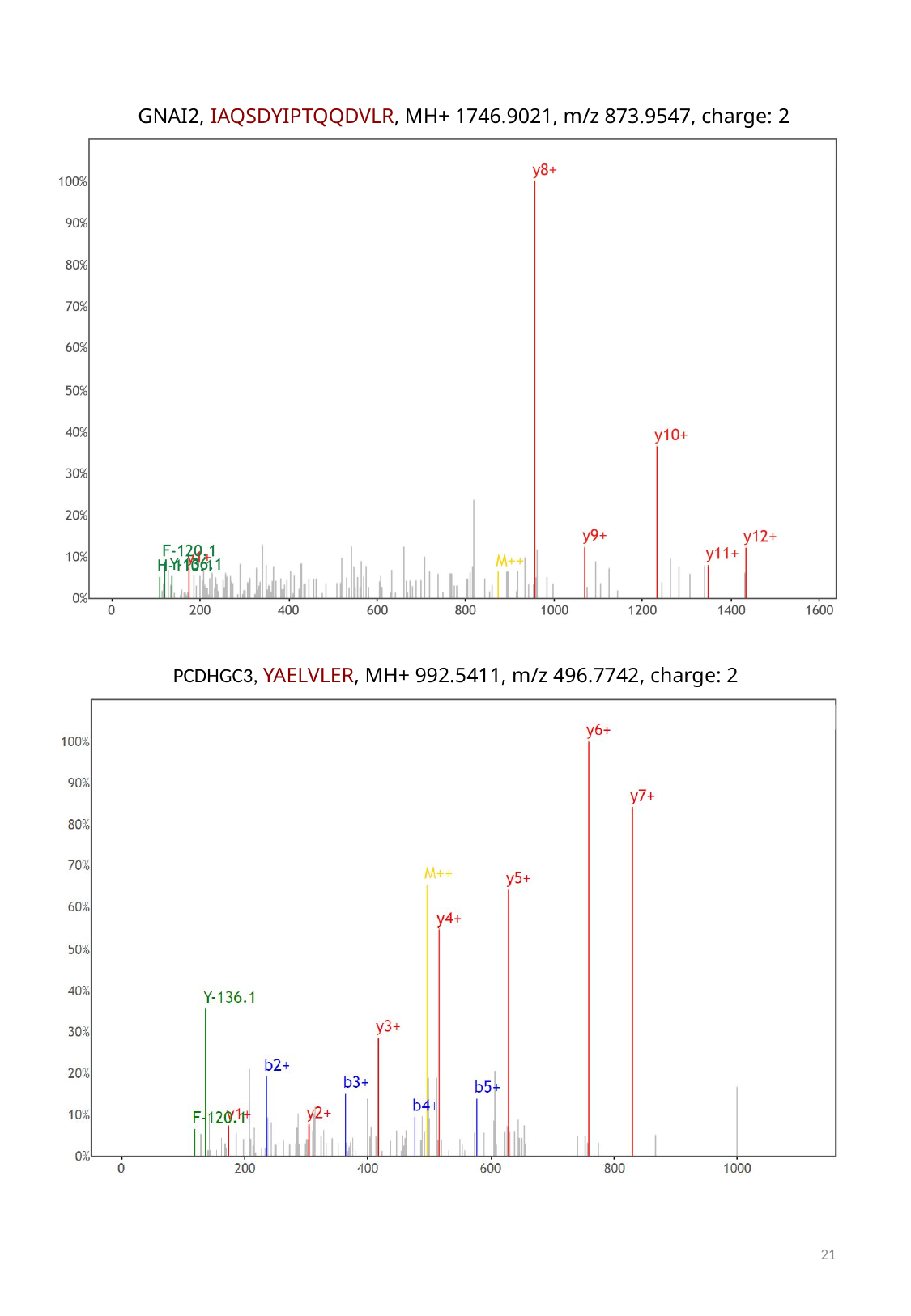

GNAI2, IAQSDYIPTQQDVLR, MH+ 1746.9021, m/z 873.9547, charge: 2
PCDHGC3, YAELVLER, MH+ 992.5411, m/z 496.7742, charge: 2
21

## Slide 22
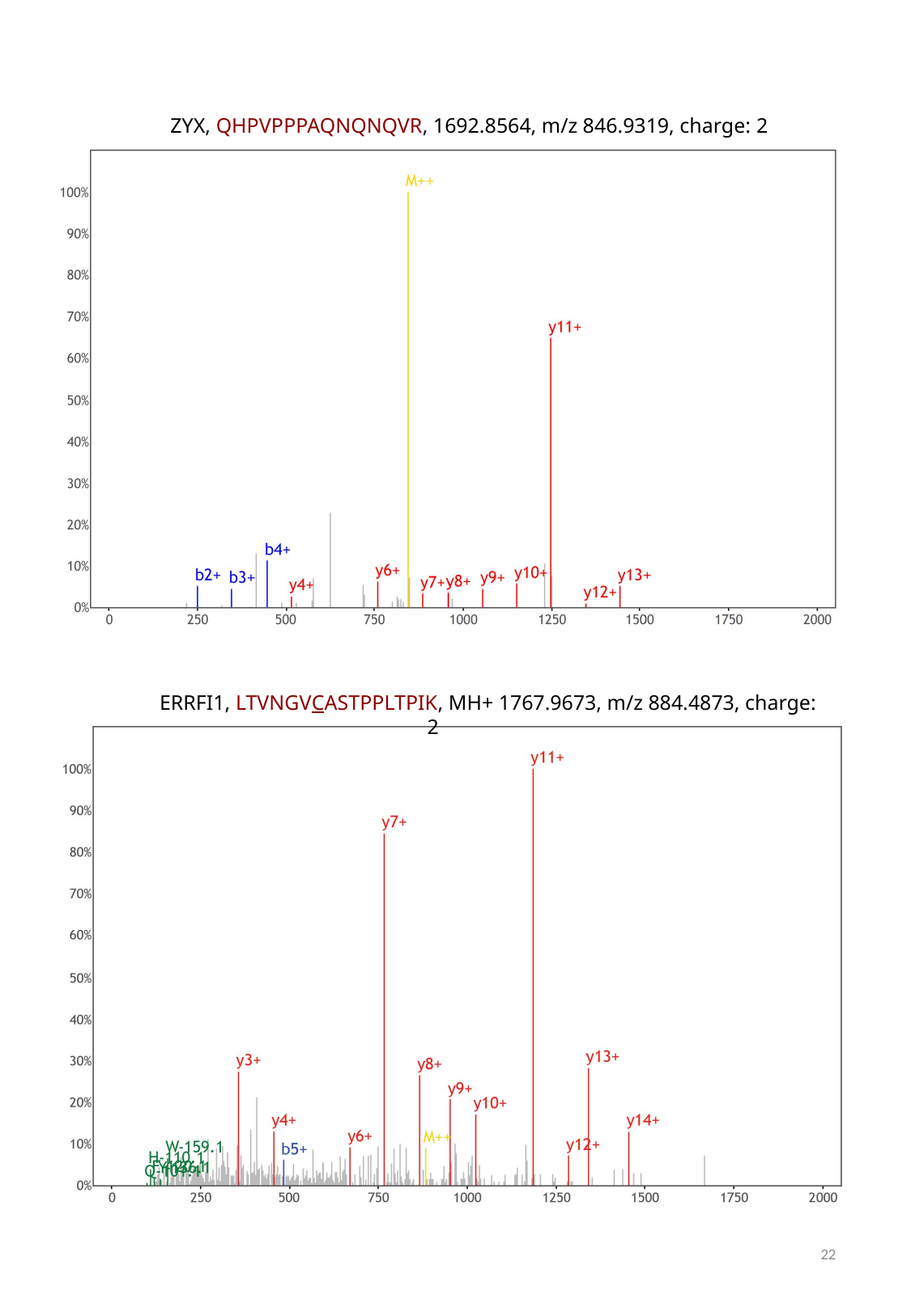

ZYX, QHPVPPPAQNQNQVR, 1692.8564, m/z 846.9319, charge: 2
ERRFI1, LTVNGVCASTPPLTPIK, MH+ 1767.9673, m/z 884.4873, charge: 2
22

## Slide 23
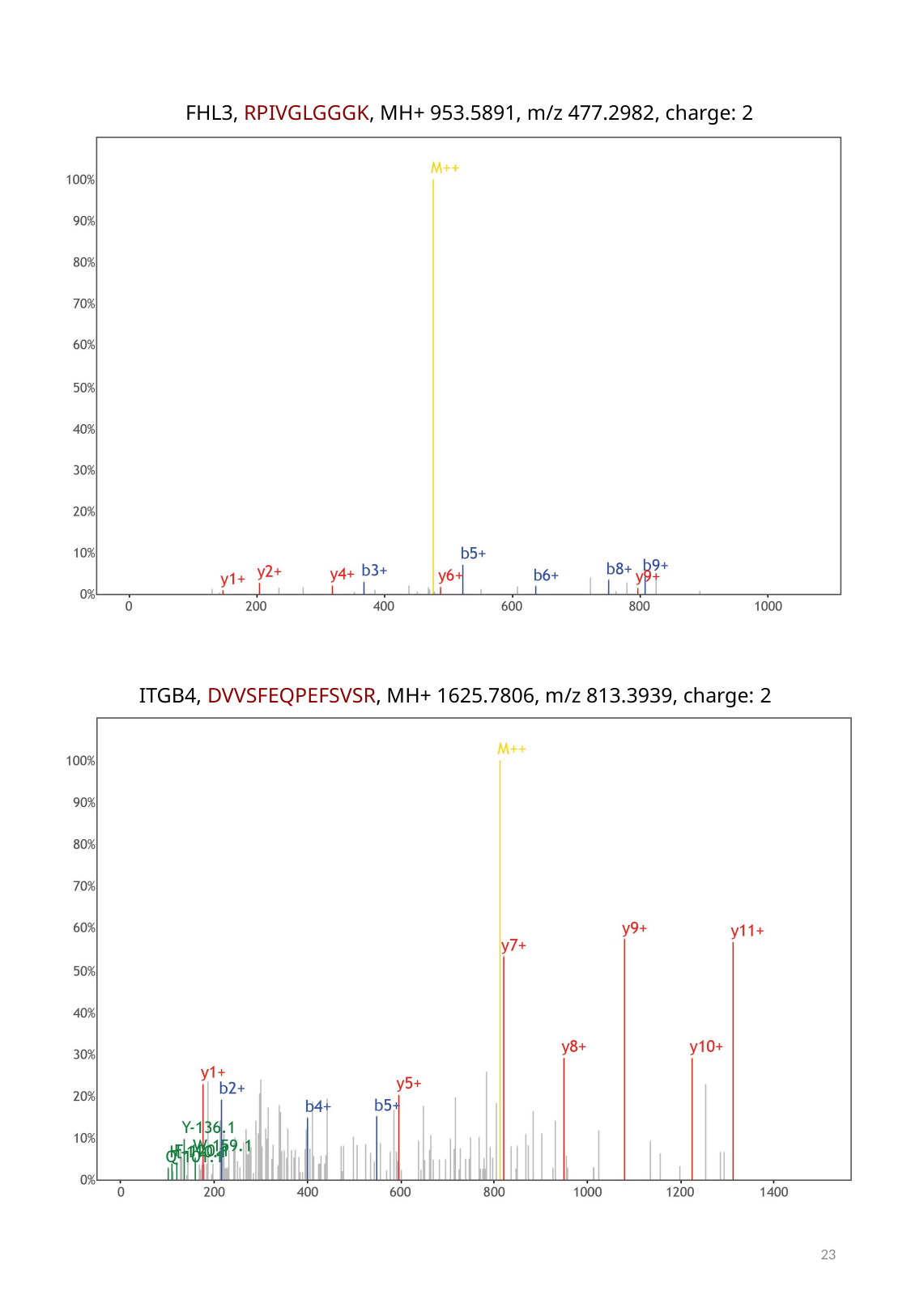

FHL3, RPIVGLGGGK, MH+ 953.5891, m/z 477.2982, charge: 2
ITGB4, DVVSFEQPEFSVSR, MH+ 1625.7806, m/z 813.3939, charge: 2
23

## Slide 24
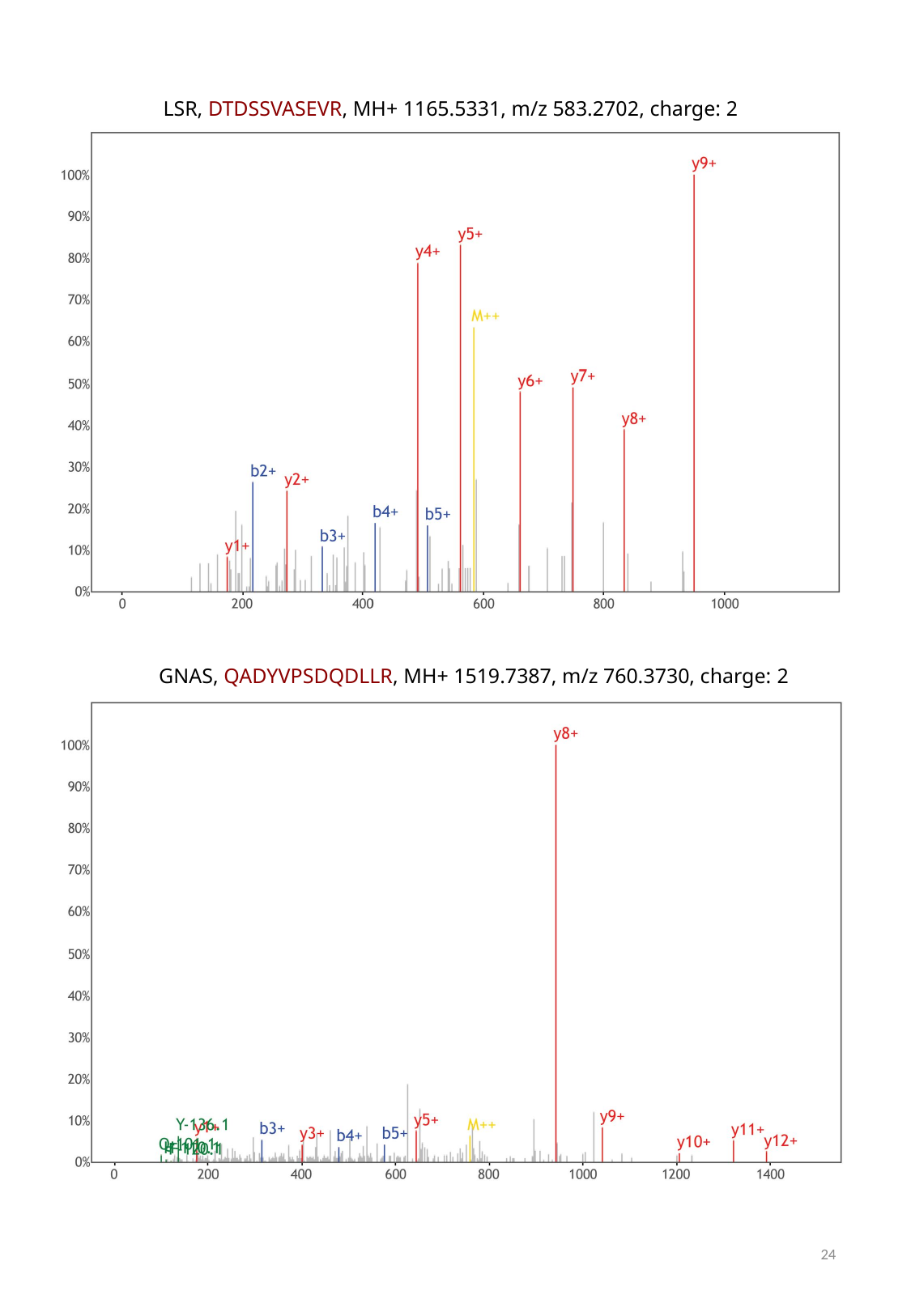

LSR, DTDSSVASEVR, MH+ 1165.5331, m/z 583.2702, charge: 2
GNAS, QADYVPSDQDLLR, MH+ 1519.7387, m/z 760.3730, charge: 2
24

## Slide 25
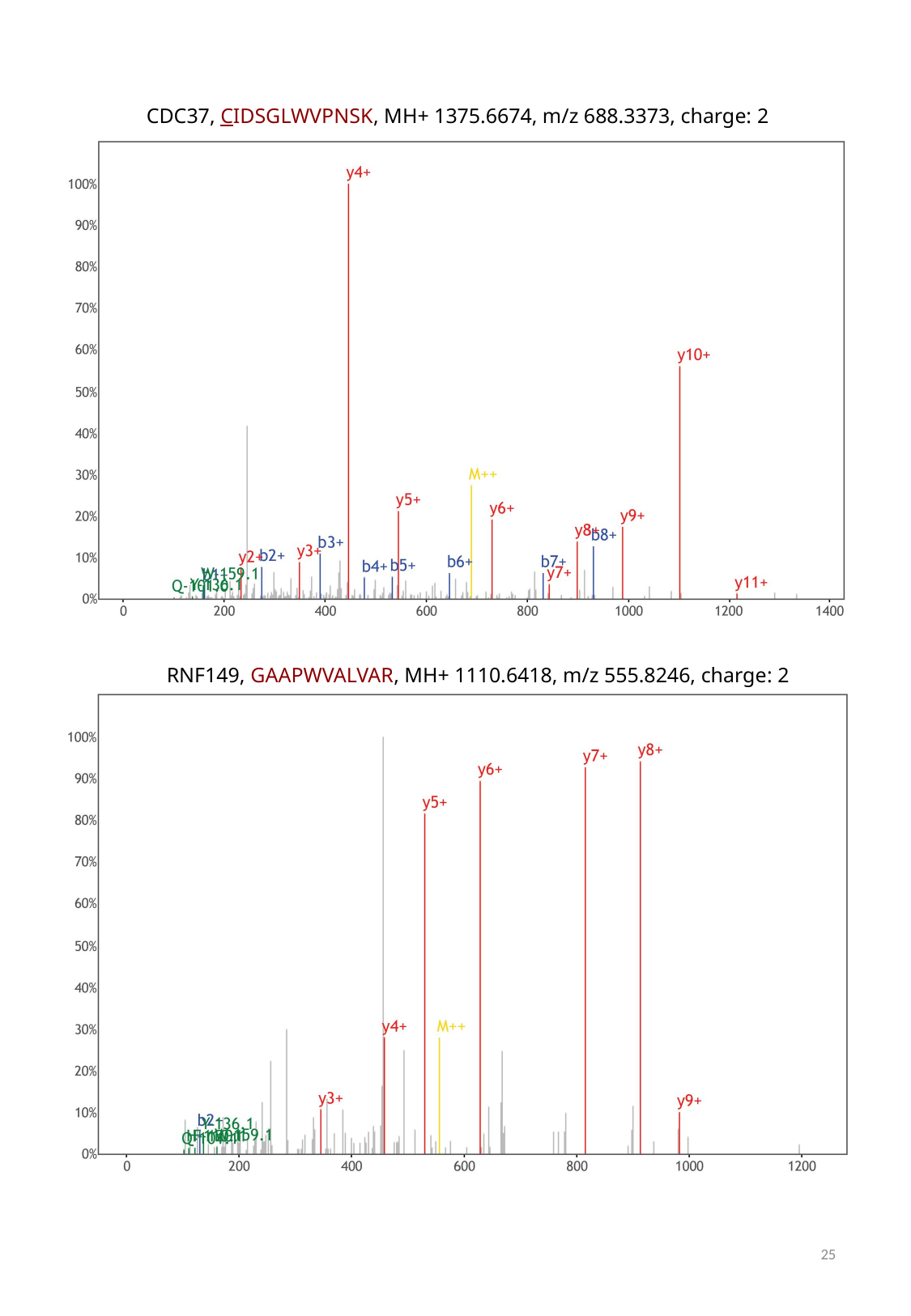

CDC37, CIDSGLWVPNSK, MH+ 1375.6674, m/z 688.3373, charge: 2
RNF149, GAAPWVALVAR, MH+ 1110.6418, m/z 555.8246, charge: 2
25

## Slide 26
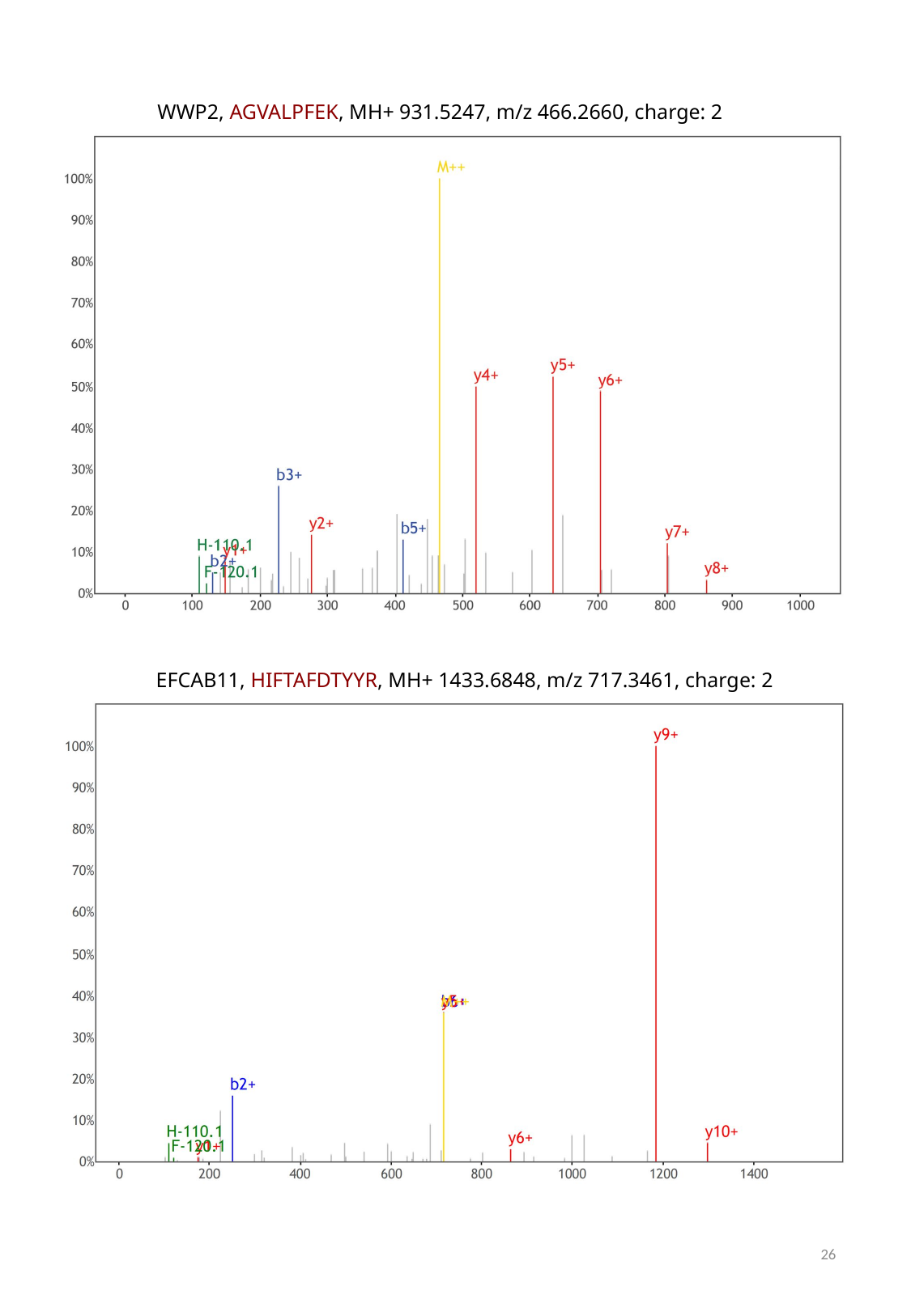

WWP2, AGVALPFEK, MH+ 931.5247, m/z 466.2660, charge: 2
EFCAB11, HIFTAFDTYYR, MH+ 1433.6848, m/z 717.3461, charge: 2
26

## Slide 27
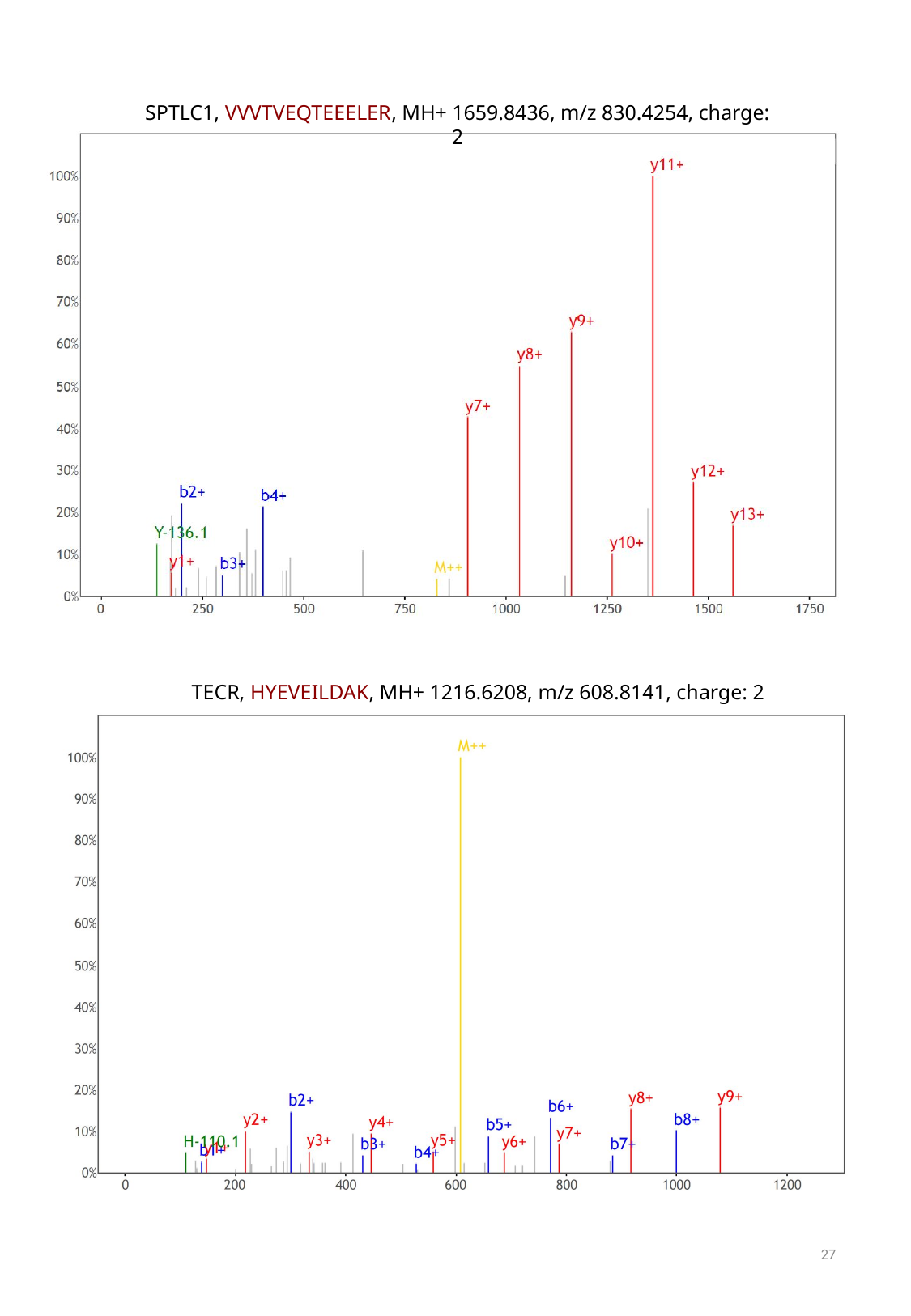

SPTLC1, VVVTVEQTEEELER, MH+ 1659.8436, m/z 830.4254, charge: 2
TECR, HYEVEILDAK, MH+ 1216.6208, m/z 608.8141, charge: 2
27

## Slide 28
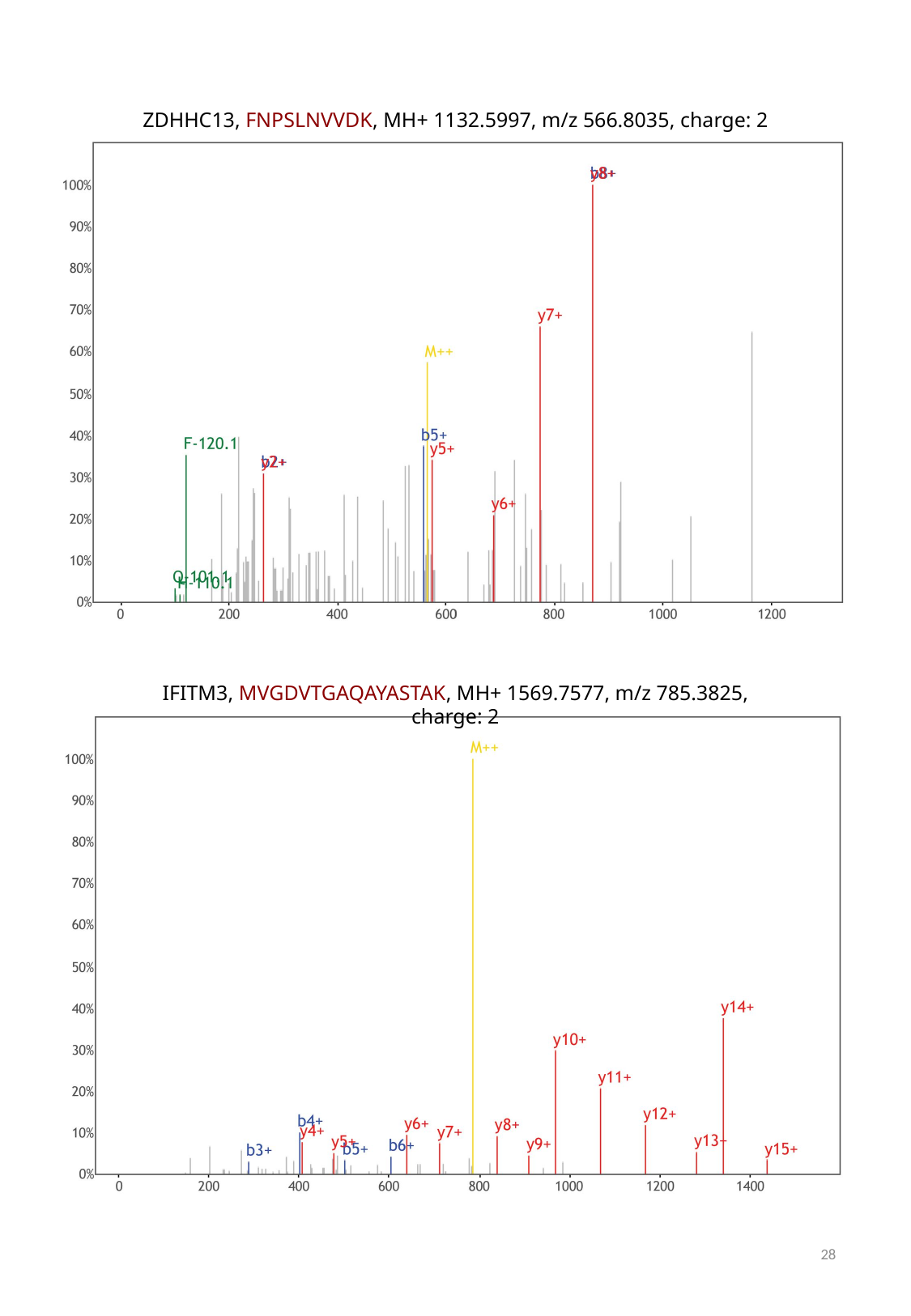

ZDHHC13, FNPSLNVVDK, MH+ 1132.5997, m/z 566.8035, charge: 2
IFITM3, MVGDVTGAQAYASTAK, MH+ 1569.7577, m/z 785.3825, charge: 2
28

## Slide 29
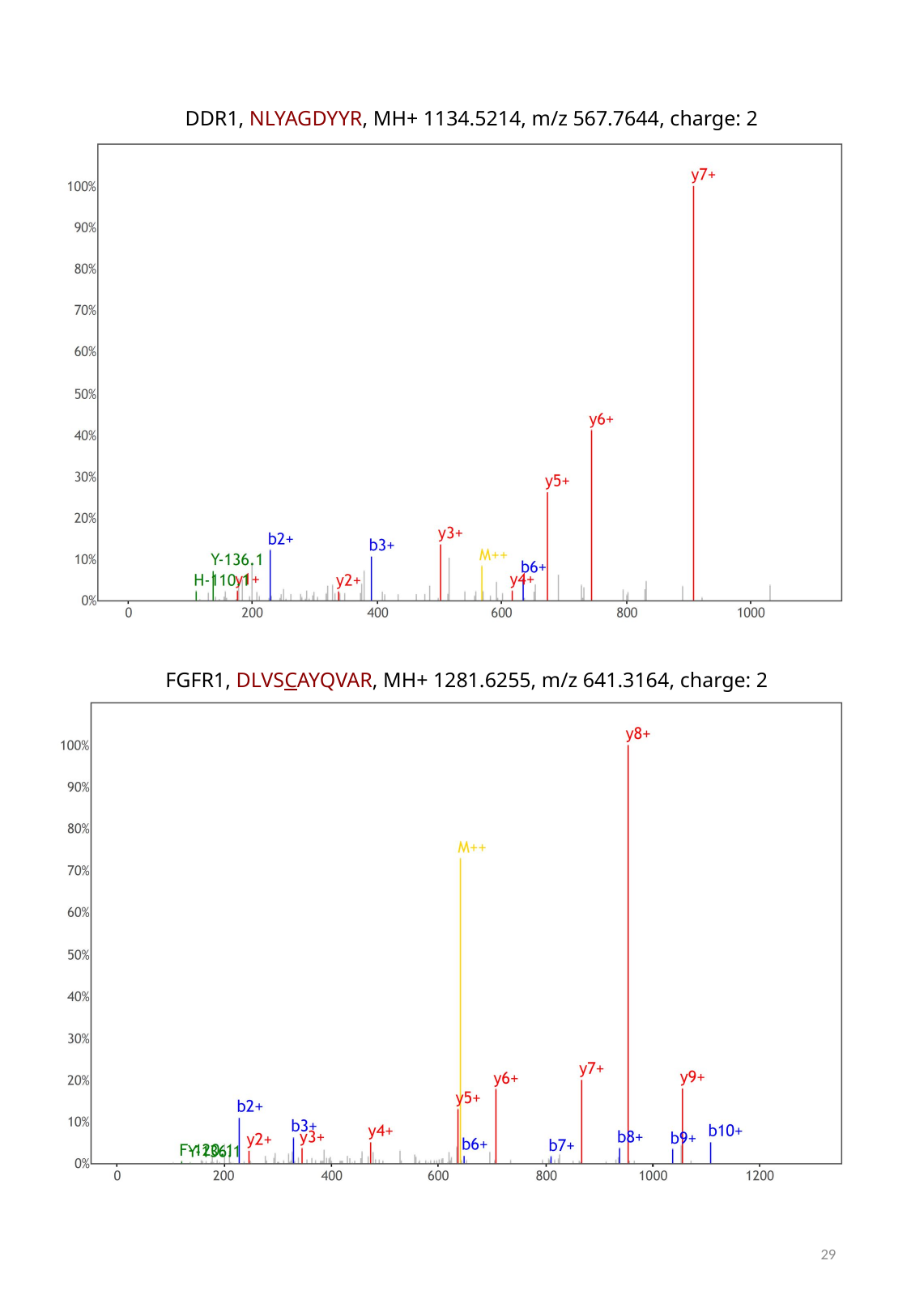

DDR1, NLYAGDYYR, MH+ 1134.5214, m/z 567.7644, charge: 2
FGFR1, DLVSCAYQVAR, MH+ 1281.6255, m/z 641.3164, charge: 2
29

## Slide 30
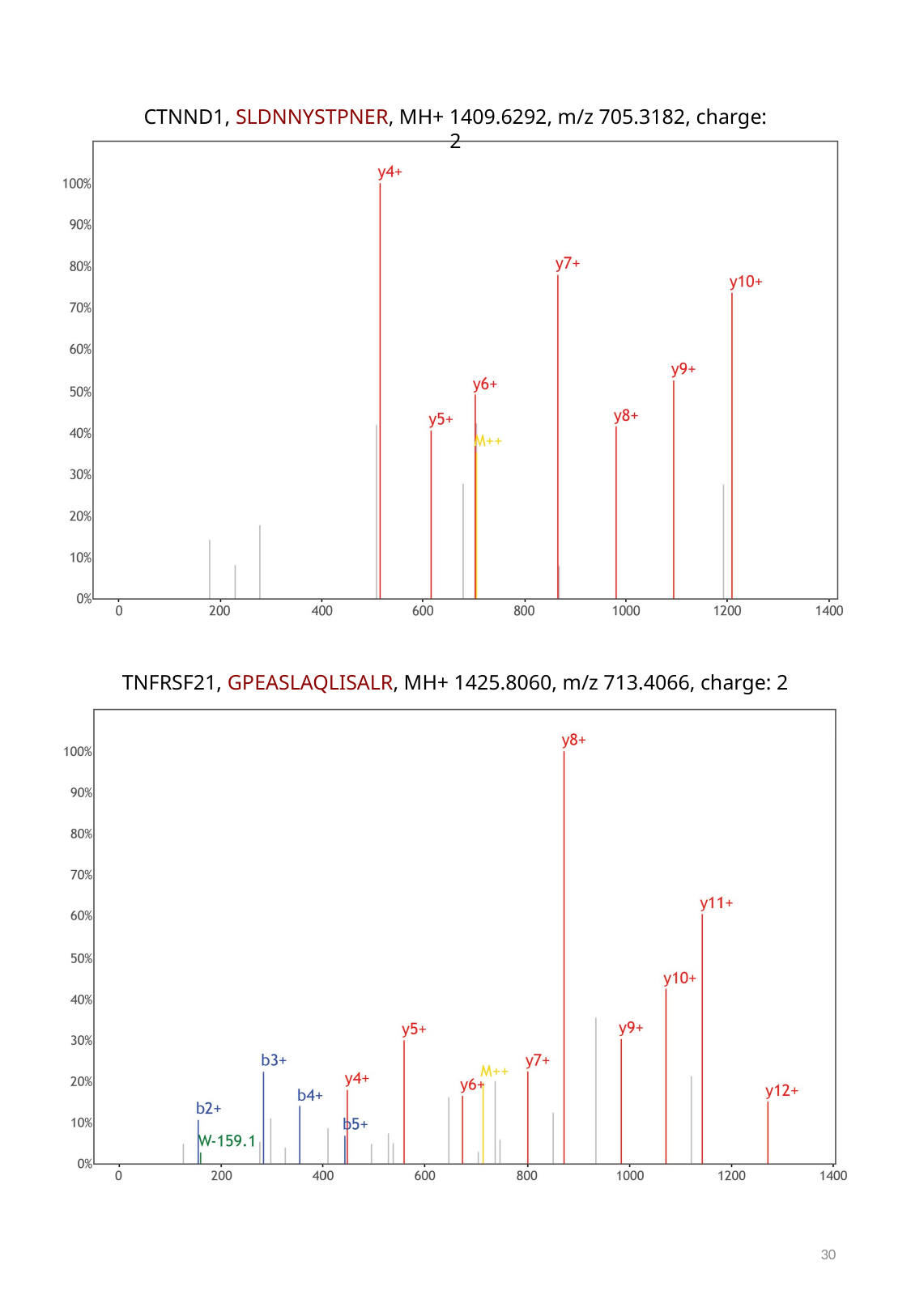

CTNND1, SLDNNYSTPNER, MH+ 1409.6292, m/z 705.3182, charge: 2
TNFRSF21, GPEASLAQLISALR, MH+ 1425.8060, m/z 713.4066, charge: 2
30

## Slide 31
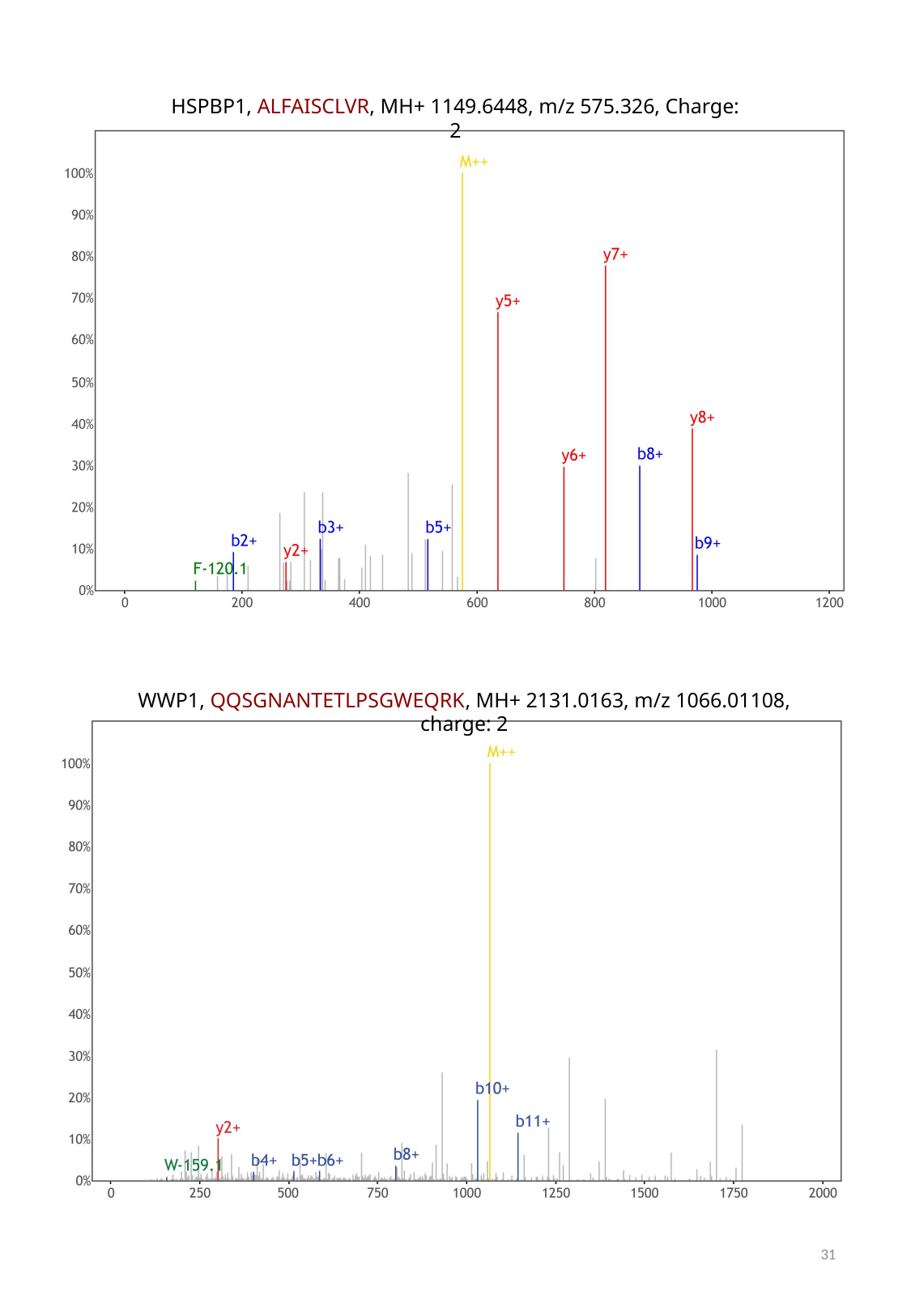

HSPBP1, ALFAISCLVR, MH+ 1149.6448, m/z 575.326, Charge: 2
WWP1, QQSGNANTETLPSGWEQRK, MH+ 2131.0163, m/z 1066.01108, charge: 2
31
